# Supplementary material for: Early childhood parent-reported speech problems in small and large for gestational age term-born and preterm-born infants: a cohort study
Source: BMJ Open. 2023 Apr 27;13(4):e065587. doi: 10.1136/bmjopen-2022-065587 (PMC10151836; doi:10.1136/bmjopen-2022-065587)
Supplement: Supplementary data [file bmjopen-2022-065587supp001.pdf]

Ethics protocol Version 1.1 5th November 2012

Respiratory and neurological outcomes in children born preterm study (RANOPs)

Research team:

Surname	First name	Title	Post held
Edwards	Martin	Dr	Clinical Research Fellow<br>Cardiff University
Kotecha	Sailesh	Professor	Professor of Child Health (CI for study)<br>Cardiff University
Dunstan	Frank	Professor	Professor of Medical Statistics<br>Cardiff University
Henderson	John	Professor	Professor of Paediatric Respiratory Medicine<br>Bristol University
Watkins	John	Dr	Medical Statistician<br>Cardiff University
John	Gareth	Mr	Improvement and Innovations Manager<br>NWIS
Richards	Louise	Mrs	Information Technology Support<br>NWIS
Morris	Geraint	Dr	Consultant Paediatrician *(Local PI)*<br>Abertawe Bro Morgannwg University Health Board
Papworth	Sue	Dr	Consultant Neonatologist *(Local PI)*<br>Aneurin Bevan Health Board
Stutchfield	Peter	Dr	Consultant Paediatrician *(Local PI)*<br>Betsi Cadwaladr University Health Board
Al-Muzaffar	Iyad	Dr	Consultant Neonatal Paediatrician *(Local PI)*<br>Cwm Taf Health Board
Pitchaikani	Prem	Dr	Consultant Paediatrician *(Local PI)*<br>Hywel Dda Health Board
Prosser	Ingrid	Dr	Consultant Community Paediatrician *(Local PI)*<br>Powys Teaching Health Board

1. *Background:*

Babies born late preterm, between 33 and 36 weeks gestation (6-7% of all UK births), have a greater risk of developing breathing problems than previously appreciated. Our research group has recently shown that late preterm born 8-9 year olds have worse lung function than similarly aged children born at term but their lung function improves by 14-17 years of age (http://www.bbc.co.uk/news/uk-wales-south-east-wales-15071922).[1] In 2009 in England and Wales there were 706,248 live-births and over 42,000 were born late preterm.[2,3] This group of children born late preterm tends to be treated the same as children born full term however there may be a need to monitor this group of children more closely.

Definition of terms:

Gestational groups - ≤32 weeks gestation = extremely preterm
- 33-36 weeks gestation = late preterm
- ≥37 weeks gestation = term

1.1 Research Question

We hypothesise that children born extremely preterm (23-32 weeks gestation) and late preterm (33 to 36 weeks gestation):

(a) have increased respiratory symptoms and disease,

(b) have increased neurodevelopmental problems,

(c) have increased health care utilisation, mainly due to respiratory morbidity

in infancy, in the pre-school and early school years, when compared to age-matched term-born children (control group).

Jee G, *et al. BMJ Open* 2023; 13:e065587. doi: 10.1136/bmjopen-2022-065587

1.2 Methods

Study Design: The morbidity and health utilisation of late preterm infants

In Wales, the average number of live-born deliveries over the last 5 years is 34,464 per annum.[2] Approximately 2,000 each year are born prematurely with 500 born extremely preterm and 1,500 late preterm.[2]

AWPS data: Total number of survivors at 1 year of age per year of birth and gestational age in Wales						
Year of birth	2003	2005	2007	2009	2010	2011
Gestational age						
23-32 weeks	436	507	503	471	513	492
33-36 weeks	1781	1719	1976	1980	1926	1913
37-43 weeks	26448	30063	31545	31933	33277	32819
Other/unknown	2303	226	279	323	171	334
Total	30968	32515	34303	34707	35887	35558

For late preterm infants, we will address:

(a) respiratory morbidity,

(b) neurodevelopmental problems and

(c) health care utilisation, especially due to respiratory reasons, via a questionnaire study and also the well-linked health databases in Wales with the collaboration of local neonatologists.

The data will be compared to term born and extremely preterm-born groups. Our team, including Professor John Henderson as well as Professor Frank Dunstan, has great expertise in questionnaire studies assessing health of children and adults.[4,5] A modified ISAAC questionnaire focussing on respiratory symptoms (rather than allergy) will be used to assess respiratory health including wheezing, drug usage and physical activity as well as visits to hospitals and general practitioners in school-aged children[6] and a validated questionnaire by Powell et al will be used for preschool children .[7,8] The questionnaires will be used to assess respiratory health and for any neurodevelopmental difficulties in a cross-sectional survey of children aged 1, 2, 3, 5, 7 and 9 years. The questionnaires will allow us to assess the impact of late preterm birth on respiratory outcomes, developmental delay and health service utilisation. The questionnaires will be age-appropriate (one for <5 years of age and another for 5-9 years of age). The two age-appropriate questionnaires are based on previous extensively validated questionnaires for both respiratory symptoms and neurodevelopmental outcomes in both preschool and school-aged children.[6-11]

We shall send 2,000 questionnaires with an invitation letter and parent information sheet for each age-group for the preterm groups (including 1,500 late preterm- and 500 extremely preterm-born children) together with a similar number for age-matched term controls. The matching of the term controls will be based on sex, place of birth and chronological age (DOB), although in the analysis of the data, the corrected postnatal age (this is the age a premature baby would be if born at term) of the preterm infants will be taken into account as a potential confounding factor. The total of 24,000 questionnaire packs will be sent via the NHS Wales Informatics Service (NWIS), which have expertise for identifying subjects from the NHS administrative register and then providing DST, the mailing house with a database to send out the questionnaires. Furthermore, children who have moved from the area or have died are identified and excluded, by using data from the All Wales Perinatal Survey (AWPS) which is directed by Professor Sailesh Kotecha with Professor Frank Dunstan a member of the steering committee.[2] AWPS maintains data on children who die before their first birthday, and this data will be used along with the Welsh Demographics Service, which also holds information on children who have passed away.

Prior to any reminder packs being sent out to the families, NWIS will be up-dated of all families who have returned a questionnaire pack. NWIS will then up-date the contact data base and also repeat the safe guard of who has died, and then send the new data base of contact information to DST, who will send out the reminder packs (1st after 1 month from the initial mailing date and 2nd after 3-4 months).

Secondly, using linked databases (including the Patient Episode Database for Wales, PEDW and the National Community Child Health Database, NCCHD and the Welsh Demographics Service, WDS) for hospital episodes, we will identify the general health service usage of these children. For those children with a returned, signed (consented) questionnaire we will be able to link the hospital data to their completed questionnaire to allow for more accurate analysis. The information gathered from PEDW, NCCHD and WDS will also contain data on all children less than 10 years of age in Wales (anonymised) and this will allow us to determine the representativeness of the sample returning questionnaires. The social status of a family will be determined by location using the welsh index for multiple deprivation child index score 2011 (taken from WDS).

The linked cross sectional data at 5 age points will be summarised in terms of rates of wheezing, rates of hospital admissions, etc. The exploration of the data will be hypothesis led to investigate if being born late preterm has an effect on the health outcome of children when compared to term born controls. The health outcomes will include hospital admissions, visits to GP, wheezing episodes, use of prescribed medication, doctor diagnosed conditions, long term disability and activity levels. The formal analysis will use general linear models to compare rates between different gestational age groups adjusting for confounders such as social class, maternal smoking during pregnancy, birth weight, gender etc.

Sample size: For a question with binary response (e.g. symptom present or absent), assuming a 50-60% response rate to the questionnaire, we will have 95% power for identifying a difference between the preterm- and term-born children if the true symptom rate is 15% in preterm and 10% in term children. We have estimated the response rates based on previous similar studies in Wales.[4, 12]

2. NWIS collaboration

We have already liaised with NWIS and shall ask their team to identify children born between 1/01/2003 to 31/12/2011. NWIS will identify all preterm infants (<37 weeks of gestation at birth) for each age group (1,2,3,5,7 & 9 years of age) using the national database (NCCHD and WDS). NWIS will then select the term controls by identifying gender matched children born on the same day and in the same area (hospital or midwifery led unit) as each preterm infant. So there will be approximately 2,000 term controls for each age group. For each age (1,2,3,5,7 and 9 years of age) there will be three groups; children born at full term (37-43 weeks of gestation), children born late preterm (33-36 weeks of gestation) and children born extremely preterm (≤32 weeks of gestation). Each child will be assigned a study number based on their DOB/gestational age/a unique reference number.

The information obtained from the health databases (WDS, NCCHD and PEDW) for each child will include the child's name, current address, gestational age, DOB, birth weight, breast feeding at birth and at 8 weeks of age, welsh index deprivation score, hospital admissions of the last year and discharge diagnosis. The postcode for the current address will be used to identify the local health board (so that the appropriate local PI is identified on the invitation letter being sent to the family). This information will need to be checked to remove any children that have died via AWPS and the WDS database. This will involve AWPS sending data to NWIS with the name, DOB and address of each child that has passed away over the last 10 years (1/1/03 to 31/12/12). ***None of this data will be made available to the research team.*** NWIS will supply DST, the mailing house with a study

database split into two sections (> 5 years of age and > 5 years of age). The database will contain the study ID number, name, address and local health board of each study participant. Data will be transferred between NWIS and DST via File Transfer Protocol Secure (FTPS).

DST will divide each data set into 7 sections according to the local health board in which the child resides. DST will then print the individual questionnaires (including study ID numbers), invitation letters including the family address and signed with the appropriate LHB with details of the local PI, and information sheets and place these in envelopes with windows that allow the letter to show the family address, and these packs will then be sent by royal mail to the families. Stamped addressed envelopes (to our research department) will be included within the study pack. DST also provide a further data health check, informing us of any duplicates and of families who may have moved, thus avoiding unnecessary/inappropriate mailing. ***No personal data will be released by NWIS to the research team at this stage.***

NWIS will send study ID numbers with the child's gestational age to the research team, so that when questionnaires are returned the correct gestational age for the child can be confirmed. Families will be given 4-8 weeks to return the questionnaires. A list of families who have returned questionnaires (by study ID numbers) will be sent to NWIS and they will send further information on each child that has a signed (consented) questionnaires, to include birth weight, antenatal details, hospital of birth, hospital admissions and discharge diagnosis over the last 12 months.

NWIS, following a re-check of the health data base for any deaths, will create a list of any families that have not returned a questionnaire. This up-dated study database will be given to DST and the first reminder pack will be sent to these families at 1 month after the initial pack was sent out. If the response rate remains low then this process will be repeated with a second remainder pack being sent after 3-4 months, when the research team will give NWIS a list of all families who have returned questionnaires and NWIS will collate an up-dated list, having removed any families where a child has died in the interim period since last checking. This up-dated study database will be given to DST and the second reminder pack will be sent to those families who have not yet responded.

Questionnaires will be returned to the Department of Child Health at Cardiff University and processed by automated scanners and specialised software (Remark Office OMR 8). The questionnaires will be coded by the software, which also identifies any problems such as missing information. Any problems detected by the software will be reviewed by the research team. This will form part of the data quality control checks. Anonymised data will be available for the whole cohort to allow us to determine the representativeness of the responders. The returned questionnaires will be processed by Dr Martin Edwards and all data stored securely in the research office at the Department of Child Health based at the University Hospital of Wales, Heath Park, Cardiff. By returning the completed questionnaires it will be assumed that the family is consenting to take part in the study. If the questionnaire form is signed the family will be consenting to be contacted with regards to clarifying any issues with the returned questionnaire; contacted in the future to take part in further research in this area; and for access to patient identifiable information in the health databases to link hospital admission and GP records information with that gathered from the questionnaires. It is clearly stated in the information leaflet what will happen with the data collected.

3. Ethical approval

The project has ethical and R&D approval from within Wales. The global governance check reference is **IRAS 91349** and the Ethics reference is **12/WA/0155.** The sponsor for the project is Cardiff University (**SPON1038-11**).

4. Timescale

We aim to mail the questionnaires between January and February 2013. We shall allow the families 6 – 8 weeks to respond initially and send a second mailing of questionnaires between April and May 2013 and if necessary a third mailing of questionnaires in between July and August 2013, dependent upon the response rate. Our expectation is to reach a 50 – 60% response rate as has been achieved in many recent questionnaire studies.[4, 12-14]

5. Resources

The study is currently funded by Departmental funds and research grant funding is being sought from several charities that support medical research – decision expected Jan/Feb 2013.

6. Documents

i. Letter of invitation to parents – 1 side of A4 paper
ii. Questionnaire for preschool children (<5 years old) – 4 sides of A4 paper
iii. Questionnaire for school aged children (5-9 years old) – 4 sides of A4 paper
iv. Information sheet for families – 2 sides of A4 paper
v. Flow diagram of research protocol

7. References

1. Kotecha, S.J., et al., *Effect of late preterm birth on longitudinal lung spirometry in school age children and adolescents.*Thorax.2012;67:54-61.
2. Kotecha S, et al. All Wales Perinatal Survey – Annual Report 2009. Cardiff University 2011.
3. Office of National Statistics (2009).
4. Burr ML, Wat D, Evans C, Dunstan FD, Doull IJ; BTS Research Committee. Asthma prevalence in 1973, 1988 and 2003. Thorax. 2006;61:296-9
5. Thomas H, et al. Mental health and quality of residential environment. Br J Psychiatry. 2007;191:500-5
6. Asher MI, et al. International study of asthma and allergies in childhood (ISAAC): Rationale and methods. Eur Respir J 1995;8:483-491.
7. Powell CV, McNamara P, Solis A, Shaw NJ. A parent completed questionnaire to describe the patterns of wheezing and other respiratory symptoms in infants and preschool children. *Archives of Disease in Childhood* 2002;87(5):376-9.
8. Trinick, R., K.W. Southern, and P.S. McNamara, *Assessing the Liverpool Respiratory Symptom Questionnaire in children with cystic fibrosis.* The European respiratory journal : official journal of the European Society for Clinical Respiratory Physiology, 2012;39(4):899-905.
9. Greenough A, Giffin FJ, Yuksel B. Respiratory morbidity in preschool children born prematurely. Relationship to adverse neonatal events. *Acta Paediatr* 1996;85(7):772-7.
10. Greenough A, Limb E, Marston L, Marlow N, Calvert S, Peacock J. Risk factors for respiratory morbidity in infancy after very premature birth. *Arch Dis Child Fetal Neonatal Ed* 2005;90(4):F320-3.
11. Jones H, Guildea Z, Stewart, J & Cartlidge, P.The health status questionnaire: Achieving concordance with published disability criteria. Archives of Disease in Childhood, 2002. **86**(1): p. 15-20.
12. Fox, R. and S. Minchom, *Parental experiences of the newborn hearing screening programme in Wales: a postal questionnaire survey.* Health expectations : an international journal of public participation in health care and health policy, 2008;11(4):376-83.
13. Leonardi, N.A., et al., *Validation of the Asthma Predictive Index and comparison with simpler clinical prediction rules.* The Journal of allergy and clinical immunology, 2011; 127(6):1466-72 e6.
14. Turner, S., et al., *First- and second-trimester fetal size and asthma outcomes at age 10 years.* American Journal of Respiratory and Critical Care Medicine, 2011;184(4);407-13.
