# Supplementary material for: Early childhood parent-reported speech problems in small and large for gestational age term-born and preterm-born infants: a cohort study
Source: BMJ Open. 2023 Apr 27;13(4):e065587. doi: 10.1136/bmjopen-2022-065587 (PMC10151836; doi:10.1136/bmjopen-2022-065587)
Supplement: Supplementary data [file bmjopen-2022-065587supp002.pdf]

Using a **PENCIL** fill in the circles like this ○ → ●

DO NOT tick or cross the circles.

An eraser can be used to rectify mistakes.

Study No: **12351**

Health questionnaire for preschool children (<5 years old)

Today's date ____________________2012

Person completing the questionnaire: Mother ○ Father ○ Other ○

Date of birth of child: ______/________/________

1. Has your child ever had wheezing or whistling in their chest at any time in their life?

Yes ○ No ○ Unsure ○

IF YOU HAVE ANSWERED "NO" PLEASE GO TO QUESTION 4.

2. In the last three months , during the day time (i.e. when awake) does your child:

	Not at all	a few days	some days	most days	every day
a. Wheeze:	○	○	○	○	○
b. Cough:	○	○	○	○	○
c. Short of breath:	○	○	○	○	○
d. Rattly chest:	○	○	○	○	○
e. Snuffly:	○	○	○	○	○

3. In the last three months , during the night time (i.e. when asleep) does your child:

	Not at all	a few nights	some nights	most nights	every night
a. Wheeze:	○	○	○	○	○
b. Cough:	○	○	○	○	○
c. Short of breath:	○	○	○	○	○
d. Rattly chest:	○	○	○	○	○
e. Snore:	○	○	○	○	○

4. How many colds has your child had in the last three months?

None ○ 1 to 3 ○ more than 3 ○ always has a cold ○

5. When my child has had a cold in the last three months, s/he has a:

	Not at all	a few colds	some colds	most colds	every cold
a. Wheeze:	○	○	○	○	○
b. Cough:	○	○	○	○	○
c. Short of breath:	○	○	○	○	○
d. Rattly chest:	○	○	○	○	○

Jee G, *et al. BMJ Open* 2023; 13:e065587. doi: 10.1136/bmjopen-2022-065587

Study No: **12351**

e. Snuffly:	○	○	○	○	○

6. When my child does NOT have a cold, in the last three months, s/he has a:

	Not at all	a few days	some days	most days	every day
a. Wheeze:	○	○	○	○	○
b. Cough:	○	○	○	○	○
c. Short of breath:	○	○	○	○	○
d. Rattly chest:	○	○	○	○	○
e. Snuffly:	○	○	○	○	○

7. When my child has been more active (e.g. crawling, walking or when excited), in the last three months, s/he has a:

	Not at all	a few days	some days	most days	every day
a. Wheeze:	○	○	○	○	○
b. Cough:	○	○	○	○	○
c. Short of breath:	○	○	○	○	○
d. Rattly chest:	○	○	○	○	○

8. Has your child ever been diagnosed with asthma by a doctor?

Yes ○ No ○

9. Is there a family history of

	Yes	No
a. Asthma	○	○
b. Eczema	○	○
c. Hayfever	○	○
d. Allergies	○	○

10. In the last 12 months, has your child ever used any regular asthma inhalers (pumps) or medicines?

Yes ○ No ○

If yes, please provide the name (or colour of the pump) with details of how often used:

____

____

11. In the last 12 months, has your child had any chest infections?

Yes ○ No ○

12. In the last 12 months, how many chest infections has your child had?

Jee G, *et al. BMJ Open* 2023; 13:e065587. doi: 10.1136/bmjopen-2022-065587

Study No: 12351

None ◯ 1 to 3 ◯ 4 to 10 ◯ more than 10 ◯

13. In the last 12 months, how many courses of antibiotics has your child had?
None ◯ 1 to 3 ◯ 4 to 10 ◯ more than 10 ◯

14. In the last 12 months, how many admissions (overnight or longer) has your child had to hospital for breathing problems?
None ◯ 1 to 3 ◯ 4 to 10 ◯ more than 10 ◯

14a. Does the child's mother smoke cigarettes? Yes ◯ No ◯

If yes, how many per day? 1 to 10 ◯ 11 to 20 ◯ more than 20 ◯

If the child's mother smokes did she smoke during the pregnancy? Yes ◯ No ◯

14b. Does the child's father smoke cigarettes? Yes ◯ No ◯

If yes, how many per day? 1 to 10 ◯ 11 to 20 ◯ more than 20 ◯

15. Do any other household members smoke cigarettes? Yes ◯ No ◯

If yes, how many per day for the whole household? 1 to 10 ◯
11 to 20 ◯
more than 20 ◯

(please add up all the cigarettes which are smoked by everyone living in the same household including the mother).

16. a . Has your child ever been diagnosed with any breathing problems (e.g. asthma, CF, TB etc.)?
Yes ◯ No ◯
If yes, please provide some details:
____
____

16. b . Has your child ever been diagnosed with any other conditions (e.g. diabetes, epilepsy etc.)?
Yes ◯ No ◯
If yes, please provide some details:
____
____

17. Is your child on any medication (please list all medicines your child is being given)?
Yes ◯ No ◯
If yes, please provide some details:
____
____

18. Does your child take part in any physical activity every week such as dancing, swimming, cycling ?
Yes ◯ No ◯
If yes, please provide some details of how often and for how long:

Jee G, *et al. BMJ Open* 2023; 13:e065587. doi: 10.1136/bmjopen-2022-065587

Study No: 12351

____

____

19. Does your child have any problems with moving?
Yes ○ No ○
If yes, please provide some details:

20. Does your child have any problems with using their hands?
Yes ○ No ○
If yes, please provide some details:

21. Does your child have any problems with their speech?
Yes ○ No ○
If yes, please provide some details:

22. Does your child have any problems with their vision?
Yes ○ No ○
If yes, please provide some details:

23. Does your child have any problems with their hearing?
Yes ○ No ○
If yes, please provide some details:

24. Does your child have any problems with their behaviour?
Yes ○ No ○
If yes, please provide some details:

25. Does your child have any learning difficulties?
Yes ○ No ○
If yes, please provide some details:

Thank you for filling in the form. The following section asks how you are happy for us to use the data or to contact you:

(A) If we need to clarify some of your answers, would you be willing to be contacted?

Yes ○ No ○ Please initial the box here ☐

Address, Telephone number &/or email:

Jee G, *et al. BMJ Open* 2023; 13:e065587. doi: 10.1136/bmjopen-2022-065587

Study No: 12351

(B) Most admissions and GP visits in Wales are stored in computer databases called NCCHD or PEDW. As part of this study, we would also like to study how children in Wales have used their GPs or have had admission to hospitals. Would you be happy for us to use your son's or daughter's records on these databases?

Yes ○ No ○ Please initial the box here ☐

(C) We may plan similar studies in the future, would you be willing to be contacted in the future?

Yes ○ No ○ Please initial the box here ☐

Name of Child	Name of Parent/Guardian	Date	Signature

Thank you very much for taking the time to fill in the form and for contributing to our research. Could you please send the form to:

Dr Martin Edwards,
Room UGT156
Department of Child Health
School of Medicine
Cardiff University
University Hospital of Wales
Heath Park
Cardiff CF14 4XN

In the enclosed self-addressed envelope.

Jee G, *et al. BMJ Open* 2023; 13:e065587. doi: 10.1136/bmjopen-2022-065587

Using a **PENCIL** fill in the circles like this

○ ⟶ ●

DO NOT tick or cross the circles.

An eraser can be used to rectify mistakes.

Study No:

Health questionnaire for school age children (>5 years old)

Today's date ____________________2012

Person completing the questionnaire: Mother ○ Father ○ Other ○

Date of birth of child: ______/_________/________

1. Has your child ever had wheezing or whistling in their chest at any time in the past?

   Yes ○ No ○ Unsure ○

IF YOU HAVE ANSWERED "NO" PLEASE GO TO QUESTION 10.

2. Has your child had wheezing or whistling in the chest in the last 12 months?

   Yes ○ No ○ Unsure ○

IF YOU HAVE ANSWERED "NO" PLEASE GO TO QUESTION 10.

3. How many attacks of wheezing has your child had in the last 12 months?

   None ○ 1 to 3 ○ 4 to 12 ○ more than 12 ○

4. In the last 12 months, how often, on average, has your child's sleep been disturbed due to wheezing?

   Never woken with wheezing ○

   Less than one night per week ○

   One or more nights per week ○

5. In the last 12 months, has wheezing ever been severe enough to limit your child's speech to only one or two words at a time between breaths?

   Yes ○ No ○

6. Has a doctor ever told you that your child has asthma?

   Yes ○ No ○

7. In the last 12 months, has your child's chest sounded wheezy during or after exercise?

   Yes ○ No ○

8. In the last 12 months, has your child ever used any regular asthma inhalers (pumps) or medicines?

   Yes ○ No ○

   If yes, please provide the name (or colour of the pump) with details of how often used:

   ____

   ____

Jee G, *et al. BMJ Open* 2023; 13:e065587. doi: 10.1136/bmjopen-2022-065587

Study No:

9. In the last 12 months, has your child had a dry cough at night, apart from a cough associated with a cold or chest infection?
   Yes ◯ No ◯

10. A. Does the child's mother smoke cigarettes?
   Yes ◯ No ◯
   If yes, how many per day? 1 to 10 ◯ 11 to 20 ◯ more than 20 ◯

   If the child's mother smokes did she smoke during the pregnancy? Yes ◯ No ◯

10.B. Does the child's father smoke cigarettes?
   Yes ◯ No ◯

   If yes, how many per day? 1 to 10 ◯ 11 to 20 ◯ more than 20 ◯

11. Do any other household members smoke cigarettes? Yes ◯ No ◯

   If yes, how many per day for the whole household? 1 to 10 ◯
   11 to 20 ◯
   more than 20 ◯
   (please add up all the cigarettes which are smoked by everyone living in the same household including the mother).

12. In the last 12 months, has your child had any chest infections?

   Yes ◯ No ◯

13. In the last 12 months, how many chest infections has your child had?

   None ◯ 1 to 3 ◯ 4 to 10 ◯ more than 10 ◯

14. In the last 12 months, how many courses of antibiotics has your child had?

   None ◯ 1 to 3 ◯ 4 to 10 ◯ more than 10 ◯

15. In the last 12 months, how many admissions (overnight or longer) has your child had to hospital for breathing problems?

   None ◯ 1 to 3 ◯ 4 to 10 ◯ more than 10 ◯

16. Is there a family history of

	Yes	No
a. Asthma	◯	◯
b. Eczema	◯	◯
c. Hayfever	◯	◯
d. Allergies	◯	◯

17. Has your child ever been diagnosed with any breathing problems (e.g. asthma, CF, TB etc.)?

   Yes ◯ No ◯

   If yes, please provide some details:

   ____

   ____

Jee G, *et al. BMJ Open* 2023; 13:e065587. doi: 10.1136/bmjopen-2022-065587

Study No:

18. Has your child ever been diagnosed with any other conditions (e.g. diabetes, epilepsy etc.)?

    Yes ◯ No ◯

    If yes, please provide some details:

    ____

    ____

19. Is your child on any medication?

    Yes ◯ No ◯

    If yes, please provide some details:

    ____

    ____

20. Does your child take part in any physical activity such as dancing, cycling or swimming?

    Yes ◯ No ◯

    If yes, please provide some details of how often and for how long:

    ____

    ____

21. Does your child have any learning problems?

    Yes ◯ No ◯

    If yes, please provide some details:

    ____

    ____

22. Does your child have any problems with their behaviour?

    Yes ◯ No ◯

    If yes, please provide some details:

    ____

    ____

23. Does your child have an educational statement?

    Yes ◯ No ◯

    If yes, please provide some details:

    ____

    ____

24. Does your child have any problems with moving?

    Yes ◯ No ◯

    If yes, please provide some details:

    ____

    ____

Jee G, *et al. BMJ Open* 2023; 13:e065587. doi: 10.1136/bmjopen-2022-065587

Study No:

25. Does your child have any problems with writing or using their hands?

Yes ○ No ○

If yes, please provide some details:

____

26. Does your child have any problems with speech?

Yes ○ No ○

If yes, please provide some details:

____

____

27. Does your child have any problems with their vision?

Yes ○ No ○

If yes, please provide some details:

____

____

28. Does your child have any problems with their hearing?

Yes ○ No ○

If yes, please provide some details:

____

____

29. Does your child have any problems with feeding?

Yes ○ No ○

If yes, please provide some details:

____

____

Thank you for filling in the form. The following section asks how you are happy for us to use the data or to contact you:

(A) If we need to clarify some of your answers, would you be willing to be contacted?

Yes ○ No ○ Please initial the box here ☐

Address, Telephone number &/or email:

____

____

Jee G, *et al. BMJ Open* 2023; 13:e065587. doi: 10.1136/bmjopen-2022-065587

Study No:

(B) Most admissions and GP visits in Wales are stored in computer databases called NCCHD or PEDW. As part of this study, we would also like to study how children in Wales have used their GPs or have had admission to hospitals. Would you be happy for us to use your son's or daughter's records on these databases?

Yes ◯ No ◯ Please initial the box here ☐

(C) We may plan similar studies in the future, would you be willing to be contacted in the future?

Yes ◯ No ◯ Please initial the box here ☐

Name of Child	Name of Parent/Guardian	Date	Signature

Thank you very much for taking the time to fill in the form and for contributing to our research.
Could you please send the form to:

Dr Martin Edwards,
Room UGT156
Department of Child Health
School of Medicine
Cardiff University
University Hospital of Wales
Heath Park
Cardiff CF14 4XN

In the enclosed self-addressed envelope.

Jee G, *et al. BMJ Open* 2023; 13:e065587. doi: 10.1136/bmjopen-2022-065587
