# Supplementary material for: Early childhood parent-reported speech problems in small and large for gestational age term-born and preterm-born infants: a cohort study
Source: BMJ Open. 2023 Apr 27;13(4):e065587. doi: 10.1136/bmjopen-2022-065587 (PMC10151836; doi:10.1136/bmjopen-2022-065587)
Supplement: Supplementary data [file bmjopen-2022-065587supp003.pdf]

Supplement 3. Comparison of responders and non-responders in the original study, RANOPs.

**Measure**	**Responders** n=7,129	**Non-Responders** n=19,593	**P value**
Preterm	4264 (59.9%)	9080 (46.4%)	<0.001
Birthweight centile			0.606
<10th	1879 (9.6%)	666 (9.4%)	
10th-90th	5478 (76.9%)	15,091 (77.1%)	
>90th	976 (13.7%)	2603 (13.3%)	
Male Sex	3846 (54.0%)	10,659 (54.5%)	0.523
Living in most deprived 50%	2736 (39.5%)	10,286 (53.8%)	<0.001

Numbers are number (%) or mean (SD) as appropriate.
