# Supplementary material for: Early childhood parent-reported speech problems in small and large for gestational age term-born and preterm-born infants: a cohort study
Source: BMJ Open. 2023 Apr 27;13(4):e065587. doi: 10.1136/bmjopen-2022-065587 (PMC10151836; doi:10.1136/bmjopen-2022-065587)
Supplement: Supplementary data [file bmjopen-2022-065587supp004.pdf]

Supplement 4. Multiple imputation model

Imputation was based on the following distributions. 50 imputed sets of data were used and combined using Rubin's rules.[1]

Variable	Imputation command	% missing
Speech Problems	-	0 (0.0%)
Learning Difficulties	logit	24 (0.3%)
Behaviour Problems	logit	41 (0.6%)
Movement Problems	logit	13 (0.2%)
Hand Problems	logit	4 (0.1%)
Maternal Age (years)	ologit	779 (11.1%)
Sex	-	0 (0.0%)
Single/Multiple pregnancy	-	0 (0.0%)
Mode of Delivery	mlogit	2913 (41.6%)
WIMD measure	regress	200 (2.9%)
Smoking Status	Logit	279 (4.0%)
Gestational age at birth	-	0 (0.0%)
Age at survey (years)	ologit	1 (0.01%)
Birthweight centile	-	0 (0.0%)

1. Rubin DB. Inference and missing data. Biometrika. 1976;63:581-92.
