# Supplementary material for: Early childhood parent-reported speech problems in small and large for gestational age term-born and preterm-born infants: a cohort study
Source: BMJ Open. 2023 Apr 27;13(4):e065587. doi: 10.1136/bmjopen-2022-065587 (PMC10151836; doi:10.1136/bmjopen-2022-065587)
Supplement: Author's manuscript [file bmjopen-2022-065587.draft_revisions.pdf]

BMJ Open is committed to open peer review. As part of this commitment we make the peer review history of every article we publish publicly available.

When an article is published we post the peer reviewers' comments and the authors' responses online. We also post the versions of the paper that were used during peer review. These are the versions that the peer review comments apply to.

The versions of the paper that follow are the versions that were submitted during the peer review process. They are not the versions of record or the final published versions. They should not be cited or distributed as the published version of this manuscript.

BMJ Open is an open access journal and the full, final, typeset and author-corrected version of record of the manuscript is available on our site with no access controls, subscription charges or pay-per-view fees (http://bmjopen.bmj.com).

If you have any questions on BMJ Open's open peer review process please email info.bmjopen@bmj.com

BMJ Open

Birthweight and long-term speech problems as a marker of higher-level neurocognitive function across all gestational ages at birth: A cohort study

Journal:	*BMJ Open*
Manuscript ID	bmjopen-2022-065587
Article Type:	Original research
Date Submitted by the Author:	13-Jun-2022
Complete List of Authors:	Jee, Gabrielle; University of Wales Hospital, Paediatrics<br>Kotecha, Sarah; Cardiff University School of Medicine, Department of Child Health<br>Chakraborty, Mallinath; Cardiff University School of Medicine, Department of Child Health; University of Wales Hospital, Department of Neonatology<br>Kotecha, Sailesh; Cardiff University School of Medicine, Department of Child Health; University of Wales Hospital, Department of Neonatology<br>Odd, David; University Hospital of Wales, Department of Neonatology; Cardiff University School of Medicine, Department of Population Health
Keywords:	NEONATOLOGY, Developmental neurology & neurodisability < PAEDIATRICS, Neonatal intensive & critical care < INTENSIVE & CRITICAL CARE

SCHOLARONE™
Manuscripts

I, the Submitting Author has the right to grant and does grant on behalf of all authors of the Work (as defined in the below author licence), an exclusive licence and/or a non-exclusive licence for contributions from authors who are: i) UK Crown employees; ii) where BMJ has agreed a CC-BY licence shall apply, and/or iii) in accordance with the terms applicable for US Federal Government officers or employees acting as part of their official duties; on a worldwide, perpetual, irrevocable, royalty-free basis to BMJ Publishing Group Ltd ("BMJ") its licensees and where the relevant Journal is co-owned by BMJ to the co-owners of the Journal, to publish the Work in this journal and any other BMJ products and to exploit all rights, as set out in our licence.

The Submitting Author accepts and understands that any supply made under these terms is made by BMJ to the Submitting Author unless you are acting as an employee on behalf of your employer or a postgraduate student of an affiliated institution which is paying any applicable article publishing charge ("APC") for Open Access articles. Where the Submitting Author wishes to make the Work available on an Open Access basis (and intends to pay the relevant APC), the terms of reuse of such Open Access shall be governed by a Creative Commons licence – details of these licences and which Creative Commons licence will apply to this Work are set out in our licence referred to above.

Other than as permitted in any relevant BMJ Author's Self Archiving Policies, I confirm this Work has not been accepted for publication elsewhere, is not being considered for publication elsewhere and does not duplicate material already published. I confirm all authors consent to publication of this Work and authorise the granting of this licence.

For peer review only

Birthweight and long-term speech problems as a marker of higher-level neurocognitive function across all gestational ages at birth: A cohort study

Jee, Gabrielle[a], Kotecha, Sarah J.[b], Chakraborty, Mallinath [b,c], Kotecha, Sailesh[b,c], Odd, David[c,d]

[a]*Department of Paediatrics, University Hospital of Wales, Cardiff, UK*

[b]*Department of Child Health, School of Medicine, Cardiff University, Cardiff, UK*

[c]*Department of Neonatology, University Hospital of Wales, Cardiff, UK*

[d]*Department of Population Health, School of Medicine, Cardiff University, Cardiff, UK*

For peer review only

Corresponding author:

David Odd

Neuadd Meirionnydd, University Hospital of Wales, Heath Park, Cardiff, CF14 4YS

oddd@cardiff.ac.uk

+44(0)2920874000

Keywords:

Neonates, birthweight, neurodevelopmental outcome

Word count:

2500

ABSTRACT

Objective

To assess if small (SGA) or large for gestational age (LGA) infants have worse long-term speech problems as a marker of higher neurocognitive function compared to infants with appropriate birthweights across all gestational ages at birth.

Design

Cohort study.

Setting

Wales, United Kingdom.

Patients

7004 children with neurodevelopmental outcomes from the Respiratory and Neurological Outcomes of Children Born Preterm study (RANOPs) which recruited 7129 responders, born from 23 weeks of gestation onwards to mothers aged 18 to 50 years of age were eligible.

Interventions

Parent-reported single-answer questionnaires with free text for additional information were completed in 2013 to assess neurodevelopmental outcomes across all ages. No intervention was given.

Main outcome measures

The primary neurodevelopmental outcome was parentally reported speech problems adjusted for clinical and demographic confounders in SGA and LGA infants compared to those with appropriate birthweights.

Results

Median age at time of study was 5 years, range 2-10 years. Although the adjusted OR was 1.28 (0.91-1.80) for infants born SGA and OR 0.95 (0.69-1.29) for LGA infants, this failed to reach statistical significance that these subgroups of infants were more likely to have long-term speech problems compared to infants born appropriate for gestational age (AGA). This

For peer review only

For peer review only - http://bmjopen.bmj.com/site/about/guidelines.xhtml

study also found evidence suggesting potential long-term learning difficulties in infants born SGA (OR 1.73 (1.17-2.57)) and movement problems in infants born LGA (OR 1.83 (1.20-2.77)).

Conclusion

Infants born SGA or LGA do not have higher risks of speech problems in childhood when compared to infants with appropriate birthweights.

For peer review only

What is already known on this topic

- Birthweight is an important predictor of neonatal prognostic outcomes
- Certain subgroups i.e., low birthweight and prematurity or excessive birthweight in term infants are associated with increased risk of immediate adverse outcomes which may possibly persist into adulthood on the cardiometabolic system
- Some established factors such as maternal diabetes, smoking during pregnancy and multiple births can influence birthweight

What this study adds

- Infants born small or large for gestational age are not more likely than those born appropriate for gestational age to have long-term speech problems
- Long-term learning difficulties may be more likely in infants born small for gestational age in comparison to infants born with birthweights appropriate for gestational age
- Those born large for gestational age may be more likely than infants with birthweights appropriate for gestational age to have long-term movement problems

How this study might affect research, practice, or policy

- This study has found possible adverse neurodevelopmental outcomes in specific subgroups of infants because of their birthweight, identifying a need for further research to better understand modifiable predictors of birthweight
- This also highlights a potential need for postnatal follow up of targeted groups and serial developmental assessments for early identification of developmental problems

For peer review only

INTRODUCTION

Background

Birthweight is a complex product of intrinsic and extrinsic influences on the foetal-maternal interaction and is an important predictor of perinatal morbidity and mortality.[1–3] Population studies have shown trends that infants are being born heavier. In England and Wales, there was an average increase of 40 grams over 26 years for all live births recorded between 1986 and 2012 with a 8-10% increased risk of being born with a high birthweight during this period.[4] Trends for heavier birthweights were also observed in other countries including Canada, USA and Sweden, and the cause for this shift is not understood.[4] This drives a need to better understand predictors of birthweight and the association with longer-term outcomes.

Short- and long-term outcomes for certain subgroups such as prematurity and small for gestational age (SGA) have been well explored but remains unclear for large for gestational age (LGA) infants. LGA is associated with birth complications and indirect effects persisting into adulthood.[5] Around birth, larger term infants have increased risk of shoulder dystocia, meconium aspiration, lower 5-minute Apgar scores and death.[6,7] Postnatally, they are more likely to have polycythemia, hypoglycaemia and respiratory distress syndrome; all conditions associated with poor long-term outcomes.[6–8] In preterm births, the prognosis for being LGA is unclear, with some literature suggesting an advantage; reporting lower perinatal mortality in preterm LGA infants, but with higher risks of early-onset sepsis and intraventricular haemorrhage.[9]

Overall, data on the long-term effects of being LGA across all gestational ages is lacking, and little is known about neurodevelopmental outcomes. A retrospective cohort study by Moore et.al found an increased risk of autism in term infants born SGA between 23 and 31 weeks, whereas being born large may have conferred some protective effect.[10] In view of speech being a dynamic product of higher function cognitive and sensorimotor feedback processes involving multiple cortical and subcortical

For peer review only

areas for planning, selecting, sequencing and motor programming, it was selected as a surrogate marker reflecting the child's ability to perform complex neurological tasks for this study.[11]

OBJECTIVES

To identify if infants born small, or large for gestational age, have more long-term speech problems as a marker of complex neurocognitive function than infants born with birthweights appropriate for gestational age.

METHODS

Study design

This study was conducted using data collected from the Respiratory and Neurological Outcomes of Children Born Preterm study (RANOPs), a cross-sectional population study conducted in Wales in 2013. This study recruited equal numbers of preterm (n=13361) and term-born children (n=13361) in years 2003, 2005, 2007, 2009 and 2011 to complete questionnaires on respiratory and neurodevelopmental outcomes. Term infants were selected to be comparable with the preterm infants, for date of birth, sex, and locality. A total of 7149 responses were received including consent for data usage and access to health databases. Parent-reported single-answer outcomes recorded as yes, no, or unsure, with a free-text response for additional information of reported problems were collected in 2013 for all ages. Baseline demographics including birth and maternal characteristics were collected from national health databases.[12–14]

The primary neurodevelopmental outcome was parent-reported speech problems, chosen as an indicator of higher function.[15] Secondary neurodevelopmental outcomes were learning, behavioural, hearing, movement, and hand problems. For this study, all 'unsure' responses were re-coded as 'no' and included in the primary analysis.

For peer review only

Study Population

The eligible population were all children enrolled in RANOPs, born from 23 weeks of gestation to mothers from 18 to 50 years of age with available speech outcomes (n=7004). Exposure measures were gestational age at birth and birthweight category. Birthweight centiles were calculated for each sex and gestation (in weeks) using the LMS Growth program (Medical Research Council).[16] SGA was defined as <10th centile on the UK-WHO growth charts and >90th centile for LGA. These are values generally accepted across England and Wales, with Scotland utilising the 5th and 95th centiles.[17,18] Gestational age was categorised (as per WHO definitions) as extremely preterm (<28 weeks), very preterm (28 to 31 weeks) and moderate to late preterm (32 to <37 weeks).[19] Post-maturity was defined as gestational age at birth greater than or equal to 42 weeks.[20] SGA and LGA infants were compared to appropriate for gestational age (AGA) infants across all gestations for analysis.

Covariates

Covariates included neonatal and maternal influences known a *priori* to influence birthweight in-utero. Neonatal factors are singleton or multiple births, sex, and mode of delivery. Maternal factors accounted for include smoking during pregnancy and documented Welsh Index of Multiple Deprivation (WIMD) score (1 being the most deprived) and age at the time of birth.[21]

Predictors of low birthweight are well studied but determinants of high birthweight are unclear. The most important risk factor for low birthweight is gestational age.[22] Multiple pregnancies are known to influence gestational age at birth and consequently, birthweight.[23] A cohort study of 2,078,327 singletons with no congenital anomalies born between 1999 and 2010 in the Netherlands showed that male infants have significantly higher average birthweights than females but were not more or less likely to be SGA or LGA.[24] Mode of delivery was likely multifactorial due to perinatal clinical practice surrounding estimated birthweight or centile, birth complications, and maternal preference.[17,25,26] For this study, mode of delivery was not assumed to impact birthweight.

For peer review only - http://bmjopen.bmj.com/site/about/guidelines.xhtml

Evidence suggests that active smoking is associated with increased risk of low birth weight and preterm birth, and prevalence of smoking is associated with socioeconomic status. [27,28] There is also an increased risk of low birthweight in the lower end of maternal age, influenced by socioeconomic status. Evidence surrounding birthweight and advanced maternal age is inconsistent with some studies showing increased prevalence of infants born LGA while others showing a U-shaped trend of low birthweight with increasing maternal age.[27,29–32]

Statistical Analysis

Duplicates and infants with missing exposure or primary outcome data were removed. Initially, neonatal, and maternal characteristics at birth for all infants were compared across their birthweight category. Comparisons were performed using the Kruskal-Wallis equality of populations ranks test for birthweight category, gestational age and WIMD score at birth. Sex, number of births, mode of delivery and maternal smoking during pregnancy were compared using the Chi-Square test, and maternal age at delivery using the Analysis of Variance (ANOVA) test.

Next, the proportion of children with speech problems between birthweight categories was compared. Using a logistic regression model, the unadjusted and adjusted for potential confounders, odds ratios for speech problems, comparing SGA and LGA infants to those born AGA were derived. Stratifying by preterm and term infants and using a logistic regression model, the odds ratios for speech problems were also derived for SGA and LGA infants compared to AGA infants. In view of a single primary outcome, p-values corresponding to each statistical test was not corrected.

Three sensitivity analyses were performed. The logistic regression model was repeated using a random-effects model clustered by year of birth, then using the 5th and 95th centile cut-offs and lastly,

For peer review only - http://bmjopen.bmj.com/site/about/guidelines.xhtml

removing all responders where 'unsure' was coded for the primary outcome.[18] Likelihood ratio tests were used to compare models. Analysis was performed using Stata SE 17 (Statacorp LLC).

Patient and Public Involvement

Patients or the public were not involved in the design, conduct, reporting or dissemination plans of this research.

RESULTS

4284 preterm and 2865 term infants were enrolled in RANOPs (n=7149).[13] 20 duplicates were removed, leaving 7129 children. Those born to mothers younger than 18 or above 50 years of age were excluded (n=83), leaving 7046 eligible children. 8 infants had missing birthweight centiles, and a further 34 had missing data on the primary outcome, long-term speech problems; leaving a study population of 7004 participants for the primary analysis (99.40% of the eligible population).

Stratifying by birthweight categories of SGA, AGA and LGA infants, baseline neonatal and maternal characteristics of the study cohort are shown in Table 1. The median age at the time of the survey was 4.08 to 5.17 years (p=0.69). There was no significant difference in the distribution of sex across birthweight categories (p=0.26). There were significant differences (p<0.001) in the distribution of all other baseline neonatal characteristics analysed. Mean maternal age (p<0.001), median WIMD score at the time of birth (p<0.001) and maternal smoking during pregnancy (p<0.001) were also significantly different between birthweight categories. (Table 1)

Table 1. Neonatal and maternal birth characteristics for all including preterm and term-born infants.

Characteristics		Birthweight centile			
	n	<10th	10-90th	>90th	P value
Age at survey (median years)	7003	4.08 (2.67-7.67)	5.17 (2.75-7.67)	4.37 (2.75-7.67)	0.69
Neonatal Factors					
Male	7004	313 (48.68%)	2458 (45.50%)	450 (46.92%)	0.26
Singleton	7004	507 (78.85%)	4647 (86.02%)	904 (94.26%)	<0.001
Gestation (median weeks)	7004	35 (33-38)	36 (34-39)	36 (34-38)	<0.001

For peer review only - http://bmjopen.bmj.com/site/about/guidelines.xhtml

Preterm (< 37 weeks)	7004	458 (71.23%)	3105 (57.48%)	623 (64.96%)	<0.001
Post term (≥ 42 weeks)	7004	25 (3.89%)	99 (1.83%)	5 (0.52%)	<0.001
Mode of delivery	4091				<0.001
Unassisted vaginal delivery	2037	119 (32.16%)	1659 (52.33%)	259 (47.01%)	
Instrumental	406	27 (7.30%)	334 (10.54%)	45 (8.17%)	
Elective caesarean section	511	66 (17.84%)	349 (11.01%)	96 (17.42%)	
Emergency caesarean section	1137	158 (42.70%)	828 (26.12%)	151 (27.40%)	
Maternal Factors					
Age (mean years)*	6225	29.85 (6.00)	30.35 (5.66)	31.15 (5.37)	<0.001
WIMD (median) †	6804	845 (428-1351)	985 (503-1447)	985 (524-1444)	<0.001
Maternal smoking‡	6725	133 (21.38%)	655 (12.65%)	75 (8.11%)	<0.001

Values are n (%), mean (SD) or median (IQR) as appropriate
Denominator between measures differs due to missing data
*Maternal age at the time of delivery
†Welsh Index of Multiple Deprivation (1 being the most deprived)
‡Maternal smoking during pregnancy

Distribution of long-term speech problems in preterm and term infants by birthweight centile is as shown in Figure 1. Analysis of the distribution of speech problems showed a significant difference between different birthweight categories (p=0.05). There was a significant difference (p<0.001) in the distribution of learning difficulties with 12.95% of SGA infants compared to 7.50% and 7.34% of AGA and LGA infants respectively. In the other secondary outcomes, the distribution of behavioural and hand problems varied across different birthweight categories whereas there were more infants born SGA and LGA with movement problems compared to AGA infants. Hypothesis testing showed a significant difference in the distribution of behavioural problems (p<0.001), movement problems (p=0.01) and hand problems (p=0.01) across birthweight categories but not in hearing problems (p=0.59). (Table 2)

Table 2. Long-term neurodevelopmental outcomes across all birthweight categories.

**Outcome Measure**	**n**	**Birthweight centile**			
		**< 10th n (%)**	**10-90th n (%)**	**> 90th n (%)**	**P value**
Primary					
Speech problems	7004	93 (14.46)	603 (11.16)	112 (11.68)	0.05
Secondary					
Learning difficulties	6980	83 (12.95)	404 (7.50)	70 (7.34)	<0.001
Behavioural problems	6963	86 (13.46)	459 (8.54)	77 (8.11)	<0.001
Hearing problems	6985	49 (7.66)	371 (6.89)	73 (7.62)	0.59
Movement problems	6990	45 (7.01)	240 (4.45)	49 (5.12)	0.01
Hand problems	7000	40 (6.22)	206 (3.82)	36 (3.76)	0.01

For peer review only

For peer review only - http://bmjopen.bmj.com/site/about/guidelines.xhtml

Logistic regression adjusted for neonatal and maternal features of the primary outcome long-term speech problems showed that LGA infants were not more likely than their AGA counterparts to have long-term speech problems, OR 0.95 (0.69-1.29). Infants born SGA were also not more likely than AGA infants to have speech problems, OR 1.28 (0.91-1.80). Analysis of secondary outcomes found evidence that SGA infants have an increased risk of learning difficulties compared to those born AGA, OR 1.73 (1.17-2.57). There was also some evidence that LGA infants are more likely to have movement problems, OR 1.83 (1.20-2.77). This study did not find that infants born SGA or LGA were more likely to have behavioural problems, hearing problems or hand problems in comparison. (Table 3)

Table 3. Unadjusted and adjusted odds ratios for long-term neurodevelopmental outcomes in SGA and LGA infants compared to AGA controls.

**Outcome Measures**	**Unadjusted**			**Adjusted for neonatal features***			**Adjusted for neonatal* and maternal features†**		
	n	Birthweight centile		n	Birthweight centile		n	Birthweight centile	
		< 10th	> 90th		< 10th	> 90th		< 10th	> 90th
Primary									
Speech problems	7004	1.34 (1.06-1.70)	1.05 (0.85-1.30)	4091	1.30 (0.94-1.79)	0.89 (0.67-1.20)	3565	1.28 (0.91-1.80)	0.95 (0.69-1.29)
Secondary									
Learning difficulties	6980	1.83 (1.42-2.36)	0.98 (0.75-1.27)	4078	1.82 (1.27-2.62)	1.13 (0.80-1.60)	3554	1.73 (1.17-2.57)	1.25 (0.87-1.81)
Behavioural problems	6963	1.66 (1.30-2.13)	0.94 (0.73-1.22)	4064	1.48 (1.03-2.12)	0.98 (0.69-1.39)	3542	1.27 (0.85-1.90)	1.22 (0.85-1.76)
Hearing problems	6985	1.12 (0.82-1.53)	1.11 (0.86-1.45)	4076	0.84 (0.52-1.35)	0.84 (0.57-1.25)	3554	0.88 (0.53-1.47)	0.88 (0.58-1.34)
Movement problems	6990	1.62 (1.16-2.25)	1.16 (0.84-1.59)	4087	1.43 (0.90-2.29)	1.56 (1.05-2.31)	3561	1.38 (0.82-2.33)	1.83 (1.20-2.77)
Hand problems	7000	1.67 (1.18-2.37)	0.98 (0.69-1.41)	4088	1.29 (0.78-2.13)	1.00 (0.63-1.59)	3562	1.11 (0.63-1.97)	1.14 (0.70-1.86)

*Adjusted for foetal sex, singleton or multiple birth, mode of delivery
†Adjusted for foetal sex, singleton or multiple birth, mode of delivery, maternal age and WIMD score at time of birth, and maternal smoking status during pregnancy

Analysis was repeated using a random-effects regression model stratified by preterm- or term-born and the adjusted odds ratios did not demonstrate that preterm infants born SGA, OR 1.38 (0.94-2.03) or LGA, OR 0.97 (0.68-1.38) were more likely to have long-term speech problems than AGA infants.

For peer review only

For peer review only - http://bmjopen.bmj.com/site/about/guidelines.xhtml

Similar results were also demonstrated with term-born SGA and LGA infants, OR 1.01 (0.49-2.09) and OR 0.90 (0.48-1.70). There was also no evidence of interaction between the exposures measured, ($p_{interaction}$=0.75). (Table 4)

Table 4. Unadjusted and adjusted odds ratios for primary neurodevelopmental outcome of long-term speech problems stratified by preterm or term in SGA and LGA infants compared to AGA controls.

Gestation	Birthweight category	Unadjusted (n = 7004)		Adjusted for neonatal features* (n = 4091)		Adjusted for neonatal* and maternal features† (n = 3582)	
		OR (95% CI)	$P_{interaction}$	OR (95% CI)	$P_{interaction}$	OR (95% CI)	$P_{interaction}$
**Preterm (<37 weeks)**	SGA	1.27 (0.98-1.66)	0.86	1.39 (0.97-1.99)	0.68	1.38 (0.94-2.03)	0.75
	LGA	1.04 (0.81-1.33)		0.93 (0.66-1.29)		0.97 (0.68-1.38)	
**Term (≥37 weeks)**	SGA	1.16 (0.69-1.96)		1.01 (0.49-2.07)		1.01 (0.49-2.09)	
	LGA	0.92 (0.60-1.42)		0.80 (0.43-1.49)		0.90 (0.48-1.70)	

*Adjusted for foetal sex, singleton or multiple birth, mode of delivery
†Adjusted for maternal age and WIMD score at time of birth, and maternal smoking status during pregnancy

Multi-level regression model of SGA and LGA infants compared to those born AGA clustered by year of birth showed no evidence of long-term speech problems in infants born SGA (OR 1.28 (0.91-1.80)) or LGA (OR 0.95 (0.69-1.29)). A repeat of the analysis using the 5th and 95th centiles as cut-offs for SGA and LGA did not show that SGA infants were more likely than AGA infants to have long-term speech problems, OR 1.47 (0.97-2.21). There was also no evidence to suggest similar in infants born LGA, OR 0.80 (0.53-1.19). When 'unsure' responses were excluded from analysis, logistic regression showed no increased likelihood in long-term speech problems in infants born SGA, OR 1.30 (0.92-1.83) or LGA, OR 0.97 (0.71-1.32)) compared to those born AGA.

DISCUSSION

The production of speech is complex, relying on not only intact cognitive, motor and sensory functions and can be complicated by heterogeneity of hearing loss, particularly the child's age at time of hearing loss. [11,33] Reassuringly, in this work we were unable to identify clear associations between SGA, or LGA

For peer review only - http://bmjopen.bmj.com/site/about/guidelines.xhtml

infants and adverse long-term speech outcomes; in either the unadjusted or adjusted models as well as in the analysis of hearing problems.

Evidence from this study suggest that SGA infants may have higher risks of long-term learning difficulties but not behavioural, hearing, movement or hand problems. A single-centre cohort study of term infants in Australia born to women presenting antenatally between 1981 and 1984 showed that children born SGA had significantly more learning difficulties when followed up at 14 years of age using a parent-reported survey and academic achievement test. This study reported a comparable 19.8% and 20.5% incidence for those who completed psychometric testing and behavioural questionnaires respectively. This study also demonstrated long-term attention difficulties in extremely SGA (3rd centile and below) term-born female adolescents.[34] Similar findings of poorer attention, executive function and memory were reported in a small cohort study of SGA preterm- and term-born young adults who underwent neuropsychological assessment.[35]

There was also evidence suggesting that LGA infants have a small increased risk of long-term movement problems. The potential increased risk in LGA infants could be influenced by neonatal baseline comorbidities such as poor Apgar scores at birth, shoulder dystocia and hypoxic-ischaemic encephalopathy; all diseases seen more comonly in larger infants. These comorbidities were unavailable for this study population and could be investigated in subsequent studies to identify possible causal relationships that might underpin this association. However long-term hand problems were not evident in this subgroup, as expected with the increased risk of shoulder dystocia in LGA infants and so interpretation of this association should be done cautiously.[6] Indeed, this study also did not find an increased risk of adverse long-term neurodevelopmental outcomes in speech, learning, behavioural and hearing domains in infants born LGA; although confidence intervals are relatively wide and important increases in morbidity cannot be excluded and work with more precise estimate may be warranted.

For peer review only - http://bmjopen.bmj.com/site/about/guidelines.xhtml

Analysis of baseline characteristics noted a significant difference in maternal age, and demographics at birth between birthweight categories. While this study was not designed to explore the relationship between maternal age and birthweight, previous studies have reported significant findings of higher maternal age in LGA infants.[29–32] In addition, the association between maternal smoking, deprivation, and birthweight was sobering, with 20% of SGA infants exposed to in-utero smoking. In this work, more than 1 in 5 mothers smoked during pregnancy, identifying an important public health focus on a modifiable risk factor for low birth weight to address,[27] with even first trimester cessation of smoking having substantial benefits.[27,36,37]

There was also a significant difference in the mode of delivery across birthweight centile categories likely due to clinical practice managing high-risk pregnancies[17,25,26]. This may include singleton or multiple pregnancies, gestational age and estimated birthweight or centile at time of birth. Being male or female did not influence being SGA or LGA, compatible with findings of a large cohort study in Netherlands[24].

STRENGTHS AND LIMITATIONS

- Like most cohort studies, missing data in those enrolled, particularly in confounders, was present, and potentially using parent-reported questionnaires may have introduced some degree of recall bias.
- In addition, while this study has a relatively large sample size, this represents around 25% of the eligible population, and is based on data collected between 2003 and 2011; and intepretation should consider this.
- While the number of infants is relatively large for a targeted data collection, confidence intervals around point estimates are relatively wide, and a smaller, but still important increase, or decrease in outcomes cannot be excluded.

For peer review only

- Finally, while we had broad data on most subjects, alongside potential confounders, residual confounding is a limitation of intepretation in all work such as this. However, point estimates remain compatible across the adjusted analyses.

CONCLUSIONS

In this work, infants born small or large for their gestation do not have higher risks of speech problems in childhood when compared to infants with appropriate birthweights. However important differences may still exist, and we found that some infants being born LGA may have increased movement problems, while those born SGA may have long-term learning difficulties; compared to appropriately grown infants

DECLARATIONS

Funding

No additional funding was received for this analysis. The original study, RANOPs was funded by Medical Research Council Experimental Medicine Challenge Grant (reg: MR/M022552/1), Mason Medical Research Foundation grant, and Children and Young Peoples Research Network Wales.

Competing interests

The authors have no relevant financial or non-financial interests to disclose.

Acknowledgements

We would like to thank all participants in this study.

Author contributions

For peer review only - http://bmjopen.bmj.com/site/about/guidelines.xhtml

Gabrielle Jee conceived and designed this work, wrote statistical analysis plan, cleaned, and analysed data, drafted first manuscript, revised draft manuscript and edited and updated final paper.

David Odd conceived and designed this work, wrote statistical analysis plan, analysed data and drafted first manuscript, revised draft manuscript and edited and updated final paper

Sarah J. Kotecha and Sailesh Kotecha conceived, designed, performed original RANOPs study including data collection, revised draft manuscript and edited and updated final paper.

Mallinath Chakraborty revised the draft manuscript and edited and updated final paper.

Ethical approval

Ethical approval was sought at initiation of RANOPs and approved by South East Wales Research Ethics Committee (Research Ethics Committee 12/WA/0155 Project 91349). Parents provided written consent to participate.

Data sharing

All data must be held securely at Cardiff University as per ethical approval given for this research. Anonymous data will be available from the department of Child Health at Cardiff University to bona fide researchers provided that ethical approval is obtained from a research ethics committee in the UK for any suggested studies. Requests for data access should be sent to Sailesh Kotecha (Kotechas@cardiff.ac.uk).

For peer review only

REFERENCES

1. Chiossi G, Pedroza C, Costantine MM, Truong VTT, Gargano G, Saade GR. Customized *vs* population-based growth charts to identify neonates at risk of adverse outcome: systematic review and Bayesian meta-analysis of observational studies. Ultrasound in Obstetrics & Gynecology. 2017 Aug;50(2).
2. Hutcheon JA, Zhang X, Platt RW, Cnattingius S, Kramer MS. The case against customised birthweight standards. Paediatric and Perinatal Epidemiology. 2011 Jan;25(1).
3. Baer RJ, Rogers EE, Partridge JC, Anderson JG, Morris M, Kuppermann M, et al. Population-based risks of mortality and preterm morbidity by gestational age and birth weight. Journal of Perinatology. 2016 Nov 28;36(11).
4. Ghosh RE, Berild JD, Sterrantino AF, Toledano MB, Hansell AL. Birth weight trends in England and Wales (1986–2012): babies are getting heavier. Archives of Disease in Childhood - Fetal and Neonatal Edition [Internet]. 2018 May;103(3):F264–70. Available from: https://fn.bmj.com/lookup/doi/10.1136/archdischild-2016-311790
5. Chiavaroli V, Derraik JGB, Hofman PL, Cutfield WS. Born Large for Gestational Age: Bigger Is Not Always Better. The Journal of Pediatrics. 2016 Mar;170:307–11.
6. Boulet SL, Alexander GR, Salihu HM, Pass M. Macrosomic births in the united states: Determinants, outcomes, and proposed grades of risk. American Journal of Obstetrics and Gynecology [Internet]. 2003 May;188(5):1372–8. Available from: https://linkinghub.elsevier.com/retrieve/pii/S0002937803001327
7. Khambalia AZ, Algert CS, Bowen JR, Collie RJ, Roberts CL. Long-term outcomes for large for gestational age infants born at term. Journal of Paediatrics and Child Health. 2017 Sep;53(9).
8. Dollberg S. Normoblasts in large for gestational age infants. Archives of Disease in Childhood - Fetal and Neonatal Edition. 2000 Sep 1;83(2).
9. Boghossian NS, Geraci M, Edwards EM, Horbar JD. In-Hospital Outcomes in Large for Gestational Age Infants at 22-29 Weeks of Gestation. The Journal of Pediatrics. 2018 Jul;198.
10. Moore GS, Kneitel AW, Walker CK, Gilbert WM, Xing G. Autism risk in small- and large-for-gestational-age infants. American Journal of Obstetrics and Gynecology [Internet]. 2012 Apr;206(4):314.e1-314.e9. Available from: https://linkinghub.elsevier.com/retrieve/pii/S0002937812001354
11. Tremblay P, Deschamps I, Gracco VL. Neurobiology of Speech Production. In: Neurobiology of Language. Elsevier; 2016. p. 741–50.
12. Edwards MO, Kotecha SJ, Lowe J, Richards L, Watkins WJ, Kotecha S. Early-term birth is a risk factor for wheezing in childhood: A cross-sectional population study. Journal of Allergy and Clinical Immunology [Internet]. 2015 Sep;136(3):581-587.e2. Available from: https://linkinghub.elsevier.com/retrieve/pii/S0091674915006971
13. Edwards MO, Kotecha SJ, Lowe J, Richards L, Watkins WJ, Kotecha S. Management of Prematurity-Associated Wheeze and Its Association with Atopy. PLOS ONE. 2016 May 20;11(5).
14. Lowe J, Kotecha SJ, Watkins WJ, Kotecha S. Effect of fetal and infant growth on respiratory symptoms in preterm-born children. Pediatric Pulmonology [Internet]. 2018 Feb;53(2):189–96. Available from: https://onlinelibrary.wiley.com/doi/10.1002/ppul.23920

For peer review only - http://bmjopen.bmj.com/site/about/guidelines.xhtml

15. Moini J, Piran P. Cerebral cortex. In: Functional and Clinical Neuroanatomy. Elsevier; 2020.
16. Pan H, Cole T. LMSgrowth, a Microsoft Excel add-in to access growth references based on the LMS method; Version 2.77 [Internet]. 2012. Available from: http://wwwhealthforallchildrencouk/
17. Royal College of Obstetricians & Gynaecologists. The Investigation and Management of the Small-for-Gestational-Age Fetus. Green-top Guideline No.31. 2nd Edition. 2014. p. 1–34.
18. Public Health Scotland. Appropriate Birthweight for Gestational Age [Internet]. 2020 [cited 2021 Oct 29]. Available from: https://www.opendata.nhs.scot/hu/dataset/births-in-scottish-hospitals/resource/a5d4de3f-e340-455f-b4e4-e26321d09207
19. World Health Organisation. Preterm Birth [Internet]. 2018 [cited 2019 Oct 29]. Available from: https://www.who.int/news-room/fact-sheets/detail/preterm-birth
20. The American College of Obstetricians and Gynecologists Committee on Obstetric Practice Society for Maternal-Fetal Medicine. Committee Opinion No 579: Definition of Term Pregnancy. Obstetrics & Gynecology. 2013 Nov;122(5).
21. Statistics for Wales. Welsh Index of Multiple Deprivation [Internet]. 2019 [cited 2022 Jan 31]. Available from: https://statswales.gov.wales/Catalogue/Community-Safety-and-Social-Inclusion/Welsh-Index-of-Multiple-Deprivation
22. Mohsin M, Wong F, Bauman A, Bai J. Maternal and Neonatal Factors Influencing Premature Birth and Low Birth Weight in Australia. Journal of Biosocial Science. 2003 Apr 20;35(2):161–74.
23. Gielen M, van Beijsterveldt CEM, Derom C, Vlietinck R, Nijhuis JG, Zeegers MPA, et al. Secular trends in gestational age and birthweight in twins. Human Reproduction. 2010 Sep;25(9):2346–53.
24. Voskamp BJ, Peelen MJCS, Ravelli ACJ, Lee R, Mol BWJ, Pajkrt E, et al. Association between fetal sex, birthweight percentile and adverse pregnancy outcome. Acta Obstetricia et Gynecologica Scandinavica. 2020 Jan 30;99(1):48–58.
25. Royal College of Obstetricians and Gynaecologists. Shoulder Dystocia [Internet]. Green-top Guideline No.42, 2nd Edition. 2012 [cited 2022 Jan 11]. p. 1–18. Available from: https://www.rcog.org.uk/globalassets/documents/guidelines/gtg_42.pdf
26. Management of Breech Presentation. BJOG: An International Journal of Obstetrics & Gynaecology. 2017 Jun;124(7):e151–77.
27. Johnson C, Jones S, Paranjothy S. Low Birth Weight Review. 2014 Jul 4 [cited 2022 Jan 31]; Available from: https://www2.nphs.wales.nhs.uk/ChildrenMatFamiliesDocs.nsf/($all)/E3F761EC6EFE646F80257D490044FBAE/$file/Low%20Birth%20Weight%20-%20technical%20paper%20v1.pdf?OpenElement
28. McAndrew F, Thompson J, Fellows L, Large A, Speed M, Renfrew M. Infant Feeding Survey 2010 [Internet]. 2010 [cited 2022 Jan 31]. Available from: https://sp.ukdataservice.ac.uk/doc/7281/mrdoc/pdf/7281_ifs-uk-2010_report.pdf
29. Weissmann-Brenner A, Simchen M, Zilberberg E, Kalter A, Weisz B, Achiron R, et al. Maternal and neonatal outcomes of large for gestational age pregnancies. Acta Obstetricia et Gynecologica Scandinavica. 2012 Jul;91(7):844–9.

For peer review only - http://bmjopen.bmj.com/site/about/guidelines.xhtml

30. Siggelkow W, Boehm D, Skala C, Grosslercher M, Schmidt M, Koelbl H. The influence of macrosomia on the duration of labor, the mode of delivery and intrapartum complications. Archives of Gynecology and Obstetrics. 2008 Dec 1;278(6):547–53.
31. Akin Y, Cömert S, Turan C, Piçak A, Ağzikuru T, Telatar B. Macrosomic newborns: a 3-year review. Turk J Pediatr. 52(4):378–83.
32. Bakker R, Steegers E, Biharie A, Mackenbach J, Hofman A, Jaddoe V. Explaining differences in birth outcomes in relation to maternal age: the Generation R Study. BJOG: An International Journal of Obstetrics & Gynaecology. 2011 Mar;118(4):500–9.
33. Dobie R, van Hemel S. Hearing Loss: Determining Eligibility for Social Security Benefits [Internet]. Committee on Disability Determination for Individuals with Hearing Impairments, National Research Council, editors. Washington (DC); 2004 [cited 2022 May 26]. 180–223 p. Available from: https://www.ncbi.nlm.nih.gov/books/NBK207837/
34. O'Keeffe MJ, O'Callaghan M, Williams GM, Najman JM, Bor W. Learning, Cognitive, and Attentional Problems in Adolescents Born Small for Gestational Age. Pediatrics [Internet]. 2003 Aug 1;112(2):301–7. Available from: https://publications.aap.org/pediatrics/article/112/2/301/63295/Learning-Cognitive-and-Attentional-Problems-in
35. Østgård HF, Skranes J, Martinussen M, Jacobsen GW, Brubakk AM, Vik T, et al. Neuropsychological Deficits in Young Adults Born Small-for-Gestational Age (SGA) at Term. Journal of the International Neuropsychological Society. 2014 Mar 24;20(3):313–23.
36. Jaddoe VW v., Troe EJWM, Hofman A, Mackenbach JP, Moll HA, Steegers EAP, et al. Active and passive maternal smoking during pregnancy and the risks of low birthweight and preterm birth: the Generation R Study. Paediatric and Perinatal Epidemiology. 2008 Mar;22(2):162–71.
37. Raatikainen K, Huurinainen P, Heinonen S. Smoking in early gestation or through pregnancy: A decision crucial to pregnancy outcome. Preventive Medicine. 2007 Jan;44(1):59–63.

For peer review only

Figure 1. Proportion of preterm, and term infants with speech problems by birthweight centile (Gaussian smoothed).

For peer review only

For peer review only - http://bmjopen.bmj.com/site/about/guidelines.xhtml

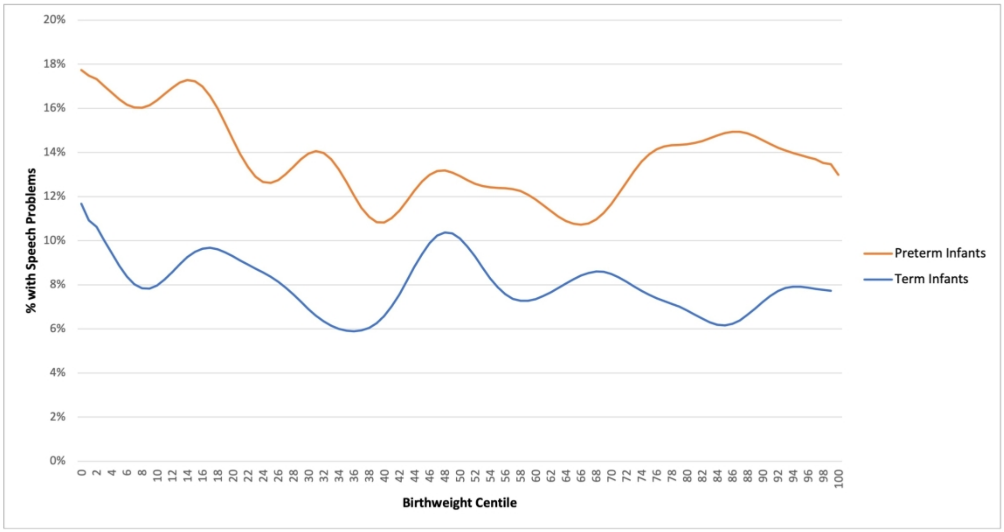

Figure 1. Proportion of term, and preterm infants with speech problems by birthweight centile (Gaussian smoothed).

247x130mm (300 x 300 DPI)

STROBE Statement—Checklist of items that should be included in reports of ***cross-sectional studies***

	Item No	Recommendation	Page No
**Title and abstract**	1	(*a*) Indicate the study's design with a commonly used term in the title or the abstract	1
		(*b*) Provide in the abstract an informative and balanced summary of what was done and what was found	2 - 3
**Introduction**			
Background/rationale	2	Explain the scientific background and rationale for the investigation being reported	5
Objectives	3	State specific objectives, including any prespecified hypotheses	6
**Methods**			
Study design	4	Present key elements of study design early in the paper	6
Setting	5	Describe the setting, locations, and relevant dates, including periods of recruitment, exposure, follow-up, and data collection	6
Participants	6	(*a*) Give the eligibility criteria, and the sources and methods of selection of participants	7
Variables	7	Clearly define all outcomes, exposures, predictors, potential confounders, and effect modifiers. Give diagnostic criteria, if applicable	7-8
Data sources/ measurement	8*	For each variable of interest, give sources of data and details of methods of assessment (measurement). Describe comparability of assessment methods if there is more than one group	7-8
Bias	9	Describe any efforts to address potential sources of bias	7-8
Study size	10	Explain how the study size was arrived at	6-7
Quantitative variables	11	Explain how quantitative variables were handled in the analyses. If applicable, describe which groupings were chosen and why	7-8
Statistical methods	12	(*a*) Describe all statistical methods, including those used to control for confounding	8-9
		(*b*) Describe any methods used to examine subgroups and interactions	8-9
		(*c*) Explain how missing data were addressed	8-9
		(*d*) If applicable, describe analytical methods taking account of sampling strategy	N/A
		(*e*) Describe any sensitivity analyses	8-9
**Results**			
Participants	13*	(a) Report numbers of individuals at each stage of study—eg numbers potentially eligible, examined for eligibility, confirmed eligible, included in the study, completing follow-up, and analysed	9-11
		(b) Give reasons for non-participation at each stage	N/A
		(c) Consider use of a flow diagram	N/A
Descriptive data	14*	(a) Give characteristics of study participants (eg demographic, clinical, social) and information on exposures and potential confounders	9-11
		(b) Indicate number of participants with missing data for each variable of interest	9-11
Outcome data	15*	Report numbers of outcome events or summary measures	9-11
Main results	16	(*a*) Give unadjusted estimates and, if applicable, confounder-adjusted estimates and their precision (eg, 95% confidence interval). Make clear which confounders were adjusted for and why they were included	9-11

For peer review only

		(*b*) Report category boundaries when continuous variables were categorized	9-11
		(*c*) If relevant, consider translating estimates of relative risk into absolute risk for a meaningful time period	N/A
Other analyses	17	Report other analyses done—eg analyses of subgroups and interactions, and sensitivity analyses	9-11
**Discussion**			
Key results	18	Summarise key results with reference to study objectives	11-12
Limitations	19	Discuss limitations of the study, taking into account sources of potential bias or imprecision. Discuss both direction and magnitude of any potential bias	12-13
Interpretation	20	Give a cautious overall interpretation of results considering objectives, limitations, multiplicity of analyses, results from similar studies, and other relevant evidence	13
Generalisability	21	Discuss the generalisability (external validity) of the study results	11-12
**Other information**			
Funding	22	Give the source of funding and the role of the funders for the present study and, if applicable, for the original study on which the present article is based	13

*Give information separately for exposed and unexposed groups.

Note: An Explanation and Elaboration article discusses each checklist item and gives methodological background and published examples of transparent reporting. The STROBE checklist is best used in conjunction with this article (freely available on the Web sites of PLoS Medicine at http://www.plosmedicine.org/, Annals of Internal Medicine at http://www.annals.org/, and Epidemiology at http://www.epidem.com/). Information on the STROBE Initiative is available at www.strobe-statement.org.

For peer review only

BMJ Open

Early childhood parent-reported speech problems in small and large for gestational age term- and preterm-born infants: A cohort study

Journal:	*BMJ Open*
Manuscript ID	bmjopen-2022-065587.R1
Article Type:	Original research
Date Submitted by the Author:	27-Dec-2022
Complete List of Authors:	Jee, Gabrielle; University of Wales Hospital, Paediatrics<br>Kotecha, Sarah; Cardiff University School of Medicine, Department of Child Health<br>Chakraborty, Mallinath; Cardiff University School of Medicine, Department of Child Health; University of Wales Hospital, Department of Neonatology<br>Kotecha, Sailesh; Cardiff University School of Medicine, Department of Child Health; University of Wales Hospital, Department of Neonatology<br>Odd, David; University Hospital of Wales, Department of Neonatology; Cardiff University School of Medicine, Department of Population Health
**Primary Subject Heading**:	Paediatrics
Secondary Subject Heading:	Neurology
Keywords:	NEONATOLOGY, Developmental neurology & neurodisability < PAEDIATRICS, Neonatal intensive & critical care < INTENSIVE & CRITICAL CARE

SCHOLARONE™
Manuscripts

I, the Submitting Author has the right to grant and does grant on behalf of all authors of the Work (as defined in the below author licence), an exclusive licence and/or a non-exclusive licence for contributions from authors who are: i) UK Crown employees; ii) where BMJ has agreed a CC-BY licence shall apply, and/or iii) in accordance with the terms applicable for US Federal Government officers or employees acting as part of their official duties; on a worldwide, perpetual, irrevocable, royalty-free basis to BMJ Publishing Group Ltd ("BMJ") its licensees and where the relevant Journal is co-owned by BMJ to the co-owners of the Journal, to publish the Work in this journal and any other BMJ products and to exploit all rights, as set out in our licence.

The Submitting Author accepts and understands that any supply made under these terms is made by BMJ to the Submitting Author unless you are acting as an employee on behalf of your employer or a postgraduate student of an affiliated institution which is paying any applicable article publishing charge ("APC") for Open Access articles. Where the Submitting Author wishes to make the Work available on an Open Access basis (and intends to pay the relevant APC), the terms of reuse of such Open Access shall be governed by a Creative Commons licence – details of these licences and which Creative Commons licence will apply to this Work are set out in our licence referred to above.

Other than as permitted in any relevant BMJ Author's Self Archiving Policies, I confirm this Work has not been accepted for publication elsewhere, is not being considered for publication elsewhere and does not duplicate material already published. I confirm all authors consent to publication of this Work and authorise the granting of this licence.

For peer review only

Early childhood parent-reported speech problems in small and large for gestational age term- and preterm-born infants: A cohort study

Jee, Gabrielle[a], Kotecha, Sarah J.[b], Chakraborty, Mallinath [b,c], Kotecha, Sailesh[b,c], Odd, David[c,d]

[a]*Department of Paediatrics, University Hospital of Wales, Cardiff, UK*

[b]*Department of Child Health, School of Medicine, Cardiff University, Cardiff, UK*

[c]*Department of Neonatology, University Hospital of Wales, Cardiff, UK*

[d]*Department of Population Health, School of Medicine, Cardiff University, Cardiff, UK*

Corresponding author:

David Odd

Neuadd Meirionnydd, University Hospital of Wales, Heath Park, Cardiff, CF14 4YS

oddd@cardiff.ac.uk

+44(0)2920874000

Keywords:

Neonates, birthweight, neurodevelopmental outcome

Word count:

3564 words

For peer review only - http://bmjopen.bmj.com/site/about/guidelines.xhtml

For peer review only

ABSTRACT

Objective

1. To assess if preterm and term small (SGA) or large for gestational age (LGA) infants have more parent-reported speech problems in early childhood compared to infants with birthweights appropriate for gestational age (AGA).
2. To assess if preterm and term SGA and LGA infants have more learning, behavioural, hearing, movement, and hand problems compared to AGA infants.

Design

Cohort study.

Setting

Wales, United Kingdom.

Participants

7004 children with neurodevelopmental outcomes from the Respiratory and Neurological Outcomes of Children Born Preterm study (RANOPs) which recruited 7129 responders, born from 23 weeks of gestation onwards to mothers aged 18 to 50 years of age were eligible.

Outcome Measures

Parent-reported single-answer questionnaires were completed in 2013 to assess early childhood neurodevelopmental outcomes . The primary outcome was parent-reported speech problems adjusted for clinical and demographic confounders in SGA and LGA

For peer review only

infants compared to AGA infants. Secondary outcomes measured were learning, behavioural, hearing, movement, and hand problems.

Results

Median age at time of study was 5 years, range 2-10 years. Although the adjusted OR was 1.28 (0.91-1.80) for SGA infants and OR 0.95 (0.69-1.29) for LGA infants, this failed to reach statistical significance that these subgroups were more likely to have parent-reported speech problems in early childhood compared to AGA infants. This study also found parent-reported early childhood evidence suggesting potential learning difficulties in SGA infants (OR 1.73 (1.17-2.57)) and movement problems in LGA infants (OR 1.83 (1.20-2.77)).

Conclusion

Infants born SGA or LGA do not have higher risks of parent-reported speech problems in childhood compared to infants born AGA.

(250 words)

For peer review only - http://bmjopen.bmj.com/site/about/guidelines.xhtml

STRENGTHS AND LIMITATIONS

- Like most cohort studies, missing data in those enrolled, particularly in confounders, was present, and potentially using parent-reported questionnaires may have introduced some degree of recall bias
- The study has a relatively large sample size, although this represents around 25% of the possible eligible population. It is also based on data collected between 2003 and 2011; and intepretation should consider this.
- The number of infants is relatively large for a targeted data collection, however due to some outcomes being relatively rare, confidence intervals around point estimates are broad, and smaller, but still important differences in outcomes cannot be excluded.
- We had broad data on most subjects, including potential confounders, and while residual confounding is a limitation of observational work point estimates remain compatible across the unadjusted, adjusted and sensitivity analyses.

For peer review only

INTRODUCTION

Background

Birthweight is a complex product of intrinsic and extrinsic influences on the foetal-maternal interaction and is an important predictor of perinatal morbidity and mortality.[1]–[3] Population studies have shown trends that infants are being born heavier. In England and Wales, there was an average increase of 40 grams over 26 years for all live births recorded between 1986 and 2012 with a 8-10% increased risk of being born with a high birthweight during this period.[4] Trends for heavier birthweights were also observed in other countries including Canada, USA and Sweden, and the cause for this shift is not understood.[4] This drives a need to better understand predictors of birthweight and the association with longer-term outcomes.

Outcomes for certain subgroups such as prematurity and small for gestational age (SGA) have been well explored but remains unclear for large for gestational age (LGA) infants particularly mid- to long-term outcomes. LGA is associated with birth complications and indirect effects persisting into adulthood.[5] Around birth, larger term infants have increased risk of shoulder dystocia, meconium aspiration, lower 5-minute Apgar scores and death.[6], [7] Postnatally, they are more likely to have polycythemia, hypoglycaemia and respiratory distress syndrome; all conditions associated with poor long-term outcomes.[6]–[8] In preterm births, the prognosis for being LGA is unclear, with some literature suggesting an advantage; reporting lower perinatal mortality in preterm LGA infants, but with higher risks of early-onset sepsis and intraventricular haemorrhage.[9]

For peer review only - http://bmjopen.bmj.com/site/about/guidelines.xhtml

Overall, data on the mid- to long-term effects of being LGA across all gestational ages is lacking, and little is known about neurodevelopmental outcomes. A retrospective cohort study by Moore et.al found an increased risk of autism in term infants born SGA between 23 and 31 weeks, whereas being born large may have conferred some protective effect.[10] In view of speech being a dynamic product of higher function cognitive and sensorimotor feedback processes involving multiple cortical and subcortical areas for planning, selecting, sequencing and motor programming, it was selected as the primary outcome of interest in addition to relative ease of quantification by parents. Due to the complex interaction between neurodevelopmental domains incuding speech, this study also evaluated learning, behavioural, hearing, movement, and hand problems.[11]

OBJECTIVES

1. To assess if preterm and term small (SGA) or large for gestational age (LGA) infants have more parent-reported speech problems in early childhood compared to infants with birthweights appropriate for gestational age (AGA).
2. To assess if preterm and term SGA and LGA infants have more learning, behavioural, hearing, movement, and hand problems compared to AGA infants.

METHODS

Study design

This study was conducted using data collected from the Respiratory and Neurological Outcomes of Children Born Preterm study (RANOPs), a cross-sectional population study conducted in Wales in 2013. This study recruited equal numbers of preterm (n=13361) and term-born children (n=13361) in years 2003, 2005, 2007, 2009 and 2011 to complete questionnaires on respiratory and neurodevelopmental outcomes if

For peer review only - http://bmjopen.bmj.com/site/about/guidelines.xhtml

their child has ever had any active or resolved problems in their lifetime. [Supplement 1, 2]

Term infants were selected to be comparable with the preterm infants, for date of birth, sex, and locality. A total of 7149 responses were received including consent for data usage and access to health databases. Parent-reported single-answer outcomes recorded as yes, no, or unsure, with a free-text response for additional information of reported problems were collected in 2013 for all ages. For example, "Does your child have any problems with their speech? If yes, please provide some details" followed by a yes or no option, and free text space for the additional details. Baseline demographics including birth and maternal characteristics were collected from national health databases.[12]–[14]

The primary neurodevelopmental outcome was parent-reported speech problems.[11] Secondary neurodevelopmental outcomes were learning, behavioural, hearing, movement, and hand problems. For this study, all 'unsure' responses were re-coded as 'no' and included in the primary analysis.

Study Population

The eligible population were all children enrolled in RANOPs, born from 23 weeks of gestation to mothers from 18 to 50 years of age with available speech outcomes (n=7004). Exposure measures were gestational age at birth and birthweight centile by category. Birthweight centiles were calculated for each sex and gestation (in weeks) using the LMS Growth program (Medical Research Council).[15] SGA was defined as <10th centile on the UK-WHO growth charts and >90th centile for LGA. These are

For peer review only - http://bmjopen.bmj.com/site/about/guidelines.xhtml

values generally accepted across England and Wales, with Scotland utilising the 5th and 95th centiles.[16], [17] Gestational age was categorised (as per WHO definitions) as extremely preterm (<28 weeks), very preterm (28 to 31 weeks) and moderate to late preterm (32 to <37 weeks).[18] Post-maturity was defined as gestational age at birth greater than or equal to 42 weeks.[19] SGA and LGA infants were compared to appropriate for gestational age (AGA) infants across all gestations for analysis.

Covariates

Covariates included neonatal and maternal influences known a *priori* to influence birthweight in-utero. Neonatal factors are singleton or multiple births, sex, and mode of delivery. Maternal factors accounted for include smoking during pregnancy and documented Welsh Index of Multiple Deprivation (WIMD) score (1 being the most deprived) and age at the time of birth.[20] This overlaps with potential influences on the primary outcome, parent-reported speech problems in early childhood. While complex and multifactorial in potentially influencing the primary outcome, maternal socioeconomic status was accounted for using WIMD scores.[21] Predictors of low birthweight are well studied but determinants of high birthweight are unclear. The most important risk factor for low birthweight is gestational age.[22] Multiple pregnancies are known to influence gestational age at birth and consequently, birthweight.[23] A cohort study of 2,078,327 singletons with no congenital anomalies born between 1999 and 2010 in the Netherlands showed that male infants have significantly higher average birthweights than females but were not more or less likely to be SGA or LGA.[24] Mode of delivery was likely multifactorial due to perinatal clinical practice surrounding estimated birthweight or centile, birth complications, and maternal

For peer review only

preference.[16], [25], [26] For this study, mode of delivery was not assumed to impact birthweight.

Evidence suggests that active smoking is associated with increased risk of low birth weight and preterm birth, and prevalence of smoking is associated with socioeconomic status. [27], [28] There is also an increased risk of low birthweight in the lower end of maternal age, influenced by socioeconomic status. Evidence surrounding birthweight and advanced maternal age is inconsistent with some studies showing increased prevalence of infants born LGA while others showing a U-shaped trend of low birthweight with increasing maternal age.[27], [29]–[32]

Statistical Analysis

Duplicates and infants with missing exposure or primary outcome data were removed. Initially, neonatal, and maternal characteristics at birth for all infants were compared across their birthweight centile category. Comparisons were performed using the Kruskal-Wallis equality of populations ranks test for birthweight centile category, gestational age and WIMD score at birth. Sex, number of births, mode of delivery and maternal smoking during pregnancy were compared using the Chi-Square test, and maternal age at delivery using the Analysis of Variance (ANOVA) test.

Next, the proportion of children with speech problems between birthweight centile categories was compared. Using a logistic regression model, the unadjusted and adjusted for potential confounders, odds ratios for speech problems, comparing SGA and LGA infants to those born AGA were derived. Stratifying by preterm and term infants and using a logistic regression model, the odds ratios for speech problems

For peer review only - http://bmjopen.bmj.com/site/about/guidelines.xhtml

were also derived for SGA and LGA infants compared to AGA infants. In view of a single primary outcome, p-values corresponding to each statistical test was not corrected.

Six sensitivity analyses were performed. Firstly, we repeated the logistic regression using a random-effects model to cluster by week of gestational age, and secondly by the age of the child at time of the survey. We then repeated using the 5th and 95th centile cut-offs and then again removing all responders where 'unsure' was coded for the primary outcome.[17] The main analysis was also repeated with an ordinal logistic regression analysis looking at the odds of an increasing number of reported neurodevelopmental disorders. Finally, the analysis was repeated using a missing data technique (Multiple Imputation with Chain Equations (details in Supplement 3) to assess the impact of missing outcome and covariate data on the association seen.[17] Likelihood ratio tests were used to compare models. Analysis was performed using Stata SE 17 (Statacorp LLC).

Patient and Public Involvement

Patients or the public were not involved in the design, conduct, reporting or dissemination plans of this research.

RESULTS

4284 preterm and 2865 term infants were enrolled in RANOPs (n=7149).[13] 20 duplicates were removed, leaving 7129 children. Those born to mothers younger than 18 or above 50 years of age were excluded (n=83), leaving 7046 eligible children. 8 infants had missing birthweight centiles, and a further 34 had missing data on the

For peer review only - http://bmjopen.bmj.com/site/about/guidelines.xhtml

primary outcome, long-term speech problems; leaving a study population of 7004 participants for the primary analysis (99.40% of the eligible population).

Stratifying by birthweight centile categories of SGA, AGA and LGA infants, baseline neonatal and maternal characteristics of the study cohort are shown in Table 1. The median age at time of the survey was 4.08 to 5.17 (p=0.69). Besides the distribution of sex across birthweight centile categories, there were significant differences in other baseline neonatal and maternal characteristics analysed.

Table 1. Neonatal and maternal birth characteristics for all including preterm and term-born infants.

**Characteristics**		**Birthweight centile**			
	**n**	**SGA (<10th)**	**AGA (10-90th)**	**LGA (>90th)**	**P value**
Age at survey (median years)	7003	4.08 (2.67-7.67)	5.17 (2.75-7.67)	4.37 (2.75-7.67)	0.69
Neonatal Factors					
Male	7004	313 (48.68%)	2458 (45.50%)	450 (46.92%)	0.26
Singleton	7004	507 (78.85%)	4647 (86.02%)	904 (94.26%)	<0.001
Gestation (median weeks)	7004	35 (33-38)	36 (34-39)	36 (34-38)	<0.001
Preterm (< 37 weeks)	7004	458 (71.23%)	3105 (57.48%)	623 (64.96%)	<0.001
Post term (≥ 42 weeks)	7004	25 (3.89%)	99 (1.83%)	5 (0.52%)	<0.001
Mode of delivery	4091				<0.001
Unassisted vaginal delivery	2037	119 (32.16%)	1659 (52.33%)	259 (47.01%)	
Instrumental	406	27 (7.30%)	334 (10.54%)	45 (8.17%)	
Elective caesarean section	511	66 (17.84%)	349 (11.01%)	96 (17.42%)	
Emergency caesarean section	1137	158 (42.70%)	828 (26.12%)	151 (27.40%)	
Maternal Factors					
Age (mean years)*	6225	29.85 (6.00)	30.35 (5.66)	31.15 (5.37)	<0.001
WIMD (median) †	6804	845 (428-1351)	985 (503-1447)	985 (524-1444)	<0.001
Maternal smoking‡	6725	133 (21.38%)	655 (12.65%)	75 (8.11%)	<0.001

Values are n (%), mean (SD) or median (IQR) as appropriate
Denominator between measures differs due to missing data
*Maternal age at the time of delivery
†Welsh Index of Multiple Deprivation (1 being the most deprived)
‡Maternal smoking during pregnancy

For peer review only - http://bmjopen.bmj.com/site/about/guidelines.xhtml

Distribution of long-term speech problems in preterm and term infants by birthweight centile is as shown in Figure 1. Figure 2 depicts the number of neurodevelopmental disorders stratified by gestation age at birth, birthweight centile category, and deprivation measures or WIMD category (quintile). Analysis of the distribution of the primary outcome, parent-reported speech problems showed a significant difference between different birthweight centile categories, p=0.05. For all other secondary outcomes, there were significant differences between birthweight centile categories except for in the distribution of parent-reported hearing problems (p=0.59) as per Table 2.

Table 2. Long-term neurodevelopmental outcomes across all birthweight categories.

**Outcome Measure**	**n**	**Birthweight centile**			
		**SGA ($<10^{th}$) n (%)**	**AGA ($10\text{-}90^{th}$) n (%)**	**LGA ($>90^{th}$) n (%)**	**P value**
Primary					
Speech problems	7004	93 (14.46)	603 (11.16)	112 (11.68)	0.05
Secondary					
Learning difficulties	6980	83 (12.95)	404 (7.50)	70 (7.34)	<0.001
Behavioural problems	6963	86 (13.46)	459 (8.54)	77 (8.11)	<0.001
Hearing problems	6985	49 (7.66)	371 (6.89)	73 (7.62)	0.59
Movement problems	6990	45 (7.01)	240 (4.45)	49 (5.12)	0.01
Hand problems	7000	40 (6.22)	206 (3.82)	36 (3.76)	0.01

Logistic regression adjusted for neonatal and maternal features of the primary outcome, parent-reported speech problems in early childhood showed that SGA and LGA infants were not more likely than their AGA counterparts to have problems, OR 1.28 (0.91-1.80) and OR 0.95 (0.69-1.29) respectively. Analysis of parent-reported secondary outcomes found evidence that SGA infants have an increased risk of learning difficulties compared to those born AGA, OR 1.73 (1.17-2.57). There was also some evidence that LGA infants are more likely to have movement problems, OR 1.83 (1.20-2.77). This study did not find that infants born SGA or LGA were more likely to have behavioural problems, hearing problems or hand problems in comparison to AGA infants. [Table 3]

For peer review only - http://bmjopen.bmj.com/site/about/guidelines.xhtml

Table 3. Unadjusted and adjusted odds ratios for long-term neurodevelopmental outcomes in SGA and LGA infants compared to AGA controls.

**Outcome Measures**	**Unadjusted**			**Adjusted for neonatal features***			**Adjusted for neonatal* and maternal features†**		
	n	Birthweight centile SGA (<10th)	Birthweight centile LGA (>90th)	n	Birthweight centile SGA (<10th)	Birthweight centile LGA (>90th)	n	Birthweight centile SGA (<10th)	Birthweight centile LGA (>90th)
Primary									
Speech problems	7004	1.34 (1.06-1.70)	1.05 (0.85-1.30)	4091	1.30 (0.94-1.79)	0.89 (0.67-1.20)	3565	1.28 (0.91-1.80)	0.95 (0.69-1.29)
Secondary									
Learning difficulties	6980	1.83 (1.42-2.36)	0.98 (0.75-1.27)	4078	1.82 (1.27-2.62)	1.13 (0.80-1.60)	3554	1.73 (1.17-2.57)	1.25 (0.87-1.81)
Behavioural problems	6963	1.66 (1.30-2.13)	0.94 (0.73-1.22)	4064	1.48 (1.03-2.12)	0.98 (0.69-1.39)	3542	1.27 (0.85-1.90)	1.22 (0.85-1.76)
Hearing problems	6985	1.12 (0.82-1.53)	1.11 (0.86-1.45)	4076	0.84 (0.52-1.35)	0.84 (0.57-1.25)	3554	0.88 (0.53-1.47)	0.88 (0.58-1.34)
Movement problems	6990	1.62 (1.16-2.25)	1.16 (0.84-1.59)	4087	1.43 (0.90-2.29)	1.56 (1.05-2.31)	3561	1.38 (0.82-2.33)	1.83 (1.20-2.77)
Hand problems	7000	1.67 (1.18-2.37)	0.98 (0.69-1.41)	4088	1.29 (0.78-2.13)	1.00 (0.63-1.59)	3562	1.11 (0.63-1.97)	1.14 (0.70-1.86)

*Adjusted for foetal sex, singleton or multiple birth, mode of delivery
†Adjusted for foetal sex, singleton or multiple birth, mode of delivery, maternal age and WIMD score at time of birth, and maternal smoking status during pregnancy

Analysis was repeated using a random-effects regression model stratified by preterm- or term-born and the adjusted odds ratios did not demonstrate that preterm infants born SGA or LGA were more likely to have parent-reported speech-problems than infants born AGA. There was also no evidence of interaction between the exposures measured, ($p_{interaction}$=0.75). [Table 4]

Table 4. Unadjusted and adjusted odds ratios for primary neurodevelopmental outcome of long-term speech problems stratified by preterm or term in SGA and LGA infants compared to AGA controls.

**Gestation**	**Birthweight category**	**Unadjusted (n = 7004)**		**Adjusted for neonatal features* (n = 4091)**		**Adjusted for neonatal* and maternal features† (n = 3582)**	
		OR (95% CI)	$P_{interaction}$	OR (95% CI)	$P_{interaction}$	OR (95% CI)	$P_{interaction}$
**Preterm (<37 weeks)**	SGA (<10th)	1.27 (0.98-1.66)	0.86	1.39 (0.97-1.99)	0.68	1.38 (0.94-2.03)	0.75

For peer review only - http://bmjopen.bmj.com/site/about/guidelines.xhtml

	LGA (>90th)	1.04 (0.81-1.33)		0.93 (0.66-1.29)		0.97 (0.68-1.38)	
**Term (≥37 weeks)**	SGA (<10th)	1.16 (0.69-1.96)		1.01 (0.49-2.07)		1.01 (0.49-2.09)	
	LGA (>90th)	0.92 (0.60-1.42)		0.80 (0.43-1.49)		0.90 (0.48-1.70)	

*Adjusted for foetal sex, singleton or multiple birth, mode of delivery
†Adjusted for maternal age and WIMD score at time of birth, and maternal smoking status during pregnancy

Multi-level regression model of SGA and LGA babies compared to those born AGA across each gestational age category using child age as the clustering variable also showed compatible results (SGA, OR 1.28 (0.91-1.81); LGA, OR 0.95 (0.69-1.28) with the main analysis. When 'unsure' responses excluded from analysis, logistic regression repeated showed no increased likelihood in primary neurodevelopmental outcome, parent-reported speech problems in babies born SGA, OR 1.30 (0.92 – 1.83) or LGA, OR 0.97 (0.71 – 1.32) compared to those born AGA. Repeat of the analysis using the 5th and 95th centiles as cut-offs for SGA showed that babies born SGA were more not likely babies born AGA to have parent-reported speech problems, OR 1.47 (0.97 – 2.21).[17] There was also no evidence to suggest similar in babies born LGA, OR 0.80 (0.53 – 1.19). Repeat of the main analysis looking at the odds of an increasing number of reported developmental disorders, results are compatible with the primary analysis (SGA, OR 1.15 (0.88-1.51); LGA, OR 1.08 (0.86-1.37). Finally, repeating the main analysis using a multiple imputation model (n=7004) (SGA, OR 1.22 (0.95-1.56); LGA, OR 1.04 (0.83-1.29)) was also compatible with no clear associations seen.

For peer review only

DISCUSSION

Literature supports that parents' perceptions on their child's development have shown significant consistency with standardised developmental objective measures in

preterm- and term-born infants, and increasingly used in developmental screening for at risk. [33]–[41] Example of such is the validated PARCA-R (Parent Report of Children's Abilities) for very-preterm infants at 2 years.[42] Although some studies have reported variation between parental estimates and objective measures of speech development, parent reported speech outcomes especially speech intelligibility still remains widely used in clinical practice.[43]–[45]

The production of speech is complex, relying on not only intact cognitive, motor and sensory functions and can be complicated by heterogeneity of hearing loss, particularly the child's age at time of hearing loss.[11], [33] Reassuringly, in this work we were unable to identify clear associations between SGA, or LGA infants and adverse parent-reported speech outcomes in early childhood; in either the unadjusted or adjusted models as well as in the analysis of hearing problems. Also reassuringly, this study also did not find an increased risk of adverse parent-reported behavioural and hand problems in SGA and LGA infants, movement problems in SGA infants and learning difficulties in LGA infants. However, confidence intervals are relatively wide and important increases in morbidity cannot be excluded and more work with precise estimate may be warranted.

Evidence from this study suggest that SGA infants may have higher risks of parent-reported learning difficulties in early childhood but not behavioural, hearing, movement or hand. A single-centre cohort study of term infants in Australia born to women presenting antenatally between 1981 and 1984 showed that children born SGA had significantly more learning difficulties when followed up at 14 years of age using a parent-reported survey and academic achievement test. This study reported a

For peer review only

comparable 19.8% and 20.5% incidence for those who completed psychometric testing and behavioural questionnaires respectively. This study also demonstrated long-term attention difficulties in extremely SGA (3rd centile and below) term-born female adolescents.[46] Similar findings of poorer attention, executive function and memory were reported in a small cohort study of SGA preterm- and term-born young adults who underwent neuropsychological assessment.[47]

There was also evidence suggesting that LGA infants have a small increased risk of parent-reported movement problems during early childhood. The potential increased risk in LGA infants could be influenced by neonatal baseline comorbidities such as poor Apgar scores at birth, shoulder dystocia and hypoxic-ischaemic encephalopathy; all diseases seen more comonly in larger infants. These comorbidities were unavailable for this study population and could be investigated in subsequent studies to identify possible causal relationships that might underpin this association. However hand problems were not evident in this subgroup, as expected with the increased risk of shoulder dystocia in LGA infants and so interpretation of this association should be done cautiously.[6]

Analysis of baseline characteristics noted a significant difference in maternal age, and demographics at birth between birthweight centile categories. While this study was not designed to explore the relationship between maternal age and birthweight centile categories, previous studies have reported significant findings of higher maternal age in LGA infants.[29]–[32] In addition, the association between maternal smoking, deprivation, and birthweight centile categories was sobering, with 20% of SGA infants exposed to in-utero smoking. In this work, more than 1 in 5 mothers smoked during

For peer review only - http://bmjopen.bmj.com/site/about/guidelines.xhtml

pregnancy, identifying an important public health focus on a modifiable risk factor for low birth weight to address,[27] with even first trimester cessation of smoking having substantial benefits.[27], [48], [49]

There was also a significant difference in the mode of delivery across birthweight centile categories likely due to clinical practice managing high-risk pregnancies[16], [25], [26]. This may include singleton or multiple pregnancies, gestational age and estimated birthweight or centile at time of birth. Being male or female did not influence being SGA or LGA, compatible with findings of a large cohort study in Netherlands.[24]

For peer review only

CONCLUSIONS

In this work, infants born small (SGA) or large for gestational age (LGA) do not have higher risks of speech problems in childhood when compared to infants with birthweights appropriate for gestational age (AGA). However important differences may still exist, and we found that some infants being born LGA may have increased movement problems, while those born SGA may have long-term learning difficulties; compared to AGA infants

DECLARATIONS

Funding

No additional funding was received for this analysis. The original study, RANOPs was funded by Medical Research Council Experimental Medicine Challenge Grant (reg: MR/M022552/1), Mason Medical Research Foundation grant, and Children and Young Peoples Research Network Wales.

Competing interests

For peer review only - http://bmjopen.bmj.com/site/about/guidelines.xhtml

The authors have no relevant financial or non-financial interests to disclose.

Acknowledgements

We would like to thank all participants in this study.

Author contributions

Gabrielle Jee conceived and designed this work, wrote statistical analysis plan, cleaned, and analysed data, drafted first manuscript, revised draft manuscript and edited and updated final paper.

David Odd conceived and designed this work, wrote statistical analysis plan, analysed data and drafted first manuscript, revised draft manuscript and edited and updated final paper

Sarah J. Kotecha and Sailesh Kotecha conceived, designed, performed original RANOPs study including data collection, revised draft manuscript and edited and updated final paper.

Mallinath Chakraborty revised the draft manuscript and edited and updated final paper.

Ethical approval

Ethical approval was sought at initiation of RANOPs and approved by South East Wales Research Ethics Committee (Research Ethics Committee 12/WA/0155 Project 91349). Parents provided written consent to participate.

Data sharing

All data must be held securely at Cardiff University as per ethical approval given for this research. Anonymous data will be available from the department of Child Health

For peer review only - http://bmjopen.bmj.com/site/about/guidelines.xhtml

at Cardiff University to bona fide researchers provided that ethical approval is obtained from a research ethics committee in the UK for any suggested studies. Requests for data access should be sent to Sailesh Kotecha (Kotechas@cardiff.ac.uk).

For peer review only

REFERENCES

[1] G. Chiossi, C. Pedroza, M. M. Costantine, V. T. T. Truong, G. Gargano, and G. R. Saade, "Customized *vs* population-based growth charts to identify neonates at risk of adverse outcome: systematic review and Bayesian meta-analysis of observational studies," *Ultrasound in Obstetrics & Gynecology*, vol. 50, no. 2, Aug. 2017, doi: 10.1002/uog.17381.

[2] J. A. Hutcheon, X. Zhang, R. W. Platt, S. Cnattingius, and M. S. Kramer, "The case against customised birthweight standards," *Paediatr Perinat Epidemiol*, vol. 25, no. 1, Jan. 2011, doi: 10.1111/j.1365-3016.2010.01155.x.

[3] R. J. Baer *et al.*, "Population-based risks of mortality and preterm morbidity by gestational age and birth weight," *Journal of Perinatology*, vol. 36, no. 11, Nov. 2016, doi: 10.1038/jp.2016.118.

[4] R. E. Ghosh, J. D. Berild, A. F. Sterrantino, M. B. Toledano, and A. L. Hansell, "Birth weight trends in England and Wales (1986–2012): babies are getting heavier," *Arch Dis Child Fetal Neonatal Ed*, vol. 103, no. 3, pp. F264–F270, May 2018, doi: 10.1136/archdischild-2016-311790.

[5] V. Chiavaroli, J. G. B. Derraik, P. L. Hofman, and W. S. Cutfield, "Born Large for Gestational Age: Bigger Is Not Always Better," *J Pediatr*, vol. 170, pp. 307–311, Mar. 2016, doi: 10.1016/j.jpeds.2015.11.043.

[6] S. L. Boulet, G. R. Alexander, H. M. Salihu, and M. Pass, "Macrosomic births in the united states: Determinants, outcomes, and proposed grades of risk," *Am J Obstet Gynecol*, vol. 188, no. 5, pp. 1372–1378, May 2003, doi: 10.1067/mob.2003.302.

[7] A. Z. Khambalia, C. S. Algert, J. R. Bowen, R. J. Collie, and C. L. Roberts, "Long-term outcomes for large for gestational age infants born at term," *J Paediatr Child Health*, vol. 53, no. 9, Sep. 2017, doi: 10.1111/jpc.13593.

[8] S. Dollberg, "Normoblasts in large for gestational age infants," *Arch Dis Child Fetal Neonatal Ed*, vol. 83, no. 2, Sep. 2000, doi: 10.1136/fn.83.2.F148.

[9] N. S. Boghossian, M. Geraci, E. M. Edwards, and J. D. Horbar, "In-Hospital Outcomes in Large for Gestational Age Infants at 22-29 Weeks of Gestation," *J Pediatr*, vol. 198, Jul. 2018, doi: 10.1016/j.jpeds.2018.02.042.

[10] G. S. Moore, A. W. Kneitel, C. K. Walker, W. M. Gilbert, and G. Xing, "Autism risk in small- and large-for-gestational-age infants," *Am J Obstet Gynecol*, vol. 206, no. 4, pp. 314.e1-314.e9, Apr. 2012, doi: 10.1016/j.ajog.2012.01.044.

[11] P. Tremblay, I. Deschamps, and V. L. Gracco, "Neurobiology of Speech Production," in *Neurobiology of Language*, Elsevier, 2016, pp. 741–750. doi: 10.1016/B978-0-12-407794-2.00059-6.

[12] M. O. Edwards, S. J. Kotecha, J. Lowe, L. Richards, W. J. Watkins, and S. Kotecha, "Early-term birth is a risk factor for wheezing in childhood: A cross-sectional population study," *Journal of Allergy and Clinical Immunology*, vol. 136, no. 3, pp. 581-587.e2, Sep. 2015, doi: 10.1016/j.jaci.2015.05.005.

[13] M. O. Edwards, S. J. Kotecha, J. Lowe, L. Richards, W. J. Watkins, and S. Kotecha, "Management of Prematurity-Associated Wheeze and Its Association with Atopy," *PLoS One*, vol. 11, no. 5, May 2016, doi: 10.1371/journal.pone.0155695.

[14] J. Lowe, S. J. Kotecha, W. J. Watkins, and S. Kotecha, "Effect of fetal and infant growth on respiratory symptoms in preterm-born children," *Pediatr Pulmonol*, vol. 53, no. 2, pp. 189–196, Feb. 2018, doi: 10.1002/ppul.23920.

[15] H. Pan and T. Cole, "LMSgrowth, a Microsoft Excel add-in to access growth references based on the LMS method; Version 2.77." 2012. [Online]. Available: http://wwwhealthforallchildrencouk/

For peer review only

For peer review only - http://bmjopen.bmj.com/site/about/guidelines.xhtml

[16] Royal College of Obstetricians & Gynaecologists, “The Investigation and Management of the Small-for-Gestational-Age Fetus. Green-top Guideline No.31. 2nd Edition,” Jan. 2014.

[17] Public Health Scotland, “Appropriate Birthweight for Gestational Age,” Nov. 2020. https://www.opendata.nhs.scot/hu/dataset/births-in-scottish-hospitals/resource/a5d4de3f-e340-455f-b4e4-e26321d09207 (accessed Oct. 29, 2021).

[18] World Health Organisation, “Preterm Birth,” Feb. 19, 2018. https://www.who.int/news-room/fact-sheets/detail/preterm-birth (accessed Oct. 29, 2019).

[19] The American College of Obstetricians and Gynecologists Committee on Obstetric Practice Society for Maternal-Fetal Medicine, “Committee Opinion No 579: Definition of Term Pregnancy,” *Obstetrics & Gynecology*, vol. 122, no. 5, Nov. 2013, doi: 10.1097/01.AOG.0000437385.88715.4a.

[20] Statistics for Wales, “Welsh Index of Multiple Deprivation,” 2019. https://statswales.gov.wales/Catalogue/Community-Safety-and-Social-Inclusion/Welsh-Index-of-Multiple-Deprivation (accessed Jan. 31, 2022).

[21] T. N. Basit, A. Hughes, Z. Iqbal, and J. Cooper, “The influence of socio-economic status and ethnicity on speech and language development,” *Int J Early Years Educ*, vol. 23, no. 1, pp. 115–133, Jan. 2015, doi: 10.1080/09669760.2014.973838.

[22] M. Mohsin, F. Wong, A. Bauman, and J. Bai, “Maternal and Neonatal Factors Influencing Premature Birth and Low Birth Weight in Australia,” *J Biosoc Sci*, vol. 35, no. 2, pp. 161–174, Apr. 2003, doi: 10.1017/S0021932003001615.

[23] M. Gielen *et al.*, “Secular trends in gestational age and birthweight in twins,” *Human Reproduction*, vol. 25, no. 9, pp. 2346–2353, Sep. 2010, doi: 10.1093/humrep/deq160.

[24] B. J. Voskamp *et al.*, “Association between fetal sex, birthweight percentile and adverse pregnancy outcome,” *Acta Obstet Gynecol Scand*, vol. 99, no. 1, pp. 48–58, Jan. 2020, doi: 10.1111/aogs.13709.

[25] Royal College of Obstetricians and Gynaecologists, “Shoulder Dystocia,” *Green-top Guideline No.42, 2nd Edition*, 2012. https://www.rcog.org.uk/globalassets/documents/guidelines/gtg_42.pdf (accessed Jan. 11, 2022).

[26] “Management of Breech Presentation,” *BJOG*, vol. 124, no. 7, pp. e151–e177, Jun. 2017, doi: 10.1111/1471-0528.14465.

[27] C. Johnson, S. Jones, and S. Paranjothy, “Low Birth Weight Review,” Jul. 2014, Accessed: Jan. 31, 2022. [Online]. Available: https://www2.nphs.wales.nhs.uk/ChildrenMatFamiliesDocs.nsf/($all)/E3F761EC6EFE646F80257D490044FBAE/$file/Low%20Birth%20Weight%20-%20technical%20paper%20v1.pdf?OpenElement

[28] F. McAndrew, J. Thompson, L. Fellows, A. Large, M. Speed, and M. Renfrew, “Infant Feeding Survey 2010,” 2010. Accessed: Jan. 31, 2022. [Online]. Available: https://sp.ukdataservice.ac.uk/doc/7281/mrdoc/pdf/7281_ifs-uk-2010_report.pdf

[29] A. Weissmann-Brenner *et al.*, “Maternal and neonatal outcomes of large for gestational age pregnancies,” *Acta Obstet Gynecol Scand*, vol. 91, no. 7, pp. 844–849, Jul. 2012, doi: 10.1111/j.1600-0412.2012.01412.x.

[30] W. Siggelkow, D. Boehm, C. Skala, M. Grosslercher, M. Schmidt, and H. Koelbl, “The influence of macrosomia on the duration of labor, the mode of delivery and intrapartum complications,” *Arch Gynecol Obstet*, vol. 278, no. 6, pp. 547–553, Dec. 2008, doi: 10.1007/s00404-008-0630-7.

For peer review only

[31] Y. Akin, S. Cömert, C. Turan, A. Piçak, T. Ağzikuru, and B. Telatar, “Macrosomic newborns: a 3-year review.,” *Turk J Pediatr*, vol. 52, no. 4, pp. 378–83.
[32] R. Bakker, E. Steegers, A. Biharie, J. Mackenbach, A. Hofman, and V. Jaddoe, “Explaining differences in birth outcomes in relation to maternal age: the Generation R Study,” *BJOG*, vol. 118, no. 4, pp. 500–509, Mar. 2011, doi: 10.1111/j.1471-0528.2010.02823.x.
[33] N. D. Emerson, H. E. R. Morrell, and C. Neece, “Predictors of Age of Diagnosis for Children with Autism Spectrum Disorder: The Role of a Consistent Source of Medical Care, Race, and Condition Severity,” *J Autism Dev Disord*, vol. 46, no. 1, pp. 127–138, Jan. 2016, doi: 10.1007/s10803-015-2555-x.
[34] M. J. Vincer, H. Cake, M. Graven, L. Dodds, S. McHugh, and T. Fraboni, “A Population-Based Study to Determine the Performance of the Cognitive Adaptive Test/Clinical Linguistic and Auditory Milestone Scale to Predict the Mental Developmental Index at 18 Months on the Bayley Scales of Infant Development-II in Very Preterm Infants,” *Pediatrics*, vol. 116, no. 6, pp. e864–e867, Dec. 2005, doi: 10.1542/peds.2005-0447.
[35] H.-F. Liao, T.-M. Wang, G. Yao, and W.-T. Lee, “Concurrent validity of the Comprehensive Developmental Inventory for Infants and Toddlers with the Bayley Scales of Infant Development-II in preterm infants.,” *J Formos Med Assoc*, vol. 104, no. 10, pp. 731–7, Oct. 2005.
[36] C. Skellern, Y. Rogers, and M. O’Callaghan, “A parent-completed developmental questionnaire: Follow up of ex-premature infants,” *J Paediatr Child Health*, vol. 37, no. 2, pp. 125–129, Apr. 2001, doi: 10.1046/j.1440-1754.2001.00604.x.
[37] K. B. Doig, M. M. Macias, C. F. Saylor, J. R. Craver, and P. E. Ingram, “The Child Development Inventory: A developmental outcome measure for follow-up of the high-risk infant,” *J Pediatr*, vol. 135, no. 3, pp. 358–362, Sep. 1999, doi: 10.1016/S0022-3476(99)70134-4.
[38] J. Squires, D. Bricker, and L. Potter, “Revision of a Parent-Completed Developmental Screening Tool: Ages and Stages Questionnaires,” *J Pediatr Psychol*, vol. 22, no. 3, pp. 313–328, 1997, doi: 10.1093/jpepsy/22.3.313.
[39] R. Bortolus, F. Parazzini, D. Trevisanuto, S. Cipriani, P. Ferrarese, and V. Zanardo, “Developmental assessment of preterm and term children at 18 months: reproducibility and validity of a postal questionnaire to parents,” *Acta Paediatr*, vol. 91, no. 10, pp. 1101–1107, Oct. 2002, doi: 10.1080/080352502760311610.
[40] K. Sonnander, “PARENTAL DEVELOPMENTAL ASSESSMENT OF 18-MONTH-OLD CHILDREN: RELIABILITY AND PREDICTIVE VALUE,” *Dev Med Child Neurol*, vol. 29, no. 3, pp. 351–362, Nov. 2008, doi: 10.1111/j.1469-8749.1987.tb02489.x.
[41] E. Prochazkova, V. Brichacek, J. Dittrichova, Sobotkova D, and J. Vondracek, “Parents’ concerns regarding infant development: Kent Infant Development (KID) Scale,” *Stud Psychol (Bratisl)*, vol. 39, pp. 263–265, 1997.
[42] A. J. Martin *et al.*, “Performance of the Parent Report of Children’s Abilities-Revised (PARCA-R) versus the Bayley Scales of Infant Development III,” *Arch Dis Child*, vol. 98, no. 12, pp. 955–958, Dec. 2013, doi: 10.1136/archdischild-2012-303288.
[43] C. E. Venker, E. Haebig, J. Edwards, J. R. Saffran, and S. Ellis Weismer, “Brief Report: Early Lexical Comprehension in Young Children with ASD: Comparing Eye-Gaze Methodology and Parent Report,” *J Autism Dev Disord*, vol. 46, no. 6, pp. 2260–2266, Jun. 2016, doi: 10.1007/s10803-016-2747-z.
[44] A. Nordahl-Hansen, A. Kaale, and S. E. Ulvund, “Language assessment in children with autism spectrum disorder: Concurrent validity between report-based

For peer review only - http://bmjopen.bmj.com/site/about/guidelines.xhtml

assessments and direct tests," *Res Autism Spectr Disord*, vol. 8, no. 9, pp. 1100–1106, Sep. 2014, doi: 10.1016/j.rasd.2014.05.017.

[45] A. Sakash, T. Mahr, and K. C. Hustad, "Validity of Parent Ratings of Speech Intelligibility for Children with Cerebral Palsy," *Dev Neurorehabil*, vol. 24, no. 2, pp. 98–106, Feb. 2021, doi: 10.1080/17518423.2020.1830447.

[46] M. J. O'Keeffe, M. O'Callaghan, G. M. Williams, J. M. Najman, and W. Bor, "Learning, Cognitive, and Attentional Problems in Adolescents Born Small for Gestational Age," *Pediatrics*, vol. 112, no. 2, pp. 301–307, Aug. 2003, doi: 10.1542/peds.112.2.301.

[47] H. F. Østgård *et al.*, "Neuropsychological Deficits in Young Adults Born Small-for-Gestational Age (SGA) at Term," *Journal of the International Neuropsychological Society*, vol. 20, no. 3, pp. 313–323, Mar. 2014, doi: 10.1017/S1355617714000034.

[48] V. W. v. Jaddoe *et al.*, "Active and passive maternal smoking during pregnancy and the risks of low birthweight and preterm birth: the Generation R Study," *Paediatr Perinat Epidemiol*, vol. 22, no. 2, pp. 162–171, Mar. 2008, doi: 10.1111/j.1365-3016.2007.00916.x.

[49] K. Raatikainen, P. Huurinainen, and S. Heinonen, "Smoking in early gestation or through pregnancy: A decision crucial to pregnancy outcome," *Prev Med (Baltim)*, vol. 44, no. 1, pp. 59–63, Jan. 2007, doi: 10.1016/j.ypmed.2006.07.017.

For peer review only

Figure 1. Proportion of preterm, and term infants with parent-reported speech problems in early childhood by birthweight centile (Gaussian smoothed).

Figure 2. Proportion of neurodevelopmental disorders stratified by gestation age at birth, birthweight centile category, and WIMD category (quintile).

For peer review only

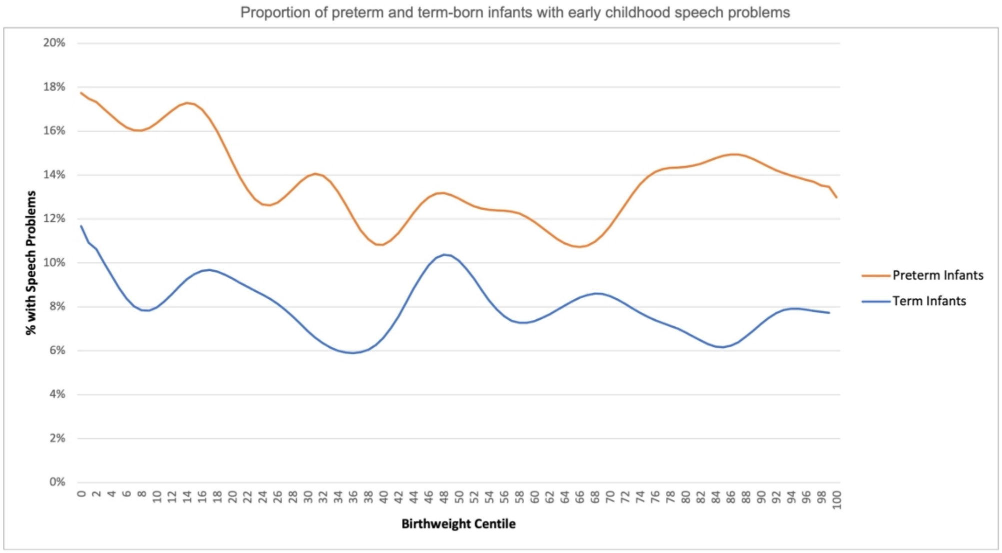

Figure 1. Proportion of preterm, and term infants with parent-reported speech problems in early childhood by birthweight centile (Gaussian smoothed).

323x179mm (300 x 300 DPI)

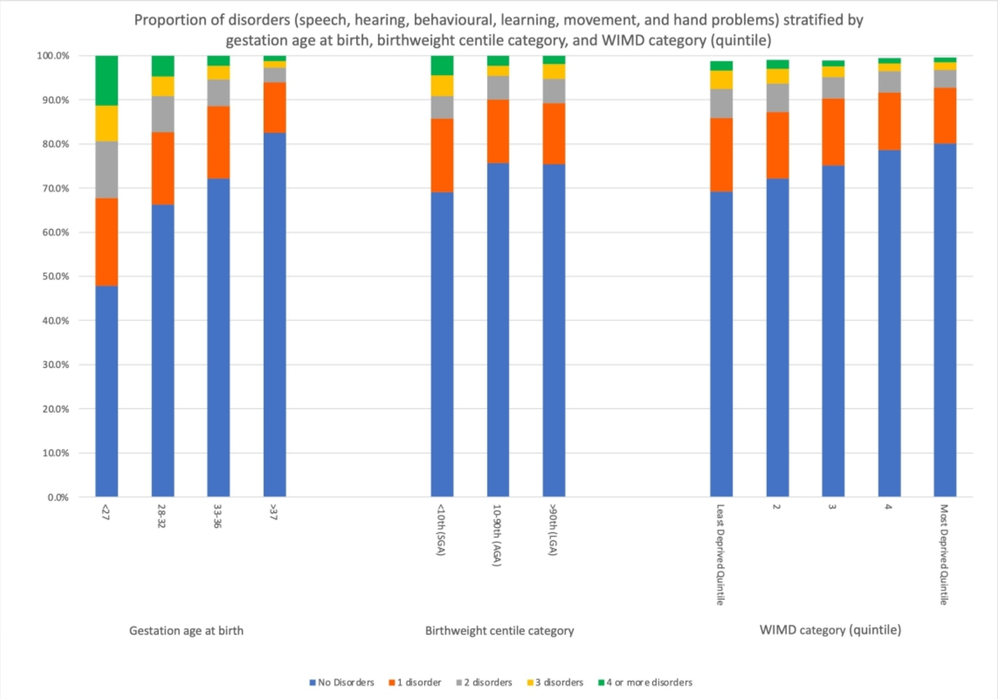

Figure 2. Proportion of neurodevelopmental disorders stratified by gestation age at birth, birthweight centile category, and WIMD category (quintile).

272x190mm (300 x 300 DPI)

Ethics protocol Version 1.1 5th November 2012

Respiratory and neurological outcomes in children born preterm study (RANOPs)

Research team:

Surname	First name	Title	Post held
Edwards	Martin	Dr	Clinical Research Fellow<br>Cardiff University
Kotecha	Sailesh	Professor	Professor of Child Health (CI for study)<br>Cardiff University
Dunstan	Frank	Professor	Professor of Medical Statistics<br>Cardiff University
Henderson	John	Professor	Professor of Paediatric Respiratory Medicine<br>Bristol University
Watkins	John	Dr	Medical Statistician<br>Cardiff University
John	Gareth	Mr	Improvement and Innovations Manager<br>NWIS
Richards	Louise	Mrs	Information Technology Support<br>NWIS
Morris	Geraint	Dr	Consultant Paediatrician *(Local PI)*<br>Abertawe Bro Morgannwg University Health Board
Papworth	Sue	Dr	Consultant Neonatologist *(Local PI)*<br>Aneurin Bevan Health Board
Stutchfield	Peter	Dr	Consultant Paediatrician *(Local PI)*<br>Betsi Cadwaladr University Health Board
Al-Muzaffar	Iyad	Dr	Consultant Neonatal Paediatrician *(Local PI)*<br>Cwm Taf Health Board
Pitchaikani	Prem	Dr	Consultant Paediatrician *(Local PI)*<br>Hywel Dda Health Board
Prosser	Ingrid	Dr	Consultant Community Paediatrician *(Local PI)*<br>Powys Teaching Health Board

1. *Background:*

Babies born late preterm, between 33 and 36 weeks gestation (6-7% of all UK births), have a greater risk of developing breathing problems than previously appreciated. Our research group has recently shown that late preterm born 8-9 year olds have worse lung function than similarly aged children born at term but their lung function improves by 14-17 years of age (http://www.bbc.co.uk/news/uk-wales-south-east-wales-15071922).[1] In 2009 in England and Wales there were 706,248 live-births and over 42,000 were born late preterm.[2, 3] This group of children born late preterm tends to be treated the same as children born full term however there may be a need to monitor this group of children more closely.

Definition of terms:

Gestational groups - ≤32 weeks gestation = extremely preterm
- 33-36 weeks gestation = late preterm
- ≥37 weeks gestation = term

1.1 Research Question

We hypothesise that children born extremely preterm (23-32 weeks gestation) and late preterm (33 to 36 weeks gestation):

(a) have increased respiratory symptoms and disease,
(b) have increased neurodevelopmental problems,
(c) have increased health care utilisation, mainly due to respiratory morbidity

in infancy, in the pre-school and early school years, when compared to age-matched term-born children (control group).

For peer review only - http://bmjopen.bmj.com/site/about/guidelines.xhtml

1.2 Methods

Study Design: The morbidity and health utilisation of late preterm infants

In Wales, the average number of live-born deliveries over the last 5 years is 34,464 per annum.[2] Approximately 2,000 each year are born prematurely with 500 born extremely preterm and 1,500 late preterm.[2]

AWPS data: Total number of survivors at 1 year of age per year of birth and gestational age in Wales						
Year of birth	2003	2005	2007	2009	2010	2011
Gestational age						
23-32 weeks	436	507	503	471	513	492
33-36 weeks	1781	1719	1976	1980	1926	1913
37-43 weeks	26448	30063	31545	31933	33277	32819
Other/unknown	2303	226	279	323	171	334
Total	30968	32515	34303	34707	35887	35558

For late preterm infants, we will address:

(a) respiratory morbidity,

(b) neurodevelopmental problems and

(c) health care utilisation, especially due to respiratory reasons, via a questionnaire study and also the well-linked health databases in Wales with the collaboration of local neonatologists.

The data will be compared to term born and extremely preterm-born groups. Our team, including Professor John Henderson as well as Professor Frank Dunstan, has great expertise in questionnaire studies assessing health of children and adults.[4, 5] A modified ISAAC questionnaire focussing on respiratory symptoms (rather than allergy) will be used to assess respiratory health including wheezing, drug usage and physical activity as well as visits to hospitals and general practitioners in school-aged children[6] and a validated questionnaire by Powell et al will be used for preschool children .[7, 8] The questionnaires will be used to assess respiratory health and for any neurodevelopmental difficulties in a cross-sectional survey of children aged 1, 2, 3, 5, 7 and 9 years. The questionnaires will allow us to assess the impact of late preterm birth on respiratory outcomes, developmental delay and health service utilisation. The questionnaires will be age-appropriate (one for <5 years of age and another for 5-9 years of age). The two age-appropriate questionnaires are based on previous extensively validated questionnaires for both respiratory symptoms and neurodevelopmental outcomes in both preschool and school-aged children.[6-11]

We shall send 2,000 questionnaires with an invitation letter and parent information sheet for each age-group for the preterm groups (including 1,500 late preterm- and 500 extremely preterm-born children) together with a similar number for age-matched term controls. The matching of the term controls will be based on sex, place of birth and chronological age (DOB), although in the analysis of the data, the corrected postnatal age (this is the age a premature baby would be if born at term) of the preterm infants will be taken into account as a potential confounding factor. The total of 24,000 questionnaire packs will be sent via the NHS Wales Informatics Service (NWIS), which have expertise for identifying subjects from the NHS administrative register and then providing DST, the mailing house with a database to send out the questionnaires. Furthermore, children who have moved from the area or have died are identified and excluded, by using data from the All Wales Perinatal Survey (AWPS) which is directed by Professor Sailesh Kotecha with Professor Frank Dunstan a member of the steering committee.[2] AWPS maintains data on children who die before their first birthday, and this data will be used along with the Welsh Demographics Service, which also holds information on children who have passed away.

For peer review only - http://bmjopen.bmj.com/site/about/guidelines.xhtml

Prior to any reminder packs being sent out to the families, NWIS will be up-dated of all families who have returned a questionnaire pack. NWIS will then up-date the contact data base and also repeat the safe guard of who has died, and then send the new data base of contact information to DST, who will send out the reminder packs (1st after 1 month from the initial mailing date and 2nd after 3-4 months).

Secondly, using linked databases (including the Patient Episode Database for Wales, PEDW and the National Community Child Health Database, NCCHD and the Welsh Demographics Service, WDS) for hospital episodes, we will identify the general health service usage of these children. For those children with a returned, signed (consented) questionnaire we will be able to link the hospital data to their completed questionnaire to allow for more accurate analysis. The information gathered from PEDW, NCCHD and WDS will also contain data on all children less than 10 years of age in Wales (anonymised) and this will allow us to determine the representativeness of the sample returning questionnaires. The social status of a family will be determined by location using the welsh index for multiple deprivation child index score 2011 (taken from WDS).

The linked cross sectional data at 5 age points will be summarised in terms of rates of wheezing, rates of hospital admissions, etc. The exploration of the data will be hypothesis led to investigate if being born late preterm has an effect on the health outcome of children when compared to term born controls. The health outcomes will include hospital admissions, visits to GP, wheezing episodes, use of prescribed medication, doctor diagnosed conditions, long term disability and activity levels. The formal analysis will use general linear models to compare rates between different gestational age groups adjusting for confounders such as social class, maternal smoking during pregnancy, birth weight, gender etc.

Sample size: For a question with binary response (e.g. symptom present or absent), assuming a 50-60% response rate to the questionnaire, we will have 95% power for identifying a difference between the preterm- and term-born children if the true symptom rate is 15% in preterm and 10% in term children. We have estimated the response rates based on previous similar studies in Wales.[4, 12]

2. NWIS collaboration

We have already liaised with NWIS and shall ask their team to identify children born between 1/01/2003 to 31/12/2011. NWIS will identify all preterm infants (<37 weeks of gestation at birth) for each age group (1,2,3,5,7 & 9 years of age) using the national database (NCCHD and WDS). NWIS will then select the term controls by identifying gender matched children born on the same day and in the same area (hospital or midwifery led unit) as each preterm infant. So there will be approximately 2,000 term controls for each age group. For each age (1,2,3,5,7 and 9 years of age) there will be three groups; children born at full term (37-43 weeks of gestation), children born late preterm (33-36 weeks of gestation) and children born extremely preterm (≤32 weeks of gestation). Each child will be assigned a study number based on their DOB/gestational age/a unique reference number.

The information obtained from the health databases (WDS, NCCHD and PEDW) for each child will include the child's name, current address, gestational age, DOB, birth weight, breast feeding at birth and at 8 weeks of age, welsh index deprivation score, hospital admissions of the last year and discharge diagnosis. The postcode for the current address will be used to identify the local health board (so that the appropriate local PI is identified on the invitation letter being sent to the family). This information will need to be checked to remove any children that have died via AWPS and the WDS database. This will involve AWPS sending data to NWIS with the name, DOB and address of each child that has passed away over the last 10 years (1/1/03 to 31/12/12). ***None of this data will be made available to the research team.*** NWIS will supply DST, the mailing house with a study

For peer review only - http://bmjopen.bmj.com/site/about/guidelines.xhtml

database split into two sections (> 5 years of age and > 5 years of age). The database will contain the study ID number, name, address and local health board of each study participant. Data will be transferred between NWIS and DST via File Transfer Protocol Secure (FTPS).

DST will divide each data set into 7 sections according to the local health board in which the child resides. DST will then print the individual questionnaires (including study ID numbers), invitation letters including the family address and signed with the appropriate LHB with details of the local PI, and information sheets and place these in envelopes with windows that allow the letter to show the family address, and these packs will then be sent by royal mail to the families. Stamped addressed envelopes (to our research department) will be included within the study pack. DST also provide a further data health check, informing us of any duplicates and of families who may have moved, thus avoiding unnecessary/inappropriate mailing. ***No personal data will be released by NWIS to the research team at this stage.***

NWIS will send study ID numbers with the child's gestational age to the research team, so that when questionnaires are returned the correct gestational age for the child can be confirmed. Families will be given 4-8 weeks to return the questionnaires. A list of families who have returned questionnaires (by study ID numbers) will be sent to NWIS and they will send further information on each child that has a signed (consented) questionnaires, to include birth weight, antenatal details, hospital of birth, hospital admissions and discharge diagnosis over the last 12 months.

NWIS, following a re-check of the health data base for any deaths, will create a list of any families that have not returned a questionnaire. This up-dated study database will be given to DST and the first reminder pack will be sent to these families at 1 month after the initial pack was sent out. If the response rate remains low then this process will be repeated with a second remainder pack being sent after 3-4 months, when the research team will give NWIS a list of all families who have returned questionnaires and NWIS will collate an up-dated list, having removed any families where a child has died in the interim period since last checking. This up-dated study database will be given to DST and the second reminder pack will be sent to those families who have not yet responded.

Questionnaires will be returned to the Department of Child Health at Cardiff University and processed by automated scanners and specialised software (Remark Office OMR 8). The questionnaires will be coded by the software, which also identifies any problems such as missing information. Any problems detected by the software will be reviewed by the research team. This will form part of the data quality control checks. Anonymised data will be available for the whole cohort to allow us to determine the representativeness of the responders. The returned questionnaires will be processed by Dr Martin Edwards and all data stored securely in the research office at the Department of Child Health based at the University Hospital of Wales, Heath Park, Cardiff. By returning the completed questionnaires it will be assumed that the family is consenting to take part in the study. If the questionnaire form is signed the family will be consenting to be contacted with regards to clarifying any issues with the returned questionnaire; contacted in the future to take part in further research in this area; and for access to patient identifiable information in the health databases to link hospital admission and GP records information with that gathered from the questionnaires. It is clearly stated in the information leaflet what will happen with the data collected.

3. Ethical approval

The project has ethical and R&D approval from within Wales. The global governance check reference is **IRAS 91349** and the Ethics reference is **12/WA/0155.** The sponsor for the project is Cardiff University (**SPON1038-11**).

For peer review only - http://bmjopen.bmj.com/site/about/guidelines.xhtml

4. Timescale
We aim to mail the questionnaires between January and February 2013. We shall allow the families 6 – 8 weeks to respond initially and send a second mailing of questionnaires between April and May 2013 and if necessary a third mailing of questionnaires in between July and August 2013, dependent upon the response rate. Our expectation is to reach a 50 – 60% response rate as has been achieved in many recent questionnaire studies.[4, 12-14]

5. Resources
The study is currently funded by Departmental funds and research grant funding is being sought from several charities that support medical research – decision expected Jan/Feb 2013.

6. Documents
- i. Letter of invitation to parents – 1 side of A4 paper
- ii. Questionnaire for preschool children (<5 years old) – 4 sides of A4 paper
- iii. Questionnaire for school aged children (5-9 years old) – 4 sides of A4 paper
- iv. Information sheet for families – 2 sides of A4 paper
- v. Flow diagram of research protocol

7. References

1. Kotecha, S.J., et al., *Effect of late preterm birth on longitudinal lung spirometry in school age children and adolescents.*Thorax.2012;67:54-61.
2. Kotecha S, et al. All Wales Perinatal Survey – Annual Report 2009. Cardiff University 2011.
3. Office of National Statistics (2009).
4. Burr ML, Wat D, Evans C, Dunstan FD, Doull IJ; BTS Research Committee. Asthma prevalence in 1973, 1988 and 2003. Thorax. 2006;61:296-9
5. Thomas H, et al. Mental health and quality of residential environment. Br J Psychiatry. 2007;191:500-5
6. Asher MI, et al. International study of asthma and allergies in childhood (ISAAC): Rationale and methods. Eur Respir J 1995;8:483-491.
7. Powell CV, McNamara P, Solis A, Shaw NJ. A parent completed questionnaire to describe the patterns of wheezing and other respiratory symptoms in infants and preschool children. *Archives of Disease in Childhood* 2002;87(5):376-9.
8. Trinick, R., K.W. Southern, and P.S. McNamara, *Assessing the Liverpool Respiratory Symptom Questionnaire in children with cystic fibrosis.* The European respiratory journal : official journal of the European Society for Clinical Respiratory Physiology, 2012;39(4):899-905.
9. Greenough A, Giffin FJ, Yuksel B. Respiratory morbidity in preschool children born prematurely. Relationship to adverse neonatal events. *Acta Paediatr* 1996;85(7):772-7.
10. Greenough A, Limb E, Marston L, Marlow N, Calvert S, Peacock J. Risk factors for respiratory morbidity in infancy after very premature birth. *Arch Dis Child Fetal Neonatal Ed* 2005;90(4):F320-3.
11. Jones H, Guildea Z, Stewart, J & Cartlidge, P.The health status questionnaire: Achieving concordance with published disability criteria. Archives of Disease in Childhood, 2002. **86**(1): p. 15-20.
12. Fox, R. and S. Minchom, *Parental experiences of the newborn hearing screening programme in Wales: a postal questionnaire survey.* Health expectations : an international journal of public participation in health care and health policy, 2008;11(4):376-83.
13. Leonardi, N.A., et al., *Validation of the Asthma Predictive Index and comparison with simpler clinical prediction rules.* The Journal of allergy and clinical immunology, 2011; 127(6):1466-72 e6.
14. Turner, S., et al., *First- and second-trimester fetal size and asthma outcomes at age 10 years.* American Journal of Respiratory and Critical Care Medicine, 2011;184(4);407-13.

For peer review only - http://bmjopen.bmj.com/site/about/guidelines.xhtml

Using a PENCIL fill in the circles like this

○ → ●

DO NOT tick or cross the circles.

An eraser can be used to rectify mistakes.

Study No: **12351**

Health questionnaire for preschool children (<5 years old)

Today's date ____________________2012

Person completing the questionnaire:

Mother ○
Father ○
Other ○

Date of birth of child: ______/_________/________

1. Has your child ever had wheezing or whistling in their chest at any time in their life?

Yes ○ No ○ Unsure ○

IF YOU HAVE ANSWERED "NO" PLEASE GO TO QUESTION 4.

2. In the last three months , during the day time (i.e. when awake) does your child:

	Not at all	a few days	some days	most days	every day
a. Wheeze:	○	○	○	○	○
b. Cough:	○	○	○	○	○
c. Short of breath:	○	○	○	○	○
d. Rattly chest:	○	○	○	○	○
e. Snuffly:	○	○	○	○	○

3. In the last three months , during the night time (i.e. when asleep) does your child:

	Not at all	a few nights	some nights	most nights	every night
a. Wheeze:	○	○	○	○	○
b. Cough:	○	○	○	○	○
c. Short of breath:	○	○	○	○	○
d. Rattly chest:	○	○	○	○	○
e. Snore:	○	○	○	○	○

4. How many colds has your child had in the last three months?

None ○ 1 to 3 ○ more than 3 ○ always has a cold ○

5. When my child has had a cold in the last three months, s/he has a:

	Not at all	a few colds	some colds	most colds	every cold
a. Wheeze:	○	○	○	○	○
b. Cough:	○	○	○	○	○
c. Short of breath:	○	○	○	○	○
d. Rattly chest:	○	○	○	○	○

For peer review only

For peer review only - http://bmjopen.bmj.com/site/about/guidelines.xhtml

Study No: **12351**

e. Snuffly:	◯	◯	◯	◯	◯

6. When my child does NOT have a cold, in the last three months, s/he has a:

	Not at all	a few days	some days	most days	every day
a. Wheeze:	◯	◯	◯	◯	◯
b. Cough:	◯	◯	◯	◯	◯
c. Short of breath:	◯	◯	◯	◯	◯
d. Rattly chest:	◯	◯	◯	◯	◯
e. Snuffly:	◯	◯	◯	◯	◯

7. When my child has been more active (e.g. crawling, walking or when excited), in the last three months, s/he has a:

	Not at all	a few days	some days	most days	every day
a. Wheeze:	◯	◯	◯	◯	◯
b. Cough:	◯	◯	◯	◯	◯
c. Short of breath:	◯	◯	◯	◯	◯
d. Rattly chest:	◯	◯	◯	◯	◯

8. Has your child ever been diagnosed with asthma by a doctor?

Yes ◯ No ◯

9. Is there a family history of

	Yes	No
a. Asthma	◯	◯
b. Eczema	◯	◯
c. Hayfever	◯	◯
d. Allergies	◯	◯

10. In the last 12 months, has your child ever used any regular asthma inhalers (pumps) or medicines?

Yes ◯ No ◯

If yes, please provide the name (or colour of the pump) with details of how often used:

____

____

11. In the last 12 months, has your child had any chest infections?

Yes ◯ No ◯

12. In the last 12 months, how many chest infections has your child had?

For peer review only

For peer review only - http://bmjopen.bmj.com/site/about/guidelines.xhtml

Study No: 12351

None ◯ 1 to 3 ◯ 4 to 10 ◯ more than 10 ◯

13. In the last 12 months, how many courses of antibiotics has your child had?
None ◯ 1 to 3 ◯ 4 to 10 ◯ more than 10 ◯

14. In the last 12 months, how many admissions (overnight or longer) has your child had to hospital for breathing problems?
None ◯ 1 to 3 ◯ 4 to 10 ◯ more than 10 ◯

14a. Does the child's mother smoke cigarettes? Yes ◯ No ◯

If yes, how many per day? 1 to 10 ◯ 11 to 20 ◯ more than 20 ◯

If the child's mother smokes did she smoke during the pregnancy? Yes ◯ No ◯

14b. Does the child's father smoke cigarettes? Yes ◯ No ◯

If yes, how many per day? 1 to 10 ◯ 11 to 20 ◯ more than 20 ◯

15. Do any other household members smoke cigarettes? Yes ◯ No ◯

If yes, how many per day for the whole household? 1 to 10 ◯
11 to 20 ◯
more than 20 ◯

(please add up all the cigarettes which are smoked by everyone living in the same household including the mother).

16. a . Has your child ever been diagnosed with any breathing problems (e.g. asthma, CF, TB etc.)?
Yes ◯ No ◯
If yes, please provide some details:
____
____

16. b . Has your child ever been diagnosed with any other conditions (e.g. diabetes, epilepsy etc.)?
Yes ◯ No ◯
If yes, please provide some details:
____
____

17. Is your child on any medication (please list all medicines your child is being given)?
Yes ◯ No ◯
If yes, please provide some details:
____
____

18. Does your child take part in any physical activity every week such as dancing, swimming, cycling ?
Yes ◯ No ◯
If yes, please provide some details of how often and for how long:

For peer review only

For peer review only - http://bmjopen.bmj.com/site/about/guidelines.xhtml

Study No: 12351

19. Does your child have any problems with moving?
Yes ◯ No ◯
If yes, please provide some details:

20. Does your child have any problems with using their hands?
Yes ◯ No ◯
If yes, please provide some details:

21. Does your child have any problems with their speech?
Yes ◯ No ◯
If yes, please provide some details:

22. Does your child have any problems with their vision?
Yes ◯ No ◯
If yes, please provide some details:

23. Does your child have any problems with their hearing?
Yes ◯ No ◯
If yes, please provide some details:

24. Does your child have any problems with their behaviour?
Yes ◯ No ◯
If yes, please provide some details:

25. Does your child have any learning difficulties?
Yes ◯ No ◯
If yes, please provide some details:

Thank you for filling in the form. The following section asks how you are happy for us to use the data or to contact you:

(A) If we need to clarify some of your answers, would you be willing to be contacted?

Yes ◯ No ◯ Please initial the box here ☐

Address, Telephone number &/or email:

For peer review only - http://bmjopen.bmj.com/site/about/guidelines.xhtml

Study No: 12351

(B) Most admissions and GP visits in Wales are stored in computer databases called NCCHD or PEDW. As part of this study, we would also like to study how children in Wales have used their GPs or have had admission to hospitals. Would you be happy for us to use your son's or daughter's records on these databases?

Yes ◯ No ◯ Please initial the box here ☐

(C) We may plan similar studies in the future, would you be willing to be contacted in the future?

Yes ◯ No ◯ Please initial the box here ☐

Name of Child	Name of Parent/Guardian	Date	Signature

Thank you very much for taking the time to fill in the form and for contributing to our research. Could you please send the form to:

Dr Martin Edwards,
Room UGT156
Department of Child Health
School of Medicine
Cardiff University
University Hospital of Wales
Heath Park
Cardiff CF14 4XN

In the enclosed self-addressed envelope.

For peer review only

For peer review only - http://bmjopen.bmj.com/site/about/guidelines.xhtml

Using a **PENCIL** fill in the circles like this ○ → ●

DO NOT tick or cross the circles.

An eraser can be used to rectify mistakes.

Study No:

Health questionnaire for school age children (>5 years old)

Today's date ____________________2012

Person completing the questionnaire: Mother ○ Father ○ Other ○

Date of birth of child: ______/_________/________

1. Has your child ever had wheezing or whistling in their chest at any time in the past?

   Yes ○ No ○ Unsure ○

IF YOU HAVE ANSWERED "NO" PLEASE GO TO QUESTION 10.

2. Has your child had wheezing or whistling in the chest in the last 12 months?

   Yes ○ No ○ Unsure ○

IF YOU HAVE ANSWERED "NO" PLEASE GO TO QUESTION 10.

3. How many attacks of wheezing has your child had in the last 12 months?

   None ○ 1 to 3 ○ 4 to 12 ○ more than 12 ○

4. In the last 12 months, how often, on average, has your child's sleep been disturbed due to wheezing?

   Never woken with wheezing ○

   Less than one night per week ○

   One or more nights per week ○

5. In the last 12 months, has wheezing ever been severe enough to limit your child's speech to only one or two words at a time between breaths?

   Yes ○ No ○

6. Has a doctor ever told you that your child has asthma?

   Yes ○ No ○

7. In the last 12 months, has your child's chest sounded wheezy during or after exercise?

   Yes ○ No ○

8. In the last 12 months, has your child ever used any regular asthma inhalers (pumps) or medicines?

   Yes ○ No ○

   If yes, please provide the name (or colour of the pump) with details of how often used:

   ____

   ____

For peer review only

For peer review only - http://bmjopen.bmj.com/site/about/guidelines.xhtml

Study No:

9. In the last 12 months, has your child had a dry cough at night, apart from a cough associated with a cold or chest infection?
   Yes ◯ No ◯

10. A. Does the child's mother smoke cigarettes?
   Yes ◯ No ◯
   If yes, how many per day? 1 to 10 ◯ 11 to 20 ◯ more than 20 ◯

   If the child's mother smokes did she smoke during the pregnancy? Yes ◯ No ◯

10.B. Does the child's father smoke cigarettes?
   Yes ◯ No ◯

   If yes, how many per day? 1 to 10 ◯ 11 to 20 ◯ more than 20 ◯

11. Do any other household members smoke cigarettes? Yes ◯ No ◯

   If yes, how many per day for the whole household? 1 to 10 ◯
   11 to 20 ◯
   more than 20 ◯
   (please add up all the cigarettes which are smoked by everyone living in the same household including the mother).

12. In the last 12 months, has your child had any chest infections?

   Yes ◯ No ◯

13. In the last 12 months, how many chest infections has your child had?

   None ◯ 1 to 3 ◯ 4 to 10 ◯ more than 10 ◯

14. In the last 12 months, how many courses of antibiotics has your child had?

   None ◯ 1 to 3 ◯ 4 to 10 ◯ more than 10 ◯

15. In the last 12 months, how many admissions (overnight or longer) has your child had to hospital for breathing problems?

   None ◯ 1 to 3 ◯ 4 to 10 ◯ more than 10 ◯

16. Is there a family history of

	Yes	No
a. Asthma	◯	◯
b. Eczema	◯	◯
c. Hayfever	◯	◯
d. Allergies	◯	◯

17. Has your child ever been diagnosed with any breathing problems (e.g. asthma, CF, TB etc.)?

   Yes ◯ No ◯

   If yes, please provide some details:

   ____

   ____

For peer review only - http://bmjopen.bmj.com/site/about/guidelines.xhtml

For peer review only

Study No:

18. Has your child ever been diagnosed with any other conditions (e.g. diabetes, epilepsy etc.)?

Yes ◯ No ◯

If yes, please provide some details:

____

____

19. Is your child on any medication?

Yes ◯ No ◯

If yes, please provide some details:

____

____

20. Does your child take part in any physical activity such as dancing, cycling or swimming?

Yes ◯ No ◯

If yes, please provide some details of how often and for how long:

____

____

21. Does your child have any learning problems?

Yes ◯ No ◯

If yes, please provide some details:

____

____

22. Does your child have any problems with their behaviour?

Yes ◯ No ◯

If yes, please provide some details:

____

____

23. Does your child have an educational statement?

Yes ◯ No ◯

If yes, please provide some details:

____

____

24. Does your child have any problems with moving?

Yes ◯ No ◯

If yes, please provide some details:

____

____

For peer review only

For peer review only - http://bmjopen.bmj.com/site/about/guidelines.xhtml

Study No:

25. Does your child have any problems with writing or using their hands?

Yes ◯ No ◯

If yes, please provide some details:

___

26. Does your child have any problems with speech?

Yes ◯ No ◯

If yes, please provide some details:

___

___

27. Does your child have any problems with their vision?

Yes ◯ No ◯

If yes, please provide some details:

___

___

28. Does your child have any problems with their hearing?

Yes ◯ No ◯

If yes, please provide some details:

___

___

29. Does your child have any problems with feeding?

Yes ◯ No ◯

If yes, please provide some details:

___

___

Thank you for filling in the form. The following section asks how you are happy for us to use the data or to contact you:

(A) If we need to clarify some of your answers, would you be willing to be contacted?

Yes ◯ No ◯ Please initial the box here ☐

Address, Telephone number &/or email:

___

___

For peer review only

For peer review only - http://bmjopen.bmj.com/site/about/guidelines.xhtml

Study No:

(B) Most admissions and GP visits in Wales are stored in computer databases called NCCHD or PEDW. As part of this study, we would also like to study how children in Wales have used their GPs or have had admission to hospitals. Would you be happy for us to use your son's or daughter's records on these databases?

Yes ◯ No ◯ Please initial the box here ☐

(C) We may plan similar studies in the future, would you be willing to be contacted in the future?

Yes ◯ No ◯ Please initial the box here ☐

Name of Child	Name of Parent/Guardian	Date	Signature

Thank you very much for taking the time to fill in the form and for contributing to our research. Could you please send the form to:

Dr Martin Edwards,
Room UGT156
Department of Child Health
School of Medicine
Cardiff University
University Hospital of Wales
Heath Park
Cardiff CF14 4XN

In the enclosed self-addressed envelope.

For peer review only

For peer review only - http://bmjopen.bmj.com/site/about/guidelines.xhtml

Multiple Imputation Model

Imputation was based on the following distributions. 50 imputed sets of data were used and combined using Rubin's rules.[1]

Variable	Imputation command	% missing
Speech Problems	-	0 (0.0%)
Learning Difficulties	logit	24 (0.3%)
Behaviour Problems	logit	41 (0.6%)
Movement Problems	logit	13 (0.2%)
Hand Problems	logit	4 (0.1%)
Maternal Age (years)	ologit	779 (11.1%)
Sex	-	0 (0.0%)
Single/Multiple pregnancy	-	0 (0.0%)
Mode of Delivery	mlogit	2913 (41.6%)
WIMD measure	regress	200 (2.9%)
Smoking Status	Logit	279 (4.0%)
Gestational age at birth	-	0 (0.0%)
Age at survey (years)	ologit	1 (0.01%)
Birthweight centile	-	0 (0.0%)

1. Rubin DB. Inference and missing data. Biometrika. 1976;63:581-92.

For peer review only

STROBE Statement—checklist of items that should be included in reports of observational studies

	Item No.	Recommendation	Page No.	Relevant text from manuscript
**Title and abstract**	1	(*a*) Indicate the study's design with a commonly used term in the title or the abstract	1	
		(*b*) Provide in the abstract an informative and balanced summary of what was done and what was found	2-3	
**Introduction**				
Background/rationale	2	Explain the scientific background and rationale for the investigation being reported	5-6	
Objectives	3	State specific objectives, including any prespecified hypotheses	6	
**Methods**				
Study design	4	Present key elements of study design early in the paper	6-7	
Setting	5	Describe the setting, locations, and relevant dates, including periods of recruitment, exposure, follow-up, and data collection	6-7	
Participants	6	(*a*) *Cohort study*—Give the eligibility criteria, and the sources and methods of selection of participants. Describe methods of follow-up<br>*Case-control study*—Give the eligibility criteria, and the sources and methods of case ascertainment and control selection. Give the rationale for the choice of cases and controls<br>*Cross-sectional study*—Give the eligibility criteria, and the sources and methods of selection of participants	7-8	
		(*b*) *Cohort study*—For matched studies, give matching criteria and number of exposed and unexposed<br>*Case-control study*—For matched studies, give matching criteria and the number of controls per case	7-8	
Variables	7	Clearly define all outcomes, exposures, predictors, potential confounders, and effect modifiers. Give diagnostic criteria, if applicable	8-9	
Data sources/ measurement	8*	For each variable of interest, give sources of data and details of methods of assessment (measurement). Describe comparability of assessment methods if there is more than one group	7	
Bias	9	Describe any efforts to address potential sources of bias	10	
Study size	10	Explain how the study size was arrived at	N/A	Supplement 1

Continued on next page

For peer review only - http://bmjopen.bmj.com/site/about/guidelines.xhtml

Quantitative variables	11	Explain how quantitative variables were handled in the analyses. If applicable, describe which groupings were chosen and why	9-10
Statistical methods	12	(*a*) Describe all statistical methods, including those used to control for confounding	9-10
		(*b*) Describe any methods used to examine subgroups and interactions	9-10
		(*c*) Explain how missing data were addressed	10
		(*d*) *Cohort study*—If applicable, explain how loss to follow-up was addressed<br>*Case-control study*—If applicable, explain how matching of cases and controls was addressed<br>*Cross-sectional study*—If applicable, describe analytical methods taking account of sampling strategy	N/A
		(*e*) Describe any sensitivity analyses	10
**Results**			
Participants	13*	(a) Report numbers of individuals at each stage of study—eg numbers potentially eligible, examined for eligibility, confirmed eligible, included in the study, completing follow-up, and analysed	10-11
		(b) Give reasons for non-participation at each stage	10-11
		(c) Consider use of a flow diagram	N/A
Descriptive data	14*	(a) Give characteristics of study participants (eg demographic, clinical, social) and information on exposures and potential confounders	11
		(b) Indicate number of participants with missing data for each variable of interest	10
		(c) *Cohort study*—Summarise follow-up time (eg, average and total amount)	N/A
Outcome data	15*	*Cohort study*—Report numbers of outcome events or summary measures over time	12
		Case-control study—Report numbers in each exposure category, or summary measures of exposure	N/A
		Cross-sectional study—Report numbers of outcome events or summary measures	N/A
Main results	16	(*a*) Give unadjusted estimates and, if applicable, confounder-adjusted estimates and their precision (eg, 95% confidence interval). Make clear which confounders were adjusted for and why they were included	10-11
		(*b*) Report category boundaries when continuous variables were categorized	10-13
		(*c*) If relevant, consider translating estimates of relative risk into absolute risk for a meaningful time period	N/A

Continued on next page

For peer review only - http://bmjopen.bmj.com/site/about/guidelines.xhtml

Other analyses	17	Report other analyses done—eg analyses of subgroups and interactions, and sensitivity analyses	14
**Discussion**			
Key results	18	Summarise key results with reference to study objectives	14-17
Limitations	19	Discuss limitations of the study, taking into account sources of potential bias or imprecision. Discuss both direction and magnitude of any potential bias	14-17
Interpretation	20	Give a cautious overall interpretation of results considering objectives, limitations, multiplicity of analyses, results from similar studies, and other relevant evidence	14-17
Generalisability	21	Discuss the generalisability (external validity) of the study results	14-17
**Other information**			
Funding	22	Give the source of funding and the role of the funders for the present study and, if applicable, for the original study on which the present article is based	17

*Give information separately for cases and controls in case-control studies and, if applicable, for exposed and unexposed groups in cohort and cross-sectional studies.

Note: An Explanation and Elaboration article discusses each checklist item and gives methodological background and published examples of transparent reporting. The STROBE checklist is best used in conjunction with this article (freely available on the Web sites of PLoS Medicine at http://www.plosmedicine.org/, Annals of Internal Medicine at http://www.annals.org/, and Epidemiology at http://www.epidem.com/). Information on the STROBE Initiative is available at www.strobe-statement.org.

For peer review only

For peer review only - http://bmjopen.bmj.com/site/about/guidelines.xhtml

BMJ Open

Early childhood parent-reported speech problems in small and large for gestational age term- and preterm-born infants: A cohort study

Journal:	*BMJ Open*
Manuscript ID	bmjopen-2022-065587.R2
Article Type:	Original research
Date Submitted by the Author:	18-Mar-2023

Complete List of Authors:	Jee, Gabrielle; University of Wales Hospital, Paediatrics<br>Kotecha, Sarah; Cardiff University School of Medicine, Department of Child Health<br>Chakraborty, Mallinath; Cardiff University School of Medicine, Department of Child Health; University of Wales Hospital, Department of Neonatology<br>Kotecha, Sailesh; Cardiff University School of Medicine, Department of Child Health; University of Wales Hospital, Department of Neonatology<br>Odd, David; University Hospital of Wales, Department of Neonatology; Cardiff University School of Medicine, Department of Population Health

**Primary Subject Heading**:	Paediatrics
Secondary Subject Heading:	Neurology
Keywords:	NEONATOLOGY, Developmental neurology & neurodisability < PAEDIATRICS, Neonatal intensive & critical care < INTENSIVE & CRITICAL CARE

SCHOLARONE™
Manuscripts

I, the Submitting Author has the right to grant and does grant on behalf of all authors of the Work (as defined in the below author licence), an exclusive licence and/or a non-exclusive licence for contributions from authors who are: i) UK Crown employees; ii) where BMJ has agreed a CC-BY licence shall apply, and/or iii) in accordance with the terms applicable for US Federal Government officers or employees acting as part of their official duties; on a worldwide, perpetual, irrevocable, royalty-free basis to BMJ Publishing Group Ltd ("BMJ") its licensees and where the relevant Journal is co-owned by BMJ to the co-owners of the Journal, to publish the Work in this journal and any other BMJ products and to exploit all rights, as set out in our licence.

The Submitting Author accepts and understands that any supply made under these terms is made by BMJ to the Submitting Author unless you are acting as an employee on behalf of your employer or a postgraduate student of an affiliated institution which is paying any applicable article publishing charge ("APC") for Open Access articles. Where the Submitting Author wishes to make the Work available on an Open Access basis (and intends to pay the relevant APC), the terms of reuse of such Open Access shall be governed by a Creative Commons licence – details of these licences and which Creative Commons licence will apply to this Work are set out in our licence referred to above.

Other than as permitted in any relevant BMJ Author's Self Archiving Policies, I confirm this Work has not been accepted for publication elsewhere, is not being considered for publication elsewhere and does not duplicate material already published. I confirm all authors consent to publication of this Work and authorise the granting of this licence.

For peer review only

Early childhood parent-reported speech problems in small and large for gestational age term- and preterm-born infants: A cohort study

Jee, Gabrielle[a], Kotecha, Sarah J.[b], Chakraborty, Mallinath [b,c], Kotecha, Sailesh[b,c], Odd, David[c,d]

[a]*Department of Paediatrics, University Hospital of Wales, Cardiff, UK*

[b]*Department of Child Health, School of Medicine, Cardiff University, Cardiff, UK*

[c]*Department of Neonatology, University Hospital of Wales, Cardiff, UK*

[d]*Department of Population Health, School of Medicine, Cardiff University, Cardiff, UK*

Corresponding author:

David Odd

Neuadd Meirionnydd, University Hospital of Wales, Heath Park, Cardiff, CF14 4YS

oddd@cardiff.ac.uk

+44(0)2920874000

Keywords:

Neonates, birthweight, neurodevelopmental outcome

Word count:

3657 words

For peer review only - http://bmjopen.bmj.com/site/about/guidelines.xhtml

ABSTRACT

Objective

1. To assess if preterm and term small (SGA) or large for gestational age (LGA) infants have more parent-reported speech problems in early childhood compared to infants with birthweights appropriate for gestational age (AGA).
2. To assess if preterm and term SGA and LGA infants have more parent-reported learning, behavioural, hearing, movement, and hand problems in early childhood compared to AGA infants.

Design

Cohort study.

Setting

Wales, United Kingdom.

Participants

7004 children with neurodevelopmental outcomes from the Respiratory and Neurological Outcomes of Children Born Preterm study (RANOPs) which enrolled 7129 children, born from 23 weeks of gestation onwards, to mothers aged 18 to 50 years of age were included in the analysis.

Outcome Measures

Parent-reported single-answer questionnaires were completed in 2013 to assess early childhood neurodevelopmental outcomes. The primary outcome was parent-reported speech problems in early childhood adjusted for clinical and demographic confounders

For peer review only

in SGA and LGA infants compared to AGA infants. Secondary outcomes measured were parent-reported early childhood learning, behavioural, hearing, movement, and hand problems.

Results

Median age at time of study was 5 years, range 2-10 years. Although the adjusted OR was 1.19 (0.92-1.55) for SGA infants and OR 1.11 (0.88-1.41) for LGA infants, this failed to reach statistical significance that these subgroups were more likely to have parent-reported speech problems in early childhood compared to AGA infants. This study also found parent-reported evidence suggestive of potential learning difficulties in early childhood (OR 1.51 (1.13-2.02)) and behavioural problems (OR 1.35 (1.01-1.79)) in SGA infants.

Conclusion

This study of 7004 infants in Wales suggest that infants born SGA or LGA likely do not have higher risks of parent-reported speech problems in early childhood compared to infants born AGA. To further ascertain this finding, studies with wider population coverage and longer-term follow up would be needed.

(287 words)

For peer review only

STRENGTHS AND LIMITATIONS

- This study has a relatively large sample size, although this represents around 26% of all eligible invitees to RANOPs (n=26722).
- Major domains of early childhood developmental outcomes were explored.
- Potentially using parent-reported questionnaires may introduce some degree of recall bias
- Like most cohort studies, missing data in those enrolled, particularly in confounders, was present.
- Data was collected between 2003 and 2011.

For peer review only

INTRODUCTION

Background

Birthweight is a complex product of intrinsic and extrinsic influences on the feto-maternal interaction and is an important predictor of perinatal morbidity and mortality.[1]–[3] Population studies have shown trends that infants are being born heavier. In England and Wales, there was an average increase of 40 grams over 26 years for all live births recorded between 1986 and 2012 with a 8-10% increased risk of being born with a high birthweight during this period.[4] Trends for heavier birthweights were also observed in other countries including Canada, USA and Sweden, and the cause for this shift is not understood.[4] This drives a need to better understand predictors of birthweight and its' association with longer-term outcomes.

Outcomes for certain subgroups such as prematurity and small for gestational age (SGA) have been well explored but remains unclear for large for gestational age (LGA) infants particularly mid- to long-term outcomes. LGA is associated with birth complications and indirect effects persisting into adulthood.[5] Around birth, larger term infants have increased risk of shoulder dystocia, meconium aspiration, lower 5-minute Apgar scores and death.[6], [7] Postnatally, they are more likely to have polycythemia, hypoglycaemia and respiratory distress syndrome; all conditions associated with poor long-term outcomes.[6]–[8] In preterm births, the prognosis for being LGA is unclear, with some literature suggesting an advantage; reporting lower perinatal mortality in preterm LGA infants, but with higher risks of early-onset sepsis and intraventricular haemorrhage.[9]

For peer review only - http://bmjopen.bmj.com/site/about/guidelines.xhtml

Overall, data on the mid- to long-term effects of being LGA across all gestational ages is lacking, and little is known about neurodevelopmental outcomes. A retrospective cohort study by Moore et.al found an increased risk of autism in term infants born SGA between 23 and 31 weeks, whereas being born large may have conferred some protective effect.[10] In view of speech being a dynamic product of higher function cognitive and sensorimotor feedback processes involving multiple cortical and subcortical areas for planning, selecting, sequencing and motor programming, it was selected as the primary outcome of interest. Due to the complex interaction between neurodevelopmental domains incuding speech, this study also evaluated learning, behavioural, hearing, movement, and hand problems.[11]

OBJECTIVES

1. To assess if preterm and term small (SGA) or large for gestational age (LGA) infants have more parent-reported speech problems in early childhood compared to infants with birthweights appropriate for gestational age (AGA).
2. To assess if preterm and term SGA and LGA infants have more parent-reported learning, behavioural, hearing, movement, and hand problems in early childhood compared to AGA infants.

METHODS

Study design

This study was conducted using data collected from the Respiratory and Neurological Outcomes of Children Born Preterm study (RANOPs), a cross-sectional population study conducted in Wales in 2013. This study recruited equal numbers of preterm (n=13361) and term-born children (n=13361) in years 2003, 2005, 2007, 2009 and

For peer review only - http://bmjopen.bmj.com/site/about/guidelines.xhtml

2011 to complete questionnaires on respiratory and neurodevelopmental outcomes if their child has ever had any active or resolved problems.[Supplement 1, 2]

Term infants were selected to be comparable with the preterm infants, for date of birth, sex, and locality. A total of 7129 responses were received including consent for data usage and access to health databases. Characteristics between those who enrolled, and those that didn't are shown in Supplement 3. Parents with preterm infants were more likely to respond to the questionnaire than those with term births ($p<0.001$) and responders were less likely (39.5% vs 53.8%) to live in the most deprived half of Wales. However, the proportions of males ($p=0.52$), and those with low, normal, or high birthweights for their gestation were similar ($p=0.61$).

Parent-reported answers on neurodevelopmental outcomes were collected in 2013 for all ages. For example, "Does your child have any problems with their speech?"; followed by a yes, or no, option. In the event neither option was selected, the response was recorded as unsure. Baseline demographics, including birth and maternal characteristics, were collected from national health databases.[11]–[13]

The primary neurodevelopmental outcome was parent-reported early childhood speech problems.[11] Secondary neurodevelopmental outcomes were parent-reported early childhood learning, behavioural, hearing, movement, and hand problems. For this study, all 'unsure' responses were re-coded as 'no' and included in the primary analysis.

For peer review only - http://bmjopen.bmj.com/site/about/guidelines.xhtml

Study Population

The eligible population were all children enrolled in RANOPs, born from 23 weeks of gestation to mothers from 18 to 50 years of age with available speech outcomes (n=7004). Exposure measures were gestational age at birth, and birthweight centile by category. Birthweight centiles were calculated for each sex and gestation (in weeks) using the LMS Growth program (Medical Research Council).[14] SGA was defined as <10th centile on the UK-WHO growth charts and >90th centile for LGA. These are values generally accepted across England and Wales, with Scotland utilising the 5th and 95th centiles.[15], [16] Gestational age was categorised (as per WHO definitions) as extremely preterm (<28 weeks), very preterm (28 to 31 weeks) and moderate to late preterm (32 to <37 weeks).[17] Post-maturity was defined as gestational age at birth greater than or equal to 42 weeks.[18] SGA and LGA infants were compared to appropriate for gestational age (AGA) infants across gestations.

Covariates

Covariates included neonatal and maternal influences known a priori to influence birthweight in-utero. Neonatal factors are singleton or multiple births, gestational age at birth, and sex. Maternal factors accounted for, included smoking during pregnancy, the Welsh Index of Multiple Deprivation (WIMD) score (a measurement of relative deprivation for small areas with scores of 1 to 1909, 1 being the most deprived), and their age.[19] These overlap with potential influences on the primary outcome, parent-reported speech problems in early childhood. While complex and multifactorial in potentially influencing the primary outcome, maternal socioeconomic status was accounted for using WIMD scores.[20] Predictors of low birthweight include low birthweight[21], multiple pregnancies[22], and fetal sex[23]. Mode of delivery was not assumed to impact birthweight and considered likely multifactorial due to perinatal

For peer review only - http://bmjopen.bmj.com/site/about/guidelines.xhtml

clinical practice surrounding estimated birthweight or centile, birth complications, and maternal preference.[15], [24], [25]. The determinants of high birthweight are however less clear.

Evidence suggests that active smoking is associated with increased risk of low birth weight and preterm birth, and prevalence of smoking is associated with socioeconomic status.[26], [27] There is also an increased risk of low birthweight with younger mothers, perhaps influenced by socioeconomic status. However, evidence surrounding birthweight and advanced maternal age is inconsistent, with some studies showing increased prevalence of infants born LGA, while others showing a U-shaped trend of low birthweight with increasing age.[26], [28]–[31]

Statistical Analysis

Duplicates and infants with missing exposure or primary outcome data were removed. Initially, neonatal, and maternal characteristics at birth for all infants were compared across their birthweight centile category. Comparisons were performed using the Kruskal-Wallis equality of populations ranks test for birthweight centile category, gestational age and WIMD score at birth. Sex, number of births, mode of delivery, and maternal smoking during pregnancy were compared using the Chi-Square test, and maternal age at delivery using the Analysis of Variance (ANOVA) test.

Next, the proportion of children with speech problems between birthweight centile categories was compared. Using a logistic regression model, the unadjusted and adjusted for potential confounders, odds ratios for speech problems, comparing SGA and LGA infants to those born AGA were derived. Stratifying by preterm and term

For peer review only - http://bmjopen.bmj.com/site/about/guidelines.xhtml

infants, and using a logistic regression model, the odds ratios for speech problems were also derived for SGA and LGA infants compared to AGA infants. In view of a single primary outcome, p-values corresponding to each statistical test was not corrected.

Six sensitivity analyses were performed. Firstly, we repeated the logistic regression using a random-effects model to cluster by week of gestational age, and secondly by the age of the child at time of the survey. We then repeated using the 5th and 95th centile cut-offs, and then again removing all responders where ‘unsure’ was coded for the primary outcome.[16] The main analysis was also repeated with an ordinal logistic regression analysis looking at the odds of an increasing number of reported neurodevelopmental disorders. Finally, the analysis was repeated using a missing data technique (Multiple Imputation with Chain Equations (details in Supplement 4)) to assess the impact of missing outcome and covariate data on the association seen.[17] Likelihood ratio tests were used to compare models. Analysis was performed using Stata SE 17 (Statacorp LLC).

Patient and Public Involvement

Patients or the public were not involved in the design, conduct, reporting, or dissemination plans of this research.

RESULTS

4284 preterm and 2865 term infants were enrolled in RANOPs (n=7149).[12] 20 duplicates were removed, leaving 7129 children. Those born to mothers younger than 18 or above 50 years of age were excluded (n=83), leaving 7046 eligible children. 8

For peer review only - http://bmjopen.bmj.com/site/about/guidelines.xhtml

infants had missing birthweight centiles, and a further 34 had missing data on the primary outcome, early childhood speech problems, leaving a study population of 7004 participants for the primary analysis (99.40% of the eligible responders).

Stratifying by birthweight centile categories of SGA, AGA and LGA infants, baseline neonatal and maternal characteristics of the study cohort are shown in Table 1. The median age at time of the survey was 4.08 to 5.17 (p=0.69). Besides the distribution of sex across birthweight centile categories, there were significant differences in other baseline neonatal and maternal characteristics analysed.

Table 1. Neonatal and maternal birth characteristics for all including preterm and term-born infants.

**Characteristics**		**Birthweight centile**			
	**n**	**SGA (<10th)**	**AGA (10-90th)**	**LGA (>90th)**	**P value**
Age at survey (median years)	7003	4.08 (2.67-7.67)	5.17 (2.75-7.67)	4.37 (2.75-7.67)	0.69
Neonatal Factors					
Male	7004	313 (48.68%)	2458 (45.50%)	450 (46.92%)	0.26
Singleton	7004	507 (78.85%)	4647 (86.02%)	904 (94.26%)	<0.001
Gestation (median weeks)	7004	35 (33-38)	36 (34-39)	36 (34-38)	<0.001
Preterm (< 37 weeks)	7004	458 (71.23%)	3105 (57.48%)	623 (64.96%)	<0.001
Post term (≥ 42 weeks)	7004	25 (3.89%)	99 (1.83%)	5 (0.52%)	<0.001
Mode of delivery	4091				<0.001
Unassisted vaginal delivery	2037	119 (32.16%)	1659 (52.33%)	259 (47.01%)	
Instrumental	406	27 (7.30%)	334 (10.54%)	45 (8.17%)	
Elective caesarean section	511	66 (17.84%)	349 (11.01%)	96 (17.42%)	
Emergency caesarean section	1137	158 (42.70%)	828 (26.12%)	151 (27.40%)	
Maternal Factors					
Age (mean years)*	6225	29.85 (6.00)	30.35 (5.66)	31.15 (5.37)	<0.001
WIMD score (median)†	6804	845 (428-1351)	985 (503-1447)	985 (524-1444)	<0.001
WIMD decile†	6804	5 (3-7)	6 (3-8)	6 (3-8)	<0.001
Maternal smoking‡	6725	133 (21.38%)	655 (12.65%)	75 (8.11%)	<0.001

Values are n (%), mean (SD) or median (IQR) as appropriate
Denominator between measures differs due to missing data
*Maternal age at the time of delivery
†Welsh Index of Multiple Deprivation/Deciles of WIMD (lower values reflecting more deprivation)
‡Maternal smoking during pregnancy

Distribution of early childhood speech problems in preterm and term infants by birthweight centile is as shown in Figure 1. Figure 2 depicts the number of

For peer review only - http://bmjopen.bmj.com/site/about/guidelines.xhtml

neurodevelopmental disorders stratified by gestation age at birth, birthweight centile category, and deprivation measures or WIMD category (quintile). Analysis of the distribution of the primary outcome, parent-reported speech problems showed a significant difference between different birthweight centile categories, p=0.05. There were significant differences between birthweight centile categories and the secondary outcomes, except for parent-reported hearing problems (p=0.59) [Table 2].

Table 2. Early childhood neurodevelopmental outcomes across all birthweight categories.

Outcome Measure	n	Birthweight centile			
		SGA (<10th) n (%)	AGA (10-90th) n (%)	LGA (>90th) n (%)	P value
Primary					
Speech problems	7004	93 (14.46)	603 (11.16)	112 (11.68)	0.05
Secondary					
Learning difficulties	6980	83 (12.95)	404 (7.50)	70 (7.34)	<0.001
Behavioural problems	6963	86 (13.46)	459 (8.54)	77 (8.11)	<0.001
Hearing problems	6985	49 (7.66)	371 (6.89)	73 (7.62)	0.59
Movement problems	6990	45 (7.01)	240 (4.45)	49 (5.12)	0.01
Hand problems	7000	40 (6.22)	206 (3.82)	36 (3.76)	0.01

Logistic regression adjusted for neonatal and maternal features of the primary outcome (parent-reported speech problems in early childhood) showed that SGA and LGA infants were not more likely than their AGA counterparts to have problems, OR 1.19 (0.92-1.55) and OR 1.11 (0.88-1.41) respectively. Analysis of parent-reported secondary outcomes found evidence that SGA infants have an increased risk of learning difficulties in early childhood compared to those born AGA, OR 1.51 (1.13-2.02). There was also some evidence that SGA infants were more likely to have early childhood behavioural problems, OR 1.35 (1.01-1.79). This study did not find that infants born SGA or LGA were more likely to have hearing, movement, or hand problems in comparison to AGA infants. [Table 3]

Table 3. Unadjusted and adjusted odds ratios for early childhood neurodevelopmental outcomes in SGA and LGA infants compared to AGA controls.

Outcome Measures	Unadjusted	Adjusted for neonatal features*	Adjusted for neonatal* and maternal features†

For peer review only - http://bmjopen.bmj.com/site/about/guidelines.xhtml

	n	Birthweight centile		n	Birthweight centile		n	Birthweight centile	
		SGA (<10th)	LGA (>90th)		SGA (<10th)	LGA (>90th)		SGA (<10th)	LGA (>90th)
Primary									
Speech problems	7004	1.34 (1.06-1.70)	1.05 (0.85-1.30)	7004	1.28 (1.01-1.63)	1.01 (0.82-1.26)	5935	1.19 (0.92-1.55)	1.11 (0.88-1.41)
Secondary									
Learning difficulties	6980	1.83 (1.42-2.36)	0.98 (0.75-1.27)	6980	1.71 (1.32-2.22)	0.92 (0.71-1.21)	5894	1.51 (1.13-2.02)	1.06 (0.79-1.41)
Behavioural problems	6963	1.67 (1.30-2.13)	0.95 (0.74-1.22)	6963	1.57 (1.22-2.02)	0.89 (0.69-1.15)	6963	1.35 (1.01-1.79)	1.19 (0.91-1.57)
Hearing problems	6985	1.12 (0.82-1.53)	1.12 (0.86-1.45)	6985	1.06 (0.77-1.45)	1.08 (0.83-1.40)	5900	1.03 (0.73-1.47)	1.16 (0.87-1.54)
Movement problems	6990	1.62 (1.16-2.25)	1.16 (0.84-1.59)	6990	1.39 (0.99-1.94)	1.08 (0.78-1.49)	5901	1.43 (0.98-2.09)	1.31 (0.92-1.84)
Hand problems	7000	1.67 (1.18-2.37)	0.98 (0.69-1.41)	7000	1.56 (1.09-2.23)	0.95 (0.66-1.36)	5910	1.36 (0.90-2.06)	1.11 (0.75-1.64)

*Adjusted for foetal sex, singleton or multiple birth, and gestational age at birth
†Adjusted for maternal age and WIMD score at time of birth, and maternal smoking status during pregnancy

Analysis was repeated using a random-effects regression model, stratified by preterm- or term-born, and the adjusted odds ratios did not demonstrate that preterm infants born SGA or LGA were more likely to have parent-reported speech-problems than infants born AGA. There was also no evidence of interaction between the exposures measured, ($p_{interaction}$=0.999). [Table 4]

Table 4. Unadjusted and adjusted odds ratios for primary neurodevelopmental outcome of early childhood speech problems stratified by preterm or term in SGA and LGA infants compared to AGA controls.

**Gestation**	**Birthweight category**	**Unadjusted (n = 7004)**		**Adjusted for neonatal features* (n = 7004)**		**Adjusted for neonatal* and maternal features† (n = 5935)**	
		OR (95% CI)	$P_{interaction}$	OR (95% CI)	$P_{interaction}$	OR (95% CI)	$P_{interaction}$
**Preterm (<37 weeks)**	SGA (<10th)	1.27 (0.98-1.66)	0.864	1.30 (0.99-1.71)	0.844	1.19 (0.88-1.61)	0.999
	LGA (>90th)	1.04 (0.81-1.33)		1.05 (0.82-1.35)		1.12 (0.85-1.47)	

For peer review only

**Term (≥37 weeks)**	SGA (<10th)	1.16 (0.69-1.96)		1.21 (0.71-2.05)		1.19 (0.69-2.07)	
	LGA (>90th)	0.92 (0.60-1.42)		0.91 (0.59-1.41)		1.10 (0.71-1.72)	

*Adjusted for foetal sex, singleton, or multiple birth
†Adjusted for maternal age and WIMD score at time of birth, and maternal smoking status during pregnancy

Multi-level regression model of SGA and LGA babies compared to those born AGA across each gestational age category (using child age as the clustering variable) also showed compatible results (SGA, OR 1.19 (0.92-1.55); LGA, OR 1.11 (0.87-1.41) with the main analysis. When ‘unsure’ responses excluded from analysis, logistic regression repeated showed no increased likelihood in primary neurodevelopmental outcome, parent-reported early childhood speech problems in babies born SGA, OR 1.20 (0.92-1.56) or LGA, OR 1.13 (0.90-1.44) compared to those born AGA. Repeat of the analysis using the 5th and 95th centiles as cut-offs for SGA showed that babies born SGA were not more likely than babies born AGA to have parent-reported early childhood speech problems, OR 1.35 (0.98-1.86).[16] There was also no evidence to suggest more parent-reported early childhood speech problems in babies born LGA, OR 1.11(0.84-1.48). Repeat of the main analysis examining the odds of an increasing number of reported developmental disorders, found results compatible with the primary analysis (SGA, OR 1.17 (0.96-1.43); LGA, OR 1.11 (0.93-1.32). Finally, repeating the main analysis using a multiple imputation model (n=7004) (SGA, OR 1.21 (0.95-1.54); LGA, OR 1.06 (0.85-1.32)) was also compatible, with no clear associations seen.

For peer review only

DISCUSSION

The literature supports that parents' perceptions on their child's development have shown significant consistency with standardised developmental objective measures in preterm- and term-born infants, and are increasingly used in developmental screening for at risk infants. [32]–[40] Example of such is the validated PARCA-R (Parent Report of Children's Abilities) for very-preterm infants at 2 years.[41] Although some studies have reported variation between parental estimates and objective measures of speech development, parent reported speech outcomes especially speech intelligibility still remains widely used in clinical practice.[42]–[44] While the absolute numbers reported here are large, they do only represent a relatively low proportion of those invited to enroll in the study; although they had similar low or high birthweigths compared to those who didn't enroll. However, they also appeared to come from less deprived areas than the wider population, and interpretation of our findings should consider this.

The production of speech is complex, relying on not only intact cognitive, motor and sensory functions but also complicated by hearing loss, particularly the child's age at time of hearing loss.[32], [45] Reassuringly, in this work we were unable to identify clear associations between SGA, or LGA infants and adverse parent-reported speech outcomes in early childhood; in either the unadjusted or adjusted models, as well as in the analysis of hearing problems. Also reassuringly, this study also did not find an increased risk of adverse parent-reported behavioural and hand problems in SGA and LGA infants, movement problems in SGA infants and learning difficulties in LGA infants. However, confidence intervals are relatively wide and important increases in morbidity cannot be excluded and more work with precise estimate may be warranted.

For peer review only

Evidence from this study suggest that SGA infants may have higher risks of parent-reported learning difficulties and behavioural problems in early childhood but not hearing, movement, or hand problems. A single-centre cohort study of term infants in Australia born to women presenting antenatally between 1981 and 1984 showed that children born SGA had significantly more learning difficulties when followed up at 14 years of age using a parent-reported survey and academic achievement test. This study reported a comparable 19.8% and 20.5% incidence for those who completed psychometric testing and behavioural questionnaires respectively. This study also demonstrated long-term attention difficulties in extremely SGA (3rd centile and below) term-born female adolescents.[46] Similar findings of poorer attention, executive function and memory were reported in a small cohort study of SGA preterm- and term-born young adults who underwent neuropsychological assessment.[47] Similarly, a cross-sectional study of 5181 childrens' behaviour, between ages 4 to 15 years in England, using a validated parent-reported questionnaire and stratifying for sociodemographic factors, suggested an association between birthweight and behavioural problems in children.[48][6]

We also saw that the proportion of all parent-reported early childhood disorders showed a strong relationship with gestational age at birth (seen in Figure 2). This appears consistent with the wider literature, [49]–[51] and this work was designed to adjusted, in part, for this by using birthweight centiles. Alternatively, this may in part reflect the complexities and co-dependency of many of these outcomes as gestational age lowers, and further work is currently underway to look at the phenotypes and interactions of challenges these infants demonstrate.

For peer review only

For peer review only - http://bmjopen.bmj.com/site/about/guidelines.xhtml

Analysis of baseline characteristics noted a significant difference in maternal age, and demographics at birth between birthweight centile categories. While this study was not designed to explore the relationship between maternal age and birthweight centile categories, previous studies have reported significant findings of higher maternal age in LGA infants.[28]–[31] In addition, the association between maternal smoking, deprivation, and birthweight centile categories was sobering, with 20% of SGA infants exposed to in-utero smoking. In this work, more than 1 in 5 mothers smoked during pregnancy, identifying an important public health focus on a modifiable risk factor for low birth weight to address,[26] with even first trimester cessation of smoking having substantial benefits.[26], [52], [53]

There was also a significant difference in the mode of delivery across birthweight centile categories likely due to clinical practice managing high-risk pregnancies[15], [24], [25]. This may include singleton or multiple pregnancies, gestational age and estimated birthweight or centile at time of birth. Sex was not associated with SGA or LGA, compatible with findings of a large cohort study in Netherlands.[23]

CONCLUSIONS

Findings from this work, on a subset of 7004 infants in Wales suggest that infants born small (SGA) or large for gestational age (LGA) may not have higher risks of speech problems in early childhood when compared to appropriate for gestational age (AGA) infants. While enrolment was achieved on only a subset of, less deprived infants, important differences may still exist, and we found that some infants being born SGA may have increased parent-reported learning difficulties and behavioural problems compared to AGA infants. Further longer-term studies on infants born SGA and LGA

For peer review only - http://bmjopen.bmj.com/site/about/guidelines.xhtml

would be of value to better understand the association of birthweight on neurodevelopment.

DECLARATIONS

Funding

No additional funding was received for this analysis. The original study, RANOPs was funded by Medical Research Council Experimental Medicine Challenge Grant (reg: MR/M022552/1), Mason Medical Research Foundation grant, and Children and Young Peoples Research Network Wales.

Competing interests

The authors have no relevant financial or non-financial interests to disclose.

Acknowledgements

We would like to thank all participants in this study.

Author contributions

Gabrielle Jee conceived and designed this work, wrote statistical analysis plan, cleaned, and analysed data, drafted first manuscript, revised draft manuscript and edited and updated final paper.

David Odd conceived and designed this work, wrote statistical analysis plan, analysed data and drafted first manuscript, revised draft manuscript and edited and updated final paper

For peer review only - http://bmjopen.bmj.com/site/about/guidelines.xhtml

Sarah J. Kotecha and Sailesh Kotecha conceived, designed, performed original RANOPs study including data collection, revised draft manuscript, and edited and updated final paper.

Mallinath Chakraborty revised the draft manuscript and edited and updated final paper.

Ethical approval

Ethical approval was sought at initiation of RANOPs and approved by South East Wales Research Ethics Committee (Research Ethics Committee 12/WA/0155 Project 91349). Parents provided written consent to participate.

Data sharing

All data must be held securely at Cardiff University as per ethical approval given for this research. Anonymous data will be available from the department of Child Health at Cardiff University to bona fide researchers provided that ethical approval is obtained from a research ethics committee in the UK for any suggested studies. Requests for data access should be sent to Sailesh Kotecha (Kotechas@cardiff.ac.uk).

For peer review only

For peer review only - http://bmjopen.bmj.com/site/about/guidelines.xhtml

REFERENCES

[1] G. Chiossi, C. Pedroza, M. M. Costantine, V. T. T. Truong, G. Gargano, and G. R. Saade, “Customized *vs* population-based growth charts to identify neonates at risk of adverse outcome: systematic review and Bayesian meta-analysis of observational studies,” *Ultrasound in Obstetrics & Gynecology*, vol. 50, no. 2, Aug. 2017, doi: 10.1002/uog.17381.

[2] J. A. Hutcheon, X. Zhang, R. W. Platt, S. Cnattingius, and M. S. Kramer, “The case against customised birthweight standards,” *Paediatr Perinat Epidemiol*, vol. 25, no. 1, Jan. 2011, doi: 10.1111/j.1365-3016.2010.01155.x.

[3] R. J. Baer *et al.*, “Population-based risks of mortality and preterm morbidity by gestational age and birth weight,” *Journal of Perinatology*, vol. 36, no. 11, Nov. 2016, doi: 10.1038/jp.2016.118.

[4] R. E. Ghosh, J. D. Berild, A. F. Sterrantino, M. B. Toledano, and A. L. Hansell, “Birth weight trends in England and Wales (1986–2012): babies are getting heavier,” *Arch Dis Child Fetal Neonatal Ed*, vol. 103, no. 3, pp. F264–F270, May 2018, doi: 10.1136/archdischild-2016-311790.

[5] V. Chiavaroli, J. G. B. Derraik, P. L. Hofman, and W. S. Cutfield, “Born Large for Gestational Age: Bigger Is Not Always Better,” *J Pediatr*, vol. 170, pp. 307–311, Mar. 2016, doi: 10.1016/j.jpeds.2015.11.043.

[6] S. L. Boulet, G. R. Alexander, H. M. Salihu, and M. Pass, “Macrosomic births in the united states: Determinants, outcomes, and proposed grades of risk,” *Am J Obstet Gynecol*, vol. 188, no. 5, pp. 1372–1378, May 2003, doi: 10.1067/mob.2003.302.

[7] A. Z. Khambalia, C. S. Algert, J. R. Bowen, R. J. Collie, and C. L. Roberts, “Long-term outcomes for large for gestational age infants born at term,” *J Paediatr Child Health*, vol. 53, no. 9, Sep. 2017, doi: 10.1111/jpc.13593.

[8] S. Dollberg, “Normoblasts in large for gestational age infants,” *Arch Dis Child Fetal Neonatal Ed*, vol. 83, no. 2, Sep. 2000, doi: 10.1136/fn.83.2.F148.

[9] N. S. Boghossian, M. Geraci, E. M. Edwards, and J. D. Horbar, “In-Hospital Outcomes in Large for Gestational Age Infants at 22-29 Weeks of Gestation,” *J Pediatr*, vol. 198, Jul. 2018, doi: 10.1016/j.jpeds.2018.02.042.

[10] G. S. Moore, A. W. Kneitel, C. K. Walker, W. M. Gilbert, and G. Xing, “Autism risk in small- and large-for-gestational-age infants,” *Am J Obstet Gynecol*, vol. 206, no. 4, pp. 314.e1-314.e9, Apr. 2012, doi: 10.1016/j.ajog.2012.01.044.

[11] M. O. Edwards, S. J. Kotecha, J. Lowe, L. Richards, W. J. Watkins, and S. Kotecha, “Early-term birth is a risk factor for wheezing in childhood: A cross-sectional population study,” *Journal of Allergy and Clinical Immunology*, vol. 136, no. 3, pp. 581-587.e2, Sep. 2015, doi: 10.1016/j.jaci.2015.05.005.

[12] M. O. Edwards, S. J. Kotecha, J. Lowe, L. Richards, W. J. Watkins, and S. Kotecha, “Management of Prematurity-Associated Wheeze and Its Association with Atopy,” *PLoS One*, vol. 11, no. 5, May 2016, doi: 10.1371/journal.pone.0155695.

[13] J. Lowe, S. J. Kotecha, W. J. Watkins, and S. Kotecha, “Effect of fetal and infant growth on respiratory symptoms in preterm-born children,” *Pediatr Pulmonol*, vol. 53, no. 2, pp. 189–196, Feb. 2018, doi: 10.1002/ppul.23920.

[14] H. Pan and T. Cole, “LMSgrowth, a Microsoft Excel add-in to access growth references based on the LMS method; Version 2.77.” 2012. [Online]. Available: http://wwwhealthforallchildrencouk/

For peer review only - http://bmjopen.bmj.com/site/about/guidelines.xhtml

[15] Royal College of Obstetricians & Gynaecologists, “The Investigation and Management of the Small-for-Gestational-Age Fetus. Green-top Guideline No.31. 2nd Edition,” Jan. 2014.
[16] Public Health Scotland, “Appropriate Birthweight for Gestational Age,” Nov. 2020. https://www.opendata.nhs.scot/hu/dataset/births-in-scottish-hospitals/resource/a5d4de3f-e340-455f-b4e4-e26321d09207 (accessed Oct. 29, 2021).
[17] World Health Organisation, “Preterm Birth,” Feb. 19, 2018. https://www.who.int/news-room/fact-sheets/detail/preterm-birth (accessed Oct. 29, 2019).
[18] The American College of Obstetricians and Gynecologists Committee on Obstetric Practice Society for Maternal-Fetal Medicine, “Committee Opinion No 579: Definition of Term Pregnancy,” *Obstetrics & Gynecology*, vol. 122, no. 5, Nov. 2013, doi: 10.1097/01.AOG.0000437385.88715.4a.
[19] Statistics for Wales, “Welsh Index of Multiple Deprivation,” 2019. https://statswales.gov.wales/Catalogue/Community-Safety-and-Social-Inclusion/Welsh-Index-of-Multiple-Deprivation (accessed Jan. 31, 2022).
[20] T. N. Basit, A. Hughes, Z. Iqbal, and J. Cooper, “The influence of socio-economic status and ethnicity on speech and language development,” *Int J Early Years Educ*, vol. 23, no. 1, pp. 115–133, Jan. 2015, doi: 10.1080/09669760.2014.973838.
[21] M. Mohsin, F. Wong, A. Bauman, and J. Bai, “Maternal and Neonatal Factors Influencing Premature Birth and Low Birth Weight in Australia,” *J Biosoc Sci*, vol. 35, no. 2, pp. 161–174, Apr. 2003, doi: 10.1017/S0021932003001615.
[22] M. Gielen *et al.*, “Secular trends in gestational age and birthweight in twins,” *Human Reproduction*, vol. 25, no. 9, pp. 2346–2353, Sep. 2010, doi: 10.1093/humrep/deq160.
[23] B. J. Voskamp *et al.*, “Association between fetal sex, birthweight percentile and adverse pregnancy outcome,” *Acta Obstet Gynecol Scand*, vol. 99, no. 1, pp. 48–58, Jan. 2020, doi: 10.1111/aogs.13709.
[24] Royal College of Obstetricians and Gynaecologists, “Shoulder Dystocia,” *Green-top Guideline No.42, 2nd Edition*, 2012. https://www.rcog.org.uk/globalassets/documents/guidelines/gtg_42.pdf (accessed Jan. 11, 2022).
[25] “Management of Breech Presentation,” *BJOG*, vol. 124, no. 7, pp. e151–e177, Jun. 2017, doi: 10.1111/1471-0528.14465.
[26] C. Johnson, S. Jones, and S. Paranjothy, “Low Birth Weight Review,” Jul. 2014, Accessed: Jan. 31, 2022. [Online]. Available: https://www2.nphs.wales.nhs.uk/ChildrenMatFamiliesDocs.nsf/($all)/E3F761EC6EFE646F80257D490044FBAE/$file/Low%20Birth%20Weight%20-%20technical%20paper%20v1.pdf?OpenElement
[27] F. McAndrew, J. Thompson, L. Fellows, A. Large, M. Speed, and M. Renfrew, “Infant Feeding Survey 2010,” 2010. Accessed: Jan. 31, 2022. [Online]. Available: https://sp.ukdataservice.ac.uk/doc/7281/mrdoc/pdf/7281_ifs-uk-2010_report.pdf
[28] A. Weissmann-Brenner *et al.*, “Maternal and neonatal outcomes of large for gestational age pregnancies,” *Acta Obstet Gynecol Scand*, vol. 91, no. 7, pp. 844–849, Jul. 2012, doi: 10.1111/j.1600-0412.2012.01412.x.
[29] W. Siggelkow, D. Boehm, C. Skala, M. Grosslercher, M. Schmidt, and H. Koelbl, “The influence of macrosomia on the duration of labor, the mode of

For peer review only

delivery and intrapartum complications," *Arch Gynecol Obstet*, vol. 278, no. 6, pp. 547–553, Dec. 2008, doi: 10.1007/s00404-008-0630-7.
[30] Y. Akin, S. Cömert, C. Turan, A. Piçak, T. Ağzikuru, and B. Telatar, "Macrosomic newborns: a 3-year review.," *Turk J Pediatr*, vol. 52, no. 4, pp. 378–83.
[31] R. Bakker, E. Steegers, A. Biharie, J. Mackenbach, A. Hofman, and V. Jaddoe, "Explaining differences in birth outcomes in relation to maternal age: the Generation R Study," *BJOG*, vol. 118, no. 4, pp. 500–509, Mar. 2011, doi: 10.1111/j.1471-0528.2010.02823.x.
[32] N. D. Emerson, H. E. R. Morrell, and C. Neece, "Predictors of Age of Diagnosis for Children with Autism Spectrum Disorder: The Role of a Consistent Source of Medical Care, Race, and Condition Severity," *J Autism Dev Disord*, vol. 46, no. 1, pp. 127–138, Jan. 2016, doi: 10.1007/s10803-015-2555-x.
[33] M. J. Vincer, H. Cake, M. Graven, L. Dodds, S. McHugh, and T. Fraboni, "A Population-Based Study to Determine the Performance of the Cognitive Adaptive Test/Clinical Linguistic and Auditory Milestone Scale to Predict the Mental Developmental Index at 18 Months on the Bayley Scales of Infant Development-II in Very Preterm Infants," *Pediatrics*, vol. 116, no. 6, pp. e864–e867, Dec. 2005, doi: 10.1542/peds.2005-0447.
[34] H.-F. Liao, T.-M. Wang, G. Yao, and W.-T. Lee, "Concurrent validity of the Comprehensive Developmental Inventory for Infants and Toddlers with the Bayley Scales of Infant Development-II in preterm infants.," *J Formos Med Assoc*, vol. 104, no. 10, pp. 731–7, Oct. 2005.
[35] C. Skellern, Y. Rogers, and M. O'Callaghan, "A parent-completed developmental questionnaire: Follow up of ex-premature infants," *J Paediatr Child Health*, vol. 37, no. 2, pp. 125–129, Apr. 2001, doi: 10.1046/j.1440-1754.2001.00604.x.
[36] K. B. Doig, M. M. Macias, C. F. Saylor, J. R. Craver, and P. E. Ingram, "The Child Development Inventory: A developmental outcome measure for follow-up of the high-risk infant," *J Pediatr*, vol. 135, no. 3, pp. 358–362, Sep. 1999, doi: 10.1016/S0022-3476(99)70134-4.
[37] J. Squires, D. Bricker, and L. Potter, "Revision of a Parent-Completed Developmental Screening Tool: Ages and Stages Questionnaires," *J Pediatr Psychol*, vol. 22, no. 3, pp. 313–328, 1997, doi: 10.1093/jpepsy/22.3.313.
[38] R. Bortolus, F. Parazzini, D. Trevisanuto, S. Cipriani, P. Ferrarese, and V. Zanardo, "Developmental assessment of preterm and term children at 18 months: reproducibility and validity of a postal questionnaire to parents," *Acta Paediatr*, vol. 91, no. 10, pp. 1101–1107, Oct. 2002, doi: 10.1080/080352502760311610.
[39] K. Sonnander, "PARENTAL DEVELOPMENTAL ASSESSMENT OF 18-MONTH-OLD CHILDREN: RELIABILITY AND PREDICTIVE VALUE," *Dev Med Child Neurol*, vol. 29, no. 3, pp. 351–362, Nov. 2008, doi: 10.1111/j.1469-8749.1987.tb02489.x.
[40] E. Prochazkova, V. Brichacek, J. Dittrichova, Sobotkova D, and J. Vondracek, "Parents' concerns regarding infant development: Kent Infant Development (KID) Scale," *Stud Psychol (Bratisl)*, vol. 39, pp. 263–265, 1997.
[41] A. J. Martin *et al.*, "Performance of the Parent Report of Children's Abilities-Revised (PARCA-R) versus the Bayley Scales of Infant Development III," *Arch*

For peer review only

Dis Child, vol. 98, no. 12, pp. 955–958, Dec. 2013, doi: 10.1136/archdischild-2012-303288.

[42] C. E. Venker, E. Haebig, J. Edwards, J. R. Saffran, and S. Ellis Weismer, "Brief Report: Early Lexical Comprehension in Young Children with ASD: Comparing Eye-Gaze Methodology and Parent Report," *J Autism Dev Disord*, vol. 46, no. 6, pp. 2260–2266, Jun. 2016, doi: 10.1007/s10803-016-2747-z.

[43] A. Nordahl-Hansen, A. Kaale, and S. E. Ulvund, "Language assessment in children with autism spectrum disorder: Concurrent validity between report-based assessments and direct tests," *Res Autism Spectr Disord*, vol. 8, no. 9, pp. 1100–1106, Sep. 2014, doi: 10.1016/j.rasd.2014.05.017.

[44] A. Sakash, T. Mahr, and K. C. Hustad, "Validity of Parent Ratings of Speech Intelligibility for Children with Cerebral Palsy," *Dev Neurorehabil*, vol. 24, no. 2, pp. 98–106, Feb. 2021, doi: 10.1080/17518423.2020.1830447.

[45] P. Tremblay, I. Deschamps, and V. L. Gracco, "Neurobiology of Speech Production," in *Neurobiology of Language*, Elsevier, 2016, pp. 741–750. doi: 10.1016/B978-0-12-407794-2.00059-6.

[46] M. J. O'Keeffe, M. O'Callaghan, G. M. Williams, J. M. Najman, and W. Bor, "Learning, Cognitive, and Attentional Problems in Adolescents Born Small for Gestational Age," *Pediatrics*, vol. 112, no. 2, pp. 301–307, Aug. 2003, doi: 10.1542/peds.112.2.301.

[47] H. F. Østgård *et al.*, "Neuropsychological Deficits in Young Adults Born Small-for-Gestational Age (SGA) at Term," *Journal of the International Neuropsychological Society*, vol. 20, no. 3, pp. 313–323, Mar. 2014, doi: 10.1017/S1355617714000034.

[48] Y. J. Kelly, J. Y. Nazroo, A. McMunn, R. Boreham, and M. Marmot, "Birthweight and behavioural problems in children: a modifiable effect?," *Int J Epidemiol*, vol. 30, no. 1, pp. 88–94, Feb. 2001, doi: 10.1093/ije/30.1.88.

[49] E. Himpens, C. van den Broeck, A. Oostra, P. Calders, and P. Vanhaesebrouck, "Prevalence, type, distribution, and severity of cerebral palsy in relation to gestational age: a meta-analytic review," *Dev Med Child Neurol*, vol. 50, no. 5, pp. 334–340, May 2008, doi: 10.1111/j.1469-8749.2008.02047.x.

[50] A. Pascal, P. Govaert, A. Oostra, G. Naulaers, E. Ortibus, and C. van den Broeck, "Neurodevelopmental outcome in very preterm and very-low-birthweight infants born over the past decade: a meta-analytic review," *Dev Med Child Neurol*, vol. 60, no. 4, pp. 342–355, Apr. 2018, doi: 10.1111/dmcn.13675.

[51] D. Moster, R. T. Lie, and T. Markestad, "Long-Term Medical and Social Consequences of Preterm Birth," *New England Journal of Medicine*, vol. 359, no. 3, pp. 262–273, Jul. 2008, doi: 10.1056/NEJMoa0706475.

[52] V. W. v. Jaddoe *et al.*, "Active and passive maternal smoking during pregnancy and the risks of low birthweight and preterm birth: the Generation R Study," *Paediatr Perinat Epidemiol*, vol. 22, no. 2, pp. 162–171, Mar. 2008, doi: 10.1111/j.1365-3016.2007.00916.x.

[53] K. Raatikainen, P. Huurinainen, and S. Heinonen, "Smoking in early gestation or through pregnancy: A decision crucial to pregnancy outcome," *Prev Med (Baltim)*, vol. 44, no. 1, pp. 59–63, Jan. 2007, doi: 10.1016/j.ypmed.2006.07.017.

For peer review only - http://bmjopen.bmj.com/site/about/guidelines.xhtml

For peer review only

For peer review only - http://bmjopen.bmj.com/site/about/guidelines.xhtml

Figure 1. Proportion of preterm, and term infants with parent-reported speech problems in early childhood by birthweight centile (Gaussian smoothed).

Figure 2. Proportion of neurodevelopmental disorders stratified by gestation age at birth, birthweight centile category, and WIMD category (quintile).

For peer review only

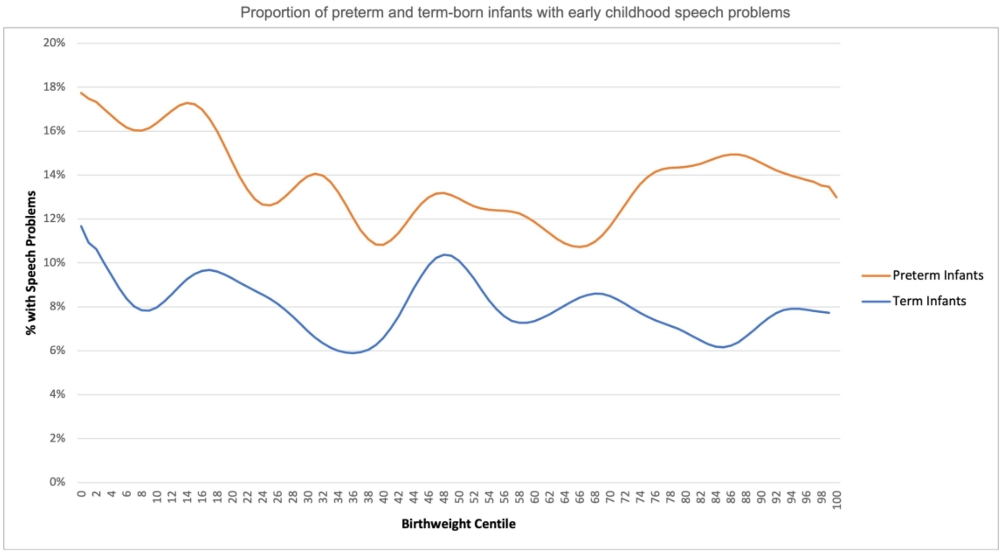

Figure 1. Proportion of preterm, and term infants with parent-reported speech problems in early childhood by birthweight centile (Gaussian smoothed).

323x179mm (300 x 300 DPI)

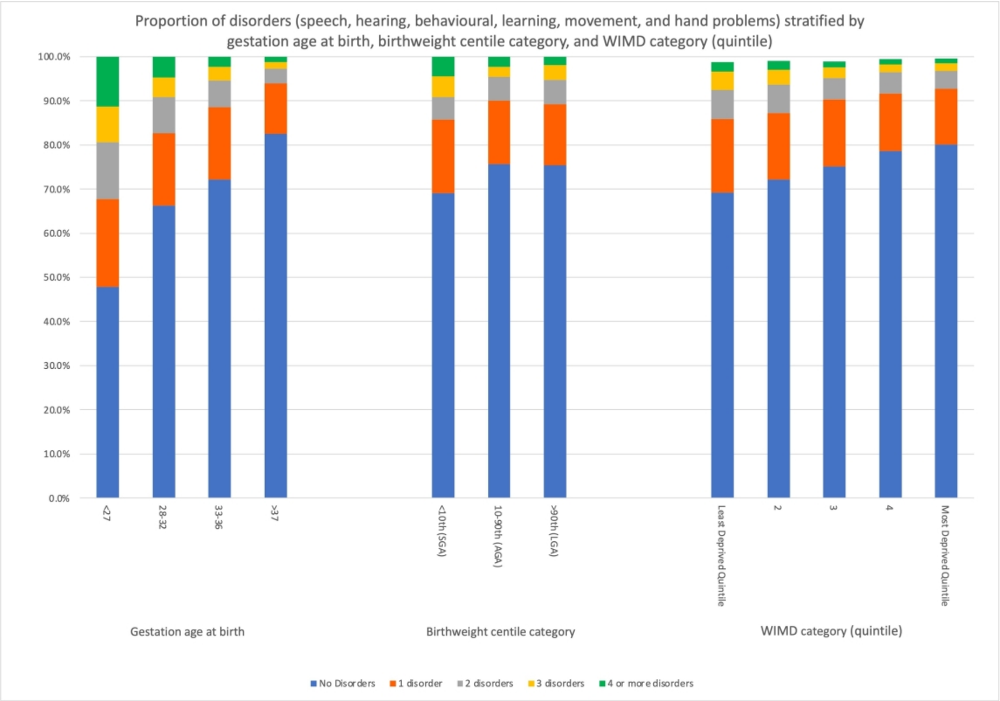

Figure 2. Proportion of neurodevelopmental disorders stratified by gestation age at birth, birthweight centile category, and WIMD category (quintile).

272x190mm (300 x 300 DPI)

Ethics protocol Version 1.1 5th November 2012

Respiratory and neurological outcomes in children born preterm study (RANOPs)

Research team:

Surname	First name	Title	Post held
Edwards	Martin	Dr	Clinical Research Fellow Cardiff University
Kotecha	Sailesh	Professor	Professor of Child Health (CI for study) Cardiff University
Dunstan	Frank	Professor	Professor of Medical Statistics Cardiff University
Henderson	John	Professor	Professor of Paediatric Respiratory Medicine Bristol University
Watkins	John	Dr	Medical Statistician Cardiff University
John	Gareth	Mr	Improvement and Innovations Manager NWIS
Richards	Louise	Mrs	Information Technology Support NWIS
Morris	Geraint	Dr	Consultant Paediatrician *(Local PI)* Abertawe Bro Morgannwg University Health Board
Papworth	Sue	Dr	Consultant Neonatologist *(Local PI)* Aneurin Bevan Health Board
Stutchfield	Peter	Dr	Consultant Paediatrician *(Local PI)* Betsi Cadwaladr University Health Board
Al-Muzaffar	Iyad	Dr	Consultant Neonatal Paediatrician *(Local PI)* Cwm Taf Health Board
Pitchaikani	Prem	Dr	Consultant Paediatrician *(Local PI)* Hywel Dda Health Board
Prosser	Ingrid	Dr	Consultant Community Paediatrician *(Local PI)* Powys Teaching Health Board

1. *Background:*

Babies born late preterm, between 33 and 36 weeks gestation (6-7% of all UK births), have a greater risk of developing breathing problems than previously appreciated. Our research group has recently shown that late preterm born 8-9 year olds have worse lung function than similarly aged children born at term but their lung function improves by 14-17 years of age (http://www.bbc.co.uk/news/uk-wales-south-east-wales-15071922).[1] In 2009 in England and Wales there were 706,248 live-births and over 42,000 were born late preterm.[2, 3] This group of children born late preterm tends to be treated the same as children born full term however there may be a need to monitor this group of children more closely.

Definition of terms:

Gestational groups - ≤32 weeks gestation = extremely preterm
- 33-36 weeks gestation = late preterm
- ≥37 weeks gestation = term

1.1 Research Question

We hypothesise that children born extremely preterm (23-32 weeks gestation) and late preterm (33 to 36 weeks gestation):

(a) have increased respiratory symptoms and disease,
(b) have increased neurodevelopmental problems,
(c) have increased health care utilisation, mainly due to respiratory morbidity

in infancy, in the pre-school and early school years, when compared to age-matched term-born children (control group).

For peer review only - http://bmjopen.bmj.com/site/about/guidelines.xhtml

1.2 Methods

Study Design: The morbidity and health utilisation of late preterm infants

In Wales, the average number of live-born deliveries over the last 5 years is 34,464 per annum.[2] Approximately 2,000 each year are born prematurely with 500 born extremely preterm and 1,500 late preterm.[2]

AWPS data: Total number of survivors at 1 year of age per year of birth and gestational age in Wales						
Year of birth	2003	2005	2007	2009	2010	2011
Gestational age						
23-32 weeks	436	507	503	471	513	492
33-36 weeks	1781	1719	1976	1980	1926	1913
37-43 weeks	26448	30063	31545	31933	33277	32819
Other/unknown	2303	226	279	323	171	334
Total	30968	32515	34303	34707	35887	35558

For late preterm infants, we will address:

(a) respiratory morbidity,

(b) neurodevelopmental problems and

(c) health care utilisation, especially due to respiratory reasons, via a questionnaire study and also the well-linked health databases in Wales with the collaboration of local neonatologists.

The data will be compared to term born and extremely preterm-born groups. Our team, including Professor John Henderson as well as Professor Frank Dunstan, has great expertise in questionnaire studies assessing health of children and adults.[4, 5] A modified ISAAC questionnaire focussing on respiratory symptoms (rather than allergy) will be used to assess respiratory health including wheezing, drug usage and physical activity as well as visits to hospitals and general practitioners in school-aged children[6] and a validated questionnaire by Powell et al will be used for preschool children .[7, 8] The questionnaires will be used to assess respiratory health and for any neurodevelopmental difficulties in a cross-sectional survey of children aged 1, 2, 3, 5, 7 and 9 years. The questionnaires will allow us to assess the impact of late preterm birth on respiratory outcomes, developmental delay and health service utilisation. The questionnaires will be age-appropriate (one for <5 years of age and another for 5-9 years of age). The two age-appropriate questionnaires are based on previous extensively validated questionnaires for both respiratory symptoms and neurodevelopmental outcomes in both preschool and school-aged children.[6-11]

We shall send 2,000 questionnaires with an invitation letter and parent information sheet for each age-group for the preterm groups (including 1,500 late preterm- and 500 extremely preterm-born children) together with a similar number for age-matched term controls. The matching of the term controls will be based on sex, place of birth and chronological age (DOB), although in the analysis of the data, the corrected postnatal age (this is the age a premature baby would be if born at term) of the preterm infants will be taken into account as a potential confounding factor. The total of 24,000 questionnaire packs will be sent via the NHS Wales Informatics Service (NWIS), which have expertise for identifying subjects from the NHS administrative register and then providing DST, the mailing house with a database to send out the questionnaires. Furthermore, children who have moved from the area or have died are identified and excluded, by using data from the All Wales Perinatal Survey (AWPS) which is directed by Professor Sailesh Kotecha with Professor Frank Dunstan a member of the steering committee.[2] AWPS maintains data on children who die before their first birthday, and this data will be used along with the Welsh Demographics Service, which also holds information on children who have passed away.

For peer review only - http://bmjopen.bmj.com/site/about/guidelines.xhtml

Prior to any reminder packs being sent out to the families, NWIS will be up-dated of all families who have returned a questionnaire pack. NWIS will then up-date the contact data base and also repeat the safe guard of who has died, and then send the new data base of contact information to DST, who will send out the reminder packs (1st after 1 month from the initial mailing date and 2nd after 3-4 months).

Secondly, using linked databases (including the Patient Episode Database for Wales, PEDW and the National Community Child Health Database, NCCHD and the Welsh Demographics Service, WDS) for hospital episodes, we will identify the general health service usage of these children. For those children with a returned, signed (consented) questionnaire we will be able to link the hospital data to their completed questionnaire to allow for more accurate analysis. The information gathered from PEDW, NCCHD and WDS will also contain data on all children less than 10 years of age in Wales (anonymised) and this will allow us to determine the representativeness of the sample returning questionnaires. The social status of a family will be determined by location using the welsh index for multiple deprivation child index score 2011 (taken from WDS).

The linked cross sectional data at 5 age points will be summarised in terms of rates of wheezing, rates of hospital admissions, etc. The exploration of the data will be hypothesis led to investigate if being born late preterm has an effect on the health outcome of children when compared to term born controls. The health outcomes will include hospital admissions, visits to GP, wheezing episodes, use of prescribed medication, doctor diagnosed conditions, long term disability and activity levels. The formal analysis will use general linear models to compare rates between different gestational age groups adjusting for confounders such as social class, maternal smoking during pregnancy, birth weight, gender etc.

Sample size: For a question with binary response (e.g. symptom present or absent), assuming a 50-60% response rate to the questionnaire, we will have 95% power for identifying a difference between the preterm- and term-born children if the true symptom rate is 15% in preterm and 10% in term children. We have estimated the response rates based on previous similar studies in Wales.[4, 12]

2. NWIS collaboration

We have already liaised with NWIS and shall ask their team to identify children born between 1/01/2003 to 31/12/2011. NWIS will identify all preterm infants (<37 weeks of gestation at birth) for each age group (1,2,3,5,7 & 9 years of age) using the national database (NCCHD and WDS). NWIS will then select the term controls by identifying gender matched children born on the same day and in the same area (hospital or midwifery led unit) as each preterm infant. So there will be approximately 2,000 term controls for each age group. For each age (1,2,3,5,7 and 9 years of age) there will be three groups; children born at full term (37-43 weeks of gestation), children born late preterm (33-36 weeks of gestation) and children born extremely preterm (≤32 weeks of gestation). Each child will be assigned a study number based on their DOB/gestational age/a unique reference number.

The information obtained from the health databases (WDS, NCCHD and PEDW) for each child will include the child's name, current address, gestational age, DOB, birth weight, breast feeding at birth and at 8 weeks of age, welsh index deprivation score, hospital admissions of the last year and discharge diagnosis. The postcode for the current address will be used to identify the local health board (so that the appropriate local PI is identified on the invitation letter being sent to the family). This information will need to be checked to remove any children that have died via AWPS and the WDS database. This will involve AWPS sending data to NWIS with the name, DOB and address of each child that has passed away over the last 10 years (1/1/03 to 31/12/12). ***None of this data will be made available to the research team.*** NWIS will supply DST, the mailing house with a study

For peer review only - http://bmjopen.bmj.com/site/about/guidelines.xhtml

database split into two sections (> 5 years of age and > 5 years of age). The database will contain the study ID number, name, address and local health board of each study participant. Data will be transferred between NWIS and DST via File Transfer Protocol Secure (FTPS).

DST will divide each data set into 7 sections according to the local health board in which the child resides. DST will then print the individual questionnaires (including study ID numbers), invitation letters including the family address and signed with the appropriate LHB with details of the local PI, and information sheets and place these in envelopes with windows that allow the letter to show the family address, and these packs will then be sent by royal mail to the families. Stamped addressed envelopes (to our research department) will be included within the study pack. DST also provide a further data health check, informing us of any duplicates and of families who may have moved, thus avoiding unnecessary/inappropriate mailing. ***No personal data will be released by NWIS to the research team at this stage.***

NWIS will send study ID numbers with the child's gestational age to the research team, so that when questionnaires are returned the correct gestational age for the child can be confirmed. Families will be given 4-8 weeks to return the questionnaires. A list of families who have returned questionnaires (by study ID numbers) will be sent to NWIS and they will send further information on each child that has a signed (consented) questionnaires, to include birth weight, antenatal details, hospital of birth, hospital admissions and discharge diagnosis over the last 12 months.

NWIS, following a re-check of the health data base for any deaths, will create a list of any families that have not returned a questionnaire. This up-dated study database will be given to DST and the first reminder pack will be sent to these families at 1 month after the initial pack was sent out. If the response rate remains low then this process will be repeated with a second remainder pack being sent after 3-4 months, when the research team will give NWIS a list of all families who have returned questionnaires and NWIS will collate an up-dated list, having removed any families where a child has died in the interim period since last checking. This up-dated study database will be given to DST and the second reminder pack will be sent to those families who have not yet responded.

Questionnaires will be returned to the Department of Child Health at Cardiff University and processed by automated scanners and specialised software (Remark Office OMR 8). The questionnaires will be coded by the software, which also identifies any problems such as missing information. Any problems detected by the software will be reviewed by the research team. This will form part of the data quality control checks. Anonymised data will be available for the whole cohort to allow us to determine the representativeness of the responders. The returned questionnaires will be processed by Dr Martin Edwards and all data stored securely in the research office at the Department of Child Health based at the University Hospital of Wales, Heath Park, Cardiff. By returning the completed questionnaires it will be assumed that the family is consenting to take part in the study. If the questionnaire form is signed the family will be consenting to be contacted with regards to clarifying any issues with the returned questionnaire; contacted in the future to take part in further research in this area; and for access to patient identifiable information in the health databases to link hospital admission and GP records information with that gathered from the questionnaires. It is clearly stated in the information leaflet what will happen with the data collected.

3. Ethical approval

The project has ethical and R&D approval from within Wales. The global governance check reference is **IRAS 91349** and the Ethics reference is **12/WA/0155.** The sponsor for the project is Cardiff University (**SPON1038-11**).

For peer review only - http://bmjopen.bmj.com/site/about/guidelines.xhtml

4. Timescale

We aim to mail the questionnaires between January and February 2013. We shall allow the families 6 – 8 weeks to respond initially and send a second mailing of questionnaires between April and May 2013 and if necessary a third mailing of questionnaires in between July and August 2013, dependent upon the response rate. Our expectation is to reach a 50 – 60% response rate as has been achieved in many recent questionnaire studies.[4, 12-14]

5. Resources

The study is currently funded by Departmental funds and research grant funding is being sought from several charities that support medical research – decision expected Jan/Feb 2013.

6. Documents

i. Letter of invitation to parents – 1 side of A4 paper
ii. Questionnaire for preschool children (<5 years old) – 4 sides of A4 paper
iii. Questionnaire for school aged children (5-9 years old) – 4 sides of A4 paper
iv. Information sheet for families – 2 sides of A4 paper
v. Flow diagram of research protocol

7. References

1. Kotecha, S.J., et al., *Effect of late preterm birth on longitudinal lung spirometry in school age children and adolescents.* Thorax.2012;67:54-61.
2. Kotecha S, et al. All Wales Perinatal Survey – Annual Report 2009. Cardiff University 2011.
3. Office of National Statistics (2009).
4. Burr ML, Wat D, Evans C, Dunstan FD, Doull IJ; BTS Research Committee. Asthma prevalence in 1973, 1988 and 2003. Thorax. 2006;61:296-9
5. Thomas H, et al. Mental health and quality of residential environment. Br J Psychiatry. 2007;191:500-5
6. Asher MI, et al. International study of asthma and allergies in childhood (ISAAC): Rationale and methods. Eur Respir J 1995;8:483-491.
7. Powell CV, McNamara P, Solis A, Shaw NJ. A parent completed questionnaire to describe the patterns of wheezing and other respiratory symptoms in infants and preschool children. *Archives of Disease in Childhood* 2002;87(5):376-9.
8. Trinick, R., K.W. Southern, and P.S. McNamara, *Assessing the Liverpool Respiratory Symptom Questionnaire in children with cystic fibrosis.* The European respiratory journal : official journal of the European Society for Clinical Respiratory Physiology, 2012;39(4):899-905.
9. Greenough A, Giffin FJ, Yuksel B. Respiratory morbidity in preschool children born prematurely. Relationship to adverse neonatal events. *Acta Paediatr* 1996;85(7):772-7.
10. Greenough A, Limb E, Marston L, Marlow N, Calvert S, Peacock J. Risk factors for respiratory morbidity in infancy after very premature birth. *Arch Dis Child Fetal Neonatal Ed* 2005;90(4):F320-3.
11. Jones H, Guildea Z, Stewart, J & Cartlidge, P.The health status questionnaire: Achieving concordance with published disability criteria. Archives of Disease in Childhood, 2002. **86**(1): p. 15-20.
12. Fox, R. and S. Minchom, *Parental experiences of the newborn hearing screening programme in Wales: a postal questionnaire survey.* Health expectations : an international journal of public participation in health care and health policy, 2008;11(4):376-83.
13. Leonardi, N.A., et al., *Validation of the Asthma Predictive Index and comparison with simpler clinical prediction rules.* The Journal of allergy and clinical immunology, 2011; 127(6):1466-72 e6.
14. Turner, S., et al., *First- and second-trimester fetal size and asthma outcomes at age 10 years.* American Journal of Respiratory and Critical Care Medicine, 2011;184(4);407-13.

For peer review only - http://bmjopen.bmj.com/site/about/guidelines.xhtml

Using a **PENCIL** fill in the circles like this

○ → ●

DO NOT tick or cross the circles.

An eraser can be used to rectify mistakes.

Study No: **12351**

Health questionnaire for preschool children (<5 years old)

Today's date ____________________2012

Person completing the questionnaire:

Mother	○
Father	○
Other	○

Date of birth of child: ______/_________/________

1. Has your child ever had wheezing or whistling in their chest at any time in their life?

Yes ○ No ○ Unsure ○

IF YOU HAVE ANSWERED "NO" PLEASE GO TO QUESTION 4.

2. In the last three months , during the day time (i.e. when awake) does your child:

	Not at all	a few days	some days	most days	every day
a. Wheeze:	○	○	○	○	○
b. Cough:	○	○	○	○	○
c. Short of breath:	○	○	○	○	○
d. Rattly chest:	○	○	○	○	○
e. Snuffly:	○	○	○	○	○

3. In the last three months , during the night time (i.e. when asleep) does your child:

	Not at all	a few nights	some nights	most nights	every night
a. Wheeze:	○	○	○	○	○
b. Cough:	○	○	○	○	○
c. Short of breath:	○	○	○	○	○
d. Rattly chest:	○	○	○	○	○
e. Snore:	○	○	○	○	○

4. How many colds has your child had in the last three months?

None ○ 1 to 3 ○ more than 3 ○ always has a cold ○

5. When my child has had a cold in the last three months, s/he has a:

	Not at all	a few colds	some colds	most colds	every cold
a. Wheeze:	○	○	○	○	○
b. Cough:	○	○	○	○	○
c. Short of breath:	○	○	○	○	○
d. Rattly chest:	○	○	○	○	○

For peer review only

For peer review only - http://bmjopen.bmj.com/site/about/guidelines.xhtml

Study No: **12351**

e. Snuffly:	○	○	○	○	○

6. When my child does NOT have a cold, in the last three months, s/he has a:

	Not at all	a few days	some days	most days	every day
a. Wheeze:	○	○	○	○	○
b. Cough:	○	○	○	○	○
c. Short of breath:	○	○	○	○	○
d. Rattly chest:	○	○	○	○	○
e. Snuffly:	○	○	○	○	○

7. When my child has been more active (e.g. crawling, walking or when excited), in the last three months, s/he has a:

	Not at all	a few days	some days	most days	every day
a. Wheeze:	○	○	○	○	○
b. Cough:	○	○	○	○	○
c. Short of breath:	○	○	○	○	○
d. Rattly chest:	○	○	○	○	○

8. Has your child ever been diagnosed with asthma by a doctor?

Yes ○ No ○

9. Is there a family history of

	Yes	No
a. Asthma	○	○
b. Eczema	○	○
c. Hayfever	○	○
d. Allergies	○	○

For peer review only

10. In the last 12 months, has your child ever used any regular asthma inhalers (pumps) or medicines?

Yes ○ No ○

If yes, please provide the name (or colour of the pump) with details of how often used:

____

____

11. In the last 12 months, has your child had any chest infections?

Yes ○ No ○

12. In the last 12 months, how many chest infections has your child had?

For peer review only - http://bmjopen.bmj.com/site/about/guidelines.xhtml

Study No: 12351

None ◯ 1 to 3 ◯ 4 to 10 ◯ more than 10 ◯

13. In the last 12 months, how many courses of antibiotics has your child had?
None ◯ 1 to 3 ◯ 4 to 10 ◯ more than 10 ◯

14. In the last 12 months, how many admissions (overnight or longer) has your child had to hospital for breathing problems?
None ◯ 1 to 3 ◯ 4 to 10 ◯ more than 10 ◯

14a. Does the child's mother smoke cigarettes? Yes ◯ No ◯

If yes, how many per day? 1 to 10 ◯ 11 to 20 ◯ more than 20 ◯

If the child's mother smokes did she smoke during the pregnancy? Yes ◯ No ◯

14b. Does the child's father smoke cigarettes? Yes ◯ No ◯

If yes, how many per day? 1 to 10 ◯ 11 to 20 ◯ more than 20 ◯

15. Do any other household members smoke cigarettes? Yes ◯ No ◯

If yes, how many per day for the whole household? 1 to 10 ◯
11 to 20 ◯
more than 20 ◯

(please add up all the cigarettes which are smoked by everyone living in the same household including the mother).

16. a . Has your child ever been diagnosed with any breathing problems (e.g. asthma, CF, TB etc.)?
Yes ◯ No ◯
If yes, please provide some details:
____
____

16. b . Has your child ever been diagnosed with any other conditions (e.g. diabetes, epilepsy etc.)?
Yes ◯ No ◯
If yes, please provide some details:
____
____

17. Is your child on any medication (please list all medicines your child is being given)?
Yes ◯ No ◯
If yes, please provide some details:
____
____

18. Does your child take part in any physical activity every week such as dancing, swimming, cycling ?
Yes ◯ No ◯
If yes, please provide some details of how often and for how long:

For peer review only - http://bmjopen.bmj.com/site/about/guidelines.xhtml

For peer review only

Study No: 12351

19. Does your child have any problems with moving?
Yes ◯ No ◯
If yes, please provide some details:

20. Does your child have any problems with using their hands?
Yes ◯ No ◯
If yes, please provide some details:

21. Does your child have any problems with their speech?
Yes ◯ No ◯
If yes, please provide some details:

22. Does your child have any problems with their vision?
Yes ◯ No ◯
If yes, please provide some details:

23. Does your child have any problems with their hearing?
Yes ◯ No ◯
If yes, please provide some details:

24. Does your child have any problems with their behaviour?
Yes ◯ No ◯
If yes, please provide some details:

25. Does your child have any learning difficulties?
Yes ◯ No ◯
If yes, please provide some details:

Thank you for filling in the form. The following section asks how you are happy for us to use the data or to contact you:

(A) If we need to clarify some of your answers, would you be willing to be contacted?

Yes ◯ No ◯ Please initial the box here ☐

Address, Telephone number &/or email:

For peer review only

For peer review only - http://bmjopen.bmj.com/site/about/guidelines.xhtml

Study No: 12351

____

____

(B) Most admissions and GP visits in Wales are stored in computer databases called NCCHD or PEDW. As part of this study, we would also like to study how children in Wales have used their GPs or have had admission to hospitals. Would you be happy for us to use your son's or daughter's records on these databases?

Yes ◯ No ◯ Please initial the box here ☐

(C) We may plan similar studies in the future, would you be willing to be contacted in the future?

Yes ◯ No ◯ Please initial the box here ☐

Name of Child	Name of Parent/Guardian	Date	Signature

Thank you very much for taking the time to fill in the form and for contributing to our research. Could you please send the form to:

Dr Martin Edwards,
Room UGT156
Department of Child Health
School of Medicine
Cardiff University
University Hospital of Wales
Heath Park
Cardiff CF14 4XN

In the enclosed self-addressed envelope.

For peer review only - http://bmjopen.bmj.com/site/about/guidelines.xhtml

Using a **PENCIL** fill in the circles like this

○ → ●

DO NOT tick or cross the circles.

An eraser can be used to rectify mistakes.

Study No:

Health questionnaire for school age children (>5 years old)

Today's date ____________________2012

Person completing the questionnaire: Mother ○ Father ○ Other ○

Date of birth of child: ______/_________/________

1. Has your child ever had wheezing or whistling in their chest at any time in the past?

   Yes ○ No ○ Unsure ○

IF YOU HAVE ANSWERED "NO" PLEASE GO TO QUESTION 10.

2. Has your child had wheezing or whistling in the chest in the last 12 months?

   Yes ○ No ○ Unsure ○

IF YOU HAVE ANSWERED "NO" PLEASE GO TO QUESTION 10.

3. How many attacks of wheezing has your child had in the last 12 months?

   None ○ 1 to 3 ○ 4 to 12 ○ more than 12 ○

4. In the last 12 months, how often, on average, has your child's sleep been disturbed due to wheezing?

   Never woken with wheezing ○

   Less than one night per week ○

   One or more nights per week ○

5. In the last 12 months, has wheezing ever been severe enough to limit your child's speech to only one or two words at a time between breaths?

   Yes ○ No ○

6. Has a doctor ever told you that your child has asthma?

   Yes ○ No ○

7. In the last 12 months, has your child's chest sounded wheezy during or after exercise?

   Yes ○ No ○

8. In the last 12 months, has your child ever used any regular asthma inhalers (pumps) or medicines?

   Yes ○ No ○

   If yes, please provide the name (or colour of the pump) with details of how often used:

   ____

   ____

For peer review only - http://bmjopen.bmj.com/site/about/guidelines.xhtml

Study No:

9. In the last 12 months, has your child had a dry cough at night, apart from a cough associated with a cold or chest infection?
   Yes ◯ No ◯

10. A. Does the child's mother smoke cigarettes?
   Yes ◯ No ◯
   If yes, how many per day? 1 to 10 ◯ 11 to 20 ◯ more than 20 ◯

   If the child's mother smokes did she smoke during the pregnancy? Yes ◯ No ◯

10.B. Does the child's father smoke cigarettes?
   Yes ◯ No ◯

   If yes, how many per day? 1 to 10 ◯ 11 to 20 ◯ more than 20 ◯

11. Do any other household members smoke cigarettes? Yes ◯ No ◯

   If yes, how many per day for the whole household? 1 to 10 ◯
   11 to 20 ◯
   more than 20 ◯
   (please add up all the cigarettes which are smoked by everyone living in the same household including the mother).

12. In the last 12 months, has your child had any chest infections?

   Yes ◯ No ◯

13. In the last 12 months, how many chest infections has your child had?

   None ◯ 1 to 3 ◯ 4 to 10 ◯ more than 10 ◯

14. In the last 12 months, how many courses of antibiotics has your child had?

   None ◯ 1 to 3 ◯ 4 to 10 ◯ more than 10 ◯

15. In the last 12 months, how many admissions (overnight or longer) has your child had to hospital for breathing problems?

   None ◯ 1 to 3 ◯ 4 to 10 ◯ more than 10 ◯

16. Is there a family history of

	Yes	No
a. Asthma	◯	◯
b. Eczema	◯	◯
c. Hayfever	◯	◯
d. Allergies	◯	◯

17. Has your child ever been diagnosed with any breathing problems (e.g. asthma, CF, TB etc.)?

   Yes ◯ No ◯

   If yes, please provide some details:

   ____

   ____

For peer review only - http://bmjopen.bmj.com/site/about/guidelines.xhtml

Study No:

18. Has your child ever been diagnosed with any other conditions (e.g. diabetes, epilepsy etc.)?

Yes ◯ No ◯

If yes, please provide some details:

____

____

19. Is your child on any medication?

Yes ◯ No ◯

If yes, please provide some details:

____

____

20. Does your child take part in any physical activity such as dancing, cycling or swimming?

Yes ◯ No ◯

If yes, please provide some details of how often and for how long:

____

____

21. Does your child have any learning problems?

Yes ◯ No ◯

If yes, please provide some details:

____

____

22. Does your child have any problems with their behaviour?

Yes ◯ No ◯

If yes, please provide some details:

____

____

23. Does your child have an educational statement?

Yes ◯ No ◯

If yes, please provide some details:

____

____

24. Does your child have any problems with moving?

Yes ◯ No ◯

If yes, please provide some details:

____

____

For peer review only

For peer review only - http://bmjopen.bmj.com/site/about/guidelines.xhtml

Study No:

25. Does your child have any problems with writing or using their hands?

Yes ◯ No ◯

If yes, please provide some details:

___

26. Does your child have any problems with speech?

Yes ◯ No ◯

If yes, please provide some details:

___

___

27. Does your child have any problems with their vision?

Yes ◯ No ◯

If yes, please provide some details:

___

___

28. Does your child have any problems with their hearing?

Yes ◯ No ◯

If yes, please provide some details:

___

___

29. Does your child have any problems with feeding?

Yes ◯ No ◯

If yes, please provide some details:

___

___

Thank you for filling in the form. The following section asks how you are happy for us to use the data or to contact you:

(A) If we need to clarify some of your answers, would you be willing to be contacted?

Yes ◯ No ◯ Please initial the box here ☐

Address, Telephone number &/or email:

___

___

For peer review only - http://bmjopen.bmj.com/site/about/guidelines.xhtml
For peer review only

Study No:

(B) Most admissions and GP visits in Wales are stored in computer databases called NCCHD or PEDW. As part of this study, we would also like to study how children in Wales have used their GPs or have had admission to hospitals. Would you be happy for us to use your son's or daughter's records on these databases?

Yes ○ No ○ Please initial the box here ☐

(C) We may plan similar studies in the future, would you be willing to be contacted in the future?

Yes ○ No ○ Please initial the box here ☐

Name of Child	Name of Parent/Guardian	Date	Signature

Thank you very much for taking the time to fill in the form and for contributing to our research. Could you please send the form to:

Dr Martin Edwards,
Room UGT156
Department of Child Health
School of Medicine
Cardiff University
University Hospital of Wales
Heath Park
Cardiff CF14 4XN

In the enclosed self-addressed envelope.

For peer review only

For peer review only - http://bmjopen.bmj.com/site/about/guidelines.xhtml

Supplement 3. Comparison of responders and non-responders in the original study, RANOPs.

Measure	Responders n=7,129	Non-Responders n=19,593	P value
Preterm	4264 (59.9%)	9080 (46.4%)	<0.001
Birthweight centile			0.606
<10th	1879 (9.6%)	666 (9.4%)	
10th-90th	5478 (76.9%)	15,091 (77.1%)	
>90th	976 (13.7%)	2603 (13.3%)	
Male Sex	3846 (54.0%)	10,659 (54.5%)	0.523
Living in most deprived 50%	2736 (39.5%)	10,286 (53.8%)	<0.001

Numbers are number (%) or mean (SD) as appropriate.

For peer review only

Supplement 4. Multiple imputation model

Imputation was based on the following distributions. 50 imputed sets of data were used and combined using Rubin's rules.[1]

**Variable**	**Imputation command**	**% missing**
Speech Problems	-	0 (0.0%)
Learning Difficulties	logit	24 (0.3%)
Behaviour Problems	logit	41 (0.6%)
Movement Problems	logit	13 (0.2%)
Hand Problems	logit	4 (0.1%)
Maternal Age (years)	ologit	779 (11.1%)
Sex	-	0 (0.0%)
Single/Multiple pregnancy	-	0 (0.0%)
Mode of Delivery	mlogit	2913 (41.6%)
WIMD measure	regress	200 (2.9%)
Smoking Status	Logit	279 (4.0%)
Gestational age at birth	-	0 (0.0%)
Age at survey (years)	ologit	1 (0.01%)
Birthweight centile	-	0 (0.0%)

1. Rubin DB. Inference and missing data. Biometrika. 1976;63:581-92.

For peer review only

STROBE Statement—checklist of items that should be included in reports of observational studies

	Item No.	Recommendation	Page No.	Relevant text from manuscript
**Title and abstract**	1	(*a*) Indicate the study's design with a commonly used term in the title or the abstract	1	
		(*b*) Provide in the abstract an informative and balanced summary of what was done and what was found	2-3	
**Introduction**				
Background/rationale	2	Explain the scientific background and rationale for the investigation being reported	5-6	
Objectives	3	State specific objectives, including any prespecified hypotheses	6	
**Methods**				
Study design	4	Present key elements of study design early in the paper	6-7	
Setting	5	Describe the setting, locations, and relevant dates, including periods of recruitment, exposure, follow-up, and data collection	6-7	
Participants	6	(*a*) *Cohort study*—Give the eligibility criteria, and the sources and methods of selection of participants. Describe methods of follow-up *Case-control study*—Give the eligibility criteria, and the sources and methods of case ascertainment and control selection. Give the rationale for the choice of cases and controls *Cross-sectional study*—Give the eligibility criteria, and the sources and methods of selection of participants	7-8	
		(*b*) *Cohort study*—For matched studies, give matching criteria and number of exposed and unexposed *Case-control study*—For matched studies, give matching criteria and the number of controls per case	7-8	
Variables	7	Clearly define all outcomes, exposures, predictors, potential confounders, and effect modifiers. Give diagnostic criteria, if applicable	8-9	

For peer review only - http://bmjopen.bmj.com/site/about/guidelines.xhtml

Data sources/ measurement	8*	For each variable of interest, give sources of data and details of methods of assessment (measurement). Describe comparability of assessment methods if there is more than one group	7	
Bias	9	Describe any efforts to address potential sources of bias	10	
Study size	10	Explain how the study size was arrived at	N/A	Supplement 1

Continued on next page

Quantitative variables	11	Explain how quantitative variables were handled in the analyses. If applicable, describe which groupings were chosen and why	9-10
Statistical methods	12	(*a*) Describe all statistical methods, including those used to control for confounding	9-10
		(*b*) Describe any methods used to examine subgroups and interactions	9-10
		(*c*) Explain how missing data were addressed	10
		(*d*) *Cohort study*—If applicable, explain how loss to follow-up was addressed *Case-control study*—If applicable, explain how matching of cases and controls was addressed *Cross-sectional study*—If applicable, describe analytical methods taking account of sampling strategy	N/A
		(*e*) Describe any sensitivity analyses	10
**Results**			
Participants	13*	(a) Report numbers of individuals at each stage of study—eg numbers potentially eligible, examined for eligibility, confirmed eligible, included in the study, completing follow-up, and analysed	10-11
		(b) Give reasons for non-participation at each stage	10-11
		(c) Consider use of a flow diagram	N/A
Descriptive data	14*	(a) Give characteristics of study participants (eg demographic, clinical, social) and information on exposures and potential confounders	11
		(b) Indicate number of participants with missing data for each variable of interest	10
		(c) *Cohort study*—Summarise follow-up time (eg, average and total amount)	N/A
Outcome data	15*	*Cohort study*—Report numbers of outcome events or summary measures over time	12

For peer review only - http://bmjopen.bmj.com/site/about/guidelines.xhtml

		Case-control study—Report numbers in each exposure category, or summary measures of exposure	N/A
		Cross-sectional study—Report numbers of outcome events or summary measures	N/A
Main results	16	(*a*) Give unadjusted estimates and, if applicable, confounder-adjusted estimates and their precision (eg, 95% confidence interval). Make clear which confounders were adjusted for and why they were included	10-11
		(*b*) Report category boundaries when continuous variables were categorized	10-13
		(*c*) If relevant, consider translating estimates of relative risk into absolute risk for a meaningful time period	N/A

Continued on next page

Other analyses	17	Report other analyses done—eg analyses of subgroups and interactions, and sensitivity analyses	14
**Discussion**			
Key results	18	Summarise key results with reference to study objectives	15-17
Limitations	19	Discuss limitations of the study, taking into account sources of potential bias or imprecision. Discuss both direction and magnitude of any potential bias	15-17
Interpretation	20	Give a cautious overall interpretation of results considering objectives, limitations, multiplicity of analyses, results from similar studies, and other relevant evidence	15-17
Generalisability	21	Discuss the generalisability (external validity) of the study results	15-17
**Other information**			
Funding	22	Give the source of funding and the role of the funders for the present study and, if applicable, for the original study on which the present article is based	17

*Give information separately for cases and controls in case-control studies and, if applicable, for exposed and unexposed groups in cohort and cross-sectional studies.

Note: An Explanation and Elaboration article discusses each checklist item and gives methodological background and published examples of transparent reporting. The STROBE checklist is best used in conjunction with this article (freely available on the Web sites of PLoS Medicine at http://www.plosmedicine.org/, Annals of Internal Medicine at http://www.annals.org/, and Epidemiology at http://www.epidem.com/). Information on the STROBE Initiative is available at www.strobe-statement.org.

For peer review only
